# Development and validation of a two-gene urine DNA methylation assay for noninvasive prostate cancer detection: a prospective multicenter study

**DOI:** 10.1186/s43556-026-00528-y

**Published:** 2026-08-07

**Authors:** Dingwen Liu, Lian Tang, Wei Xiong, Ran Xu, Qing Zhou, Genming Xu, Zhen Li, Wei Tang, Jinru Feng, Xiaowei Zeng, Jinlei Liu, Chao Li, Lei Chen, Long Wang

**Affiliations:** 1 Department of Urology, The Third Xiangya Hospital, Central South University, Changsha, Hunan 410013 China; 2 Hunan Yearth Biotechnology Co., Ltd., Changsha, 410205 China; 3 Department of Urology, The Second Xiangya Hospital of Central South University, Changsha, Hunan 410011 China; 4 Department of Andrology, The First Hospital of Hunan University of Chinese Medicine, Changsha, 410007 China; 5 Department of Urology, Hunan Provincial People’s Hospital, The First Affiliated Hospital of Hunan Normal University, Changsha, Hunan China; 6 Department of Urology, Zhuzhou Central Hospital, Zhuzhou, Hunan 412007 China

**Keywords:** Prostate cancer, Urine DNA methylation, Noninvasive diagnosis, Biomarker discovery, Clinical decision support

## Abstract

Prostate-specific antigen (PSA) screening lacks specificity, particularly within the 4–10 ng/mL diagnostic gray zone, leading to unnecessary prostate biopsies and overtreatment. Here, we developed and validated a streamlined urine DNA methylation assay for non-invasive prostate cancer (PCa) detection and biopsy decision-making. Tissue-based Methylated DNA Immunoprecipitation Sequencing (MeDIP-seq) was integrated with large-scale public methylome cohorts to identify PCa-specific hypermethylated regions. Candidate markers were further refined by targeted bisulfite sequencing (TBS) and translated into quantitative PCR (qPCR) assays using post-prostatic massage urine samples. In a retrospective development cohort of 348 participants, stepwise marker selection and logistic regression modelling generated a compact two-gene methylation assay comprising *ADD3_2* and *GSX2_2.* The model was then evaluated in an independent prospective multicenter cohort of 200 participants. The assay achieved robust diagnostic performance, with areas under curve (AUC) of 0.86 in the development cohort and 0.91 in the validation cohort, while maintaining specificity above 90%. Performance was preserved in the PSA gray zone and for clinically significant PCa (csPCa). Decision curve analysis (DCA) indicated that integrating this tool could safely avert approximately 55% of unnecessary biopsies without compromising cancer detection. This simple qPCR-based two-gene urine methylation assay provides a scalable, cost-effective and non-invasive complement to PSA-based PCa screening.

**Supplementary Information:**

The online version contains supplementary material available at 10.1186/s43556-026-00528-y.

## Introduction

Prostate cancer (PCa) is one of the most common solid malignancies in men and ranks as the fifth leading cause of cancer-related mortality worldwide, with approximately 1.4 million new cases and over 397,000 deaths reported in 2022 [1]. In China, the mortality-to-incidence ratio remains notably high (0.44), compared with that in the United States (0.15), largely due to late-stage diagnosis and a higher proportion of patients presenting with advanced Gleason scores [2]. With an estimated annual increase in incidence of 2.6%, there is an urgent need for more efficient, accurate, and cost-effective strategies for early detection of PCa.

At present, serum prostate-specific antigen (PSA) testing remains the most widely used screening tool for PCa. However, its clinical utility is constrained by limited specificity, particularly within the diagnostic “gray zone” (4–10 ng/mL), where only 25%–40% of biopsies are positive. Even at PSA levels above 20 ng/mL, the positive predictive value is approximately 70% [3, 4]. Consequently, a substantial proportion of patients with elevated PSA do not harbor malignancy, leading to unnecessary biopsies and potential overtreatment [5]. These limitations highlight the need for novel biomarkers that can improve diagnostic precision and support more reliable risk stratification.

Epigenetic alterations, especially DNA methylation abnormalities such as CpG island hypermethylation, are a well-established hallmark of prostate carcinogenesis. Several methylation-based biomarkers, including *GSTP1*, *APC*, *RASSF1A*, and *CDH1*, have been identified in tumor tissues and further adapted for liquid biopsy applications. Among these, post-digital rectal examination (DRE) urine has emerged as a particularly promising non-invasive specimen due to its reproducibility and enrichment of prostate-derived DNA [6]. In recent years, multi-gene urine methylation assays (e.g., epiCaPture) have demonstrated encouraging diagnostic performance for clinically significant PCa (csPCa) [7–10]. However, most existing panels rely on large gene sets, which increases assay complexity, cost, and inter-population variability, limiting their widespread clinical adoption [11, 12].

To address these challenges, this study aimed to develop and validate a highly simplified, non-invasive urine DNA methylation assay to refine clinical decision-making. We hypothesized that a concise epigenetic signature derived from PCa-specific methylation alterations could improve diagnostic specificity while preserving clinical practicality and reducing assay complexity. Leveraging tissue-based methylome profiling and public multi-omics datasets, we prioritized candidate methylation markers with high disease specificity and minimal background signals from benign or non-prostatic conditions. Through stepwise marker selection and multicenter clinical validation, we identified two robust methylation biomarkers, *ADD3_2* and *GSX2_2*, and developed a parsimonious two-gene diagnostic model suitable for routine clinical use. In an independent prospective multicenter cohort, the assay achieved an AUC of 0.91 while maintaining specificity above 90%, with the potential to reduce approximately 55% of unnecessary biopsies without compromising the detection of clinically significant prostate cancer.

## Results

### Patient characteristics

**Fig. 1:**
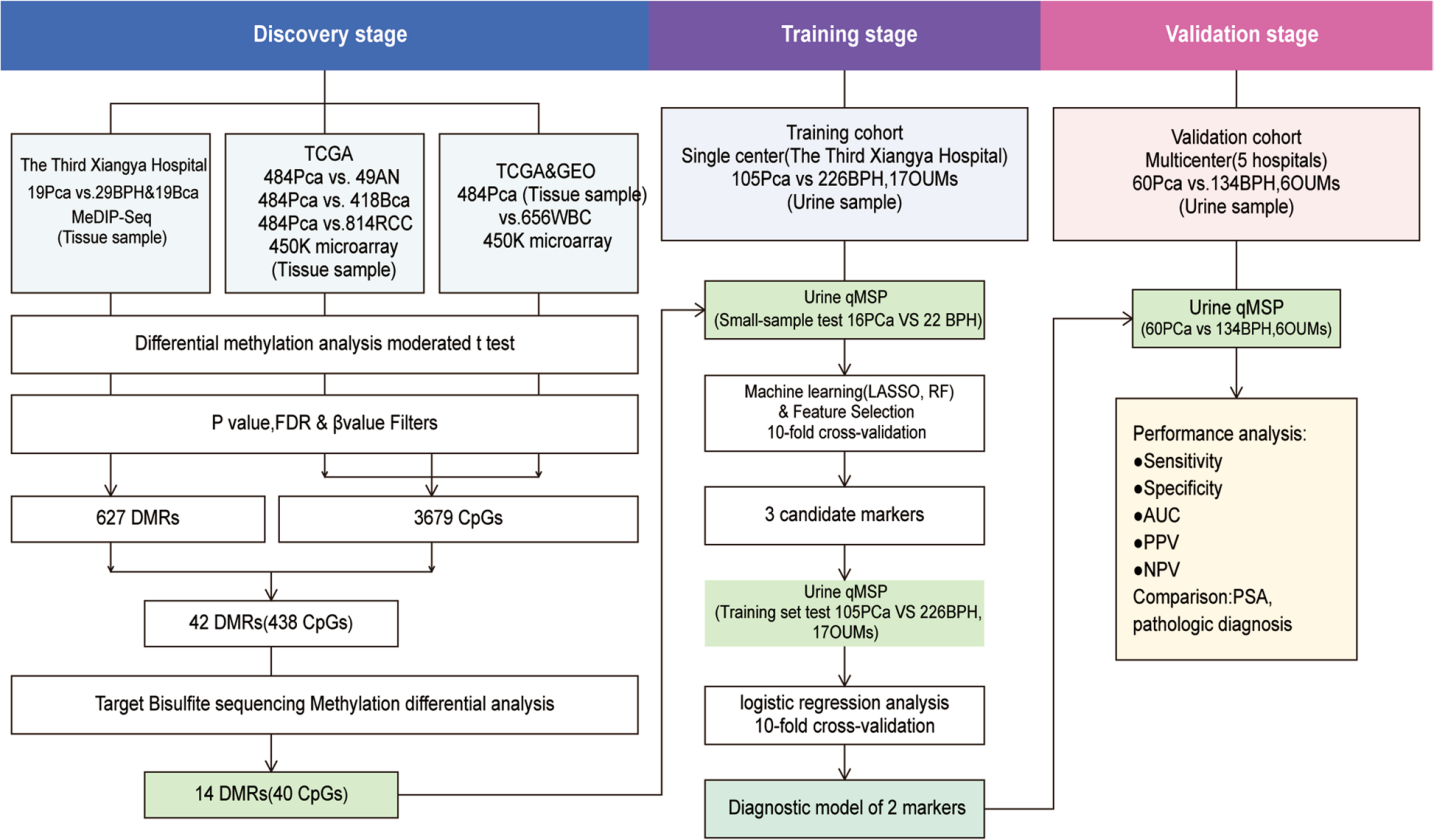
Workflow indicating the study design. MeDIP‑seq, Methylated DNA immunoprecipitation sequencing; TCGA, The Cancer Genome Atlas; GEO, Gene Expression Omnibus; PCa, prostate cancer; BCa, bladder cancer; BPH, benign prostatic hyperplasia; OUMs, other urological malignancies; FDR, false discovery rate; DMRs, differential methylation regions; qMSP, quantitative methylation specific PCR; LASSO, Least Absolute Shrinkage and Selection Operator; PSA, prostate‑specific antigen; PPV, positive predictive value; NPV, negative predictive value; AUC, area under curve

To establish a high-quality clinical framework for validating our non-invasive diagnostic assay, we first comprehensively analyzed the demographic and clinical characteristics of all enrolled participants. In the initial marker discovery phase, public datasets from The Cancer Genome Atlas (TCGA) and Gene Expression Omnibus (GEO) were utilized, comprising 484 PCa tissues, 49 adjacent normal (AN) tissues, 418 bladder cancer (BCa) tissues, 814 renal cell carcinoma (RCC) tissues, and 656 normal peripheral blood leukocyte samples. Concurrently, a clinical tissue verification cohort from the Third Xiangya Hospital, which included 19 PCa tissues, 29 benign prostatic hyperplasia (BPH) tissues, and 19 BCa tissues, was enrolled to complement the marker discovery phase (Fig. 1). The detailed demographic and clinical characteristics of the tissue cohort are comprehensively summarized in Table S1.

For the clinical noninvasive validation utilizing urine samples, a total of 548 participants were enrolled and completed the analysis in this study, comprising 348 in the training cohort and 200 in the validation cohort. Specifically, the training cohort included 106 PCa patients, 225 BPH control individuals, and 17 patients with other urological malignancies (OUMs). The validation cohort consisted of 60 PCa patients, 134 BPH control individuals, and 6 patients with OUMs. The median age of PCa patients was 71 years in the training cohort and 72 years in the validation cohort, while the corresponding median age for BPH patients was 70 years in both cohorts. In the training and validation cohorts, the median total PSA levels of PCa patients were 22.41 ng/mL and 23.65 ng/mL, respectively, whereas those for BPH patients were 5.73 ng/mL and 6.65 ng/mL (Table 1). Among all PCa patients, 4.22% were classified as Grade Group (GG) 1, 15.66% as GG2, and 80.12% as GG ≥ 3.

**Table 1:** Subject baseline demographics and clinical characteristics in the training cohort and validation cohort

Characteristics	Training cohort			Validation cohort		
	Case	Control		Case	Control	
	PCa (*n* = 106)	BPH (*n* = 225)	OUMs (*n* = 17)	PCa (*n* = 60)	BPH (*n* = 134)	OUMs (*n* = 6)
Age						
Median (IQR)	71 (66–74)	70 (62–75)	61 (56–70)	72 (64.5–75.5)	70 (65–76)	67.5 (64, 76.25)
PSA						
Median (IQR)	22.41 (8.28–76.62)	5.73 (2.29–14.40)	3.04 (1.27–9.2)	23.65 (9.49–78.92)	6.65 (3.18, 14.83)	1.96 (1.10, 3.76)
Gleason						
GG1	3(2.83%)			4(6.67%)		
GG2	17(16.04%)			9(15.00%)		
GG3	23(21.70%)			11(18.33%)		
GG4	25(23.58%)			15(25.00%)		
GG5	38(35.85%)			21(35.00%)		
Renal carcinoma			10(58.82%)			2(33.33%)
Urothelial carcinoma			7(41.18%)			4(66.67%)

*PCa* prostate cancer, *BPH* benign prostatic hyperplasia, *OUMs* other urological malignancies, *GG* Grade Group

### Discovery of DNA methylation markers to distinguish PCa from normal tissue

**Fig. 2:**
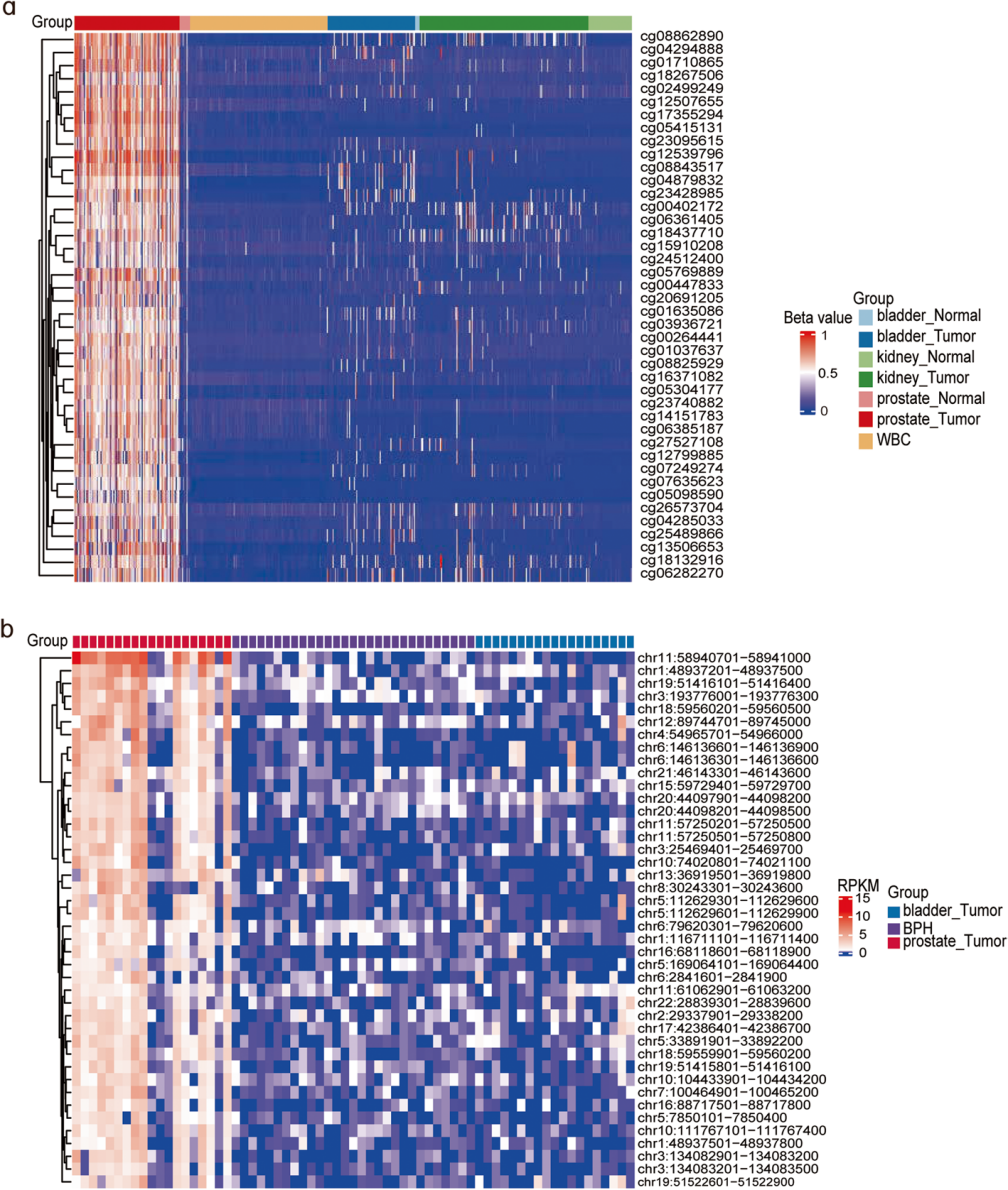
Identification of DMRs distinguishing PCa from BPH and BCa tissues. **a** Unsupervised hierarchical clustering of 42 DMRs among PCa (*n* = 484), adjacent normal prostate tissues (*n* = 49), BCa (*n* = 418), normal bladder tissues (*n* = 21), RCC (*n* = 814), normal kidney tissues (*n* = 207), and normal peripheral blood leukocytes (*n* = 656) in the TCGA and GEO cohorts. **b** Unsupervised hierarchical clustering of 42 DMRs among PCa (*n* = 19), BPH (*n* = 29), and BCa tissues (*n* = 19) in the Third Xiangya Hospital cohort. DMRs, differential methylation regions; PCa, prostate cancer; BCa, bladder cancer; BPH, benign prostatic hyperplasia; RCC, renal cell carcinoma; TCGA, The Cancer Genome Atlas; GEO, Gene Expression Omnibus; RPKM, reads per kilobase of transcript per million mapped reads

To identify highly specific epigenetic targets capable of distinguishing prostate malignancy from benign conditions and confounding urological tumors, we executed a rigorous, multi-stage biomarker discovery pipeline combining high-throughput tissue sequencing with multi-omics public data mining. The overarching study workflow is schematically presented in Fig. 1. To identify robust DNA methylation biomarkers tailored for highly specific PCa detection, we initially performed Methylated DNA Immunoprecipitation Sequencing (MeDIP-seq) on a discovery cohort from the Third Xiangya Hospital, comprising PCa (*n* = 19), BPH (*n* = 29), and BCa (*n* = 19) samples. To ensure pan-cancer specificity and eliminate leukocyte-associated confounding, these profiles were cross-referenced with large-scale public datasets. Specifically, we leveraged transcriptomic/epigenomic data from TCGA—comparing PCa (*n* = 484) against AN (*n* = 49), RCC (*n* = 814), and BCa (*n* = 418)—and integrated 656 normal leukocyte methylomes from the GEO database (GSE40279) to mitigate systemic noise from immune infiltration. Applying stringent statistical thresholds, differential DNA methylation analysis yielded 627 differential methylation regions (DMRs) within our institutional cohort and 3,679 PCa-specific CpG sites across the TCGA and GEO landscapes (Fig. S1). Intersecting these multi-omic layers—defined as CpG sites residing within or within 100 bp boundaries of the identified DMRs—uncovered 42 high-confidence candidate DMRs demonstrating strict PCa specificity (Fig. 2, Table S2). These 42 DMRs exhibited high specificity in PCa tissues (Fig. S2-8). For downstream experimental validation, we designed 162 primer pairs spanning 438 constituent CpG sites across these 42 candidate regions using Methyl Primer Express v1.0 (Table S3). Subsequent targeted bisulfite sequencing (TBS) of the institutional tissue cohorts, followed by differential analysis via the limma R package, successfully validated 40 core CpG sites across 14 master DMRs. These loci exhibited a striking hypermethylation profile exclusive to PCa tissues, while remaining meticulously unmethylated in non-malignant controls (Table S4). Taken together, these rigorous multi-stage screening phases underscore the potent diagnostic potential of these epigenetic markers in differentiating PCa from confounding non-malignant and heterologous neoplastic conditions.

### Development of a methylation diagnostic model for PCa

To compress the tissue-derived epigenetic signature into a clinically scalable and cost-effective screening tool, we established a targeted quantitative real-time PCR (qPCR) assay framework and applied machine learning algorithms to construct a parsimonious diagnostic model. Based on 40 differentially methylated CpG sites, 23 primer–probe pairs were designed for qPCR validation (Table S5). From a training cohort of 348 cases, 16 PCa and 22 BPH samples were randomly selected as a small-sample screening subset for biomarker screening. In urine-based qPCR analyses, 19 probes effectively distinguished PCa from non-PCa samples, showing markedly higher methylation in PCa and consistently lower levels in non-PCa cases, indicating strong diagnostic specificity. From this small-sample screening subset, the three top-performing genes—*ADD3_2*, *GSX2_2*, and *CYBA_1*—were selected to construct a predictive model in the training cohort, which encompassed 106 PCa cases and 242 controls (225 BPH, 17 OUMs) (Table S6). During model optimization, the two-gene model (*ADD3_2* and *GSX2_2*) was selected over a three-gene panel because it demonstrated superior overall diagnostic accuracy in the training cohort (85.06% vs. 83.05%), despite a marginal loss in sensitivity (Table S7). This parsimonious two-gene signature offers higher clinical accuracy while reducing assay complexity and cost. The diagnostic cutoff of 0.4488 was derived strictly from the training cohort via the maximum Youden index.

**Fig. 3:**
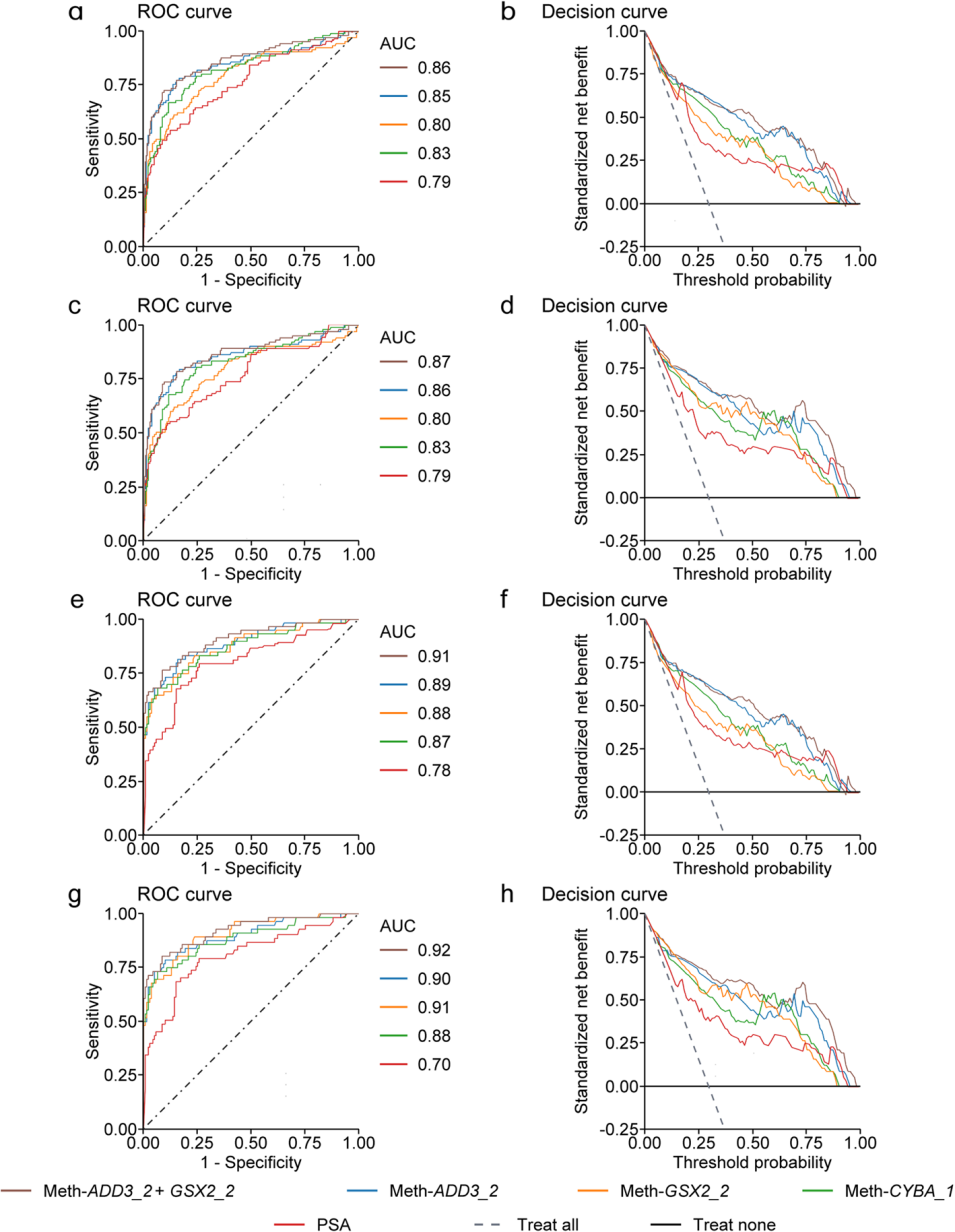
The ROC analysis and DCA were conducted to evaluate the performance of various risk models in both the training and validation cohorts. ROC analysis for any cancer (training** a**; validation **c**) and ≥ GG2 detection (training **e**; validation **g**). DCA for any cancer (training **b**; validation **d**) and ≥ GG2 detection (training **f**; validation **h**). ROC, receiver operating characteristic; DCA, decision curve analysis; GG, Grade Group. AUC, area under curve; PSA, prostate‑specific antigen

The optimal two-gene panel (*ADD3_2* + *GSX2_2*) achieved 85.06% accuracy, 72.64% sensitivity, 90.50% specificity, and an AUC of 0.86, significantly outperforming individual markers and conventional PSA (Fig. 3, Table S8). Notably, the diagnostic robustness of this model was sustained across clinical PSA subgroups; specifically, within the gray-zone range (4–10 ng/mL), it yielded an accuracy of 86.05% and a high specificity of 92.19% (Table S8). Furthermore, decision curve analysis (DCA) demonstrated a superior net clinical benefit for the panel over either single genes or PSA across a wide spectrum of threshold probabilities (Fig. 3), underscoring its translational potential as a refined, non-invasive diagnostic tool for PCa.

### Validation of the model for cancer diagnosis

To rigorously assess the generalizability, stability, and reproducibility of the finalized two-gene diagnostic model in real-world clinical environments, we subjected the locked assay algorithm and its predefined 0.4488 decision cutoff to independent prospective evaluation. The model demonstrated robust diagnostic stability, yielding an AUC of 0.91, accompanied by a sensitivity of 76.67%, a specificity of 90.71%, a positive predictive value (PPV) of 78.12%, and a negative predictive value (NPV) of 90.14% (Table 2, Table S9), while consistently maintaining a superior net clinical benefit. At an optimized decision threshold of 0.4488, implementing this model could potentially avert up to almost 55.00% of unnecessary biopsies in men suspected of harboring PCa (Fig. S9, Table S10). Crucially, this diagnostic efficacy sustained its performance within the stringent PSA gray zone (4–10 ng/mL), presenting an accuracy of 87.04% and a high specificity of 90.00% (Table 2).

**Table 2:** The clinical performance of the diagnostic model (*ADD3_2*, *GSX2_2*) in different PSA levels in the validation cohort

	**All**		**PSA < 4**		**4 ≤ PSA ≤ 10**		**PSA > 10**	
	**Case**	**Control**	**Case**	**Control**	**Case**	**Control**	**Case**	**Control**
Predicted positive	46	13	2	8	2	5	42	0
Predicted negative	14	127	0	51	2	45	12	31
Sensitivity(%)	76.67 (66.67- 86.67)		75 (25- 100)		50 (0- 100)		78.85 (67.31- 88.46)	
Specificity(%)	90.71 (85.71- 95)		87.04 (77.78- 94.44)		90 (82- 96)		97.22 (91.67- 100)	
PPV(%)	78.12 (69.22- 87.27)		30.77 (12.5- 57.14)		28.57 (0- 60)		97.67 (92.68- 100)	
NPV (%)	90.14 (86.01- 94.12)		97.96 (94- 100)		95.74 (91.84- 100)		76.09 (67.31- 85.71)	
Accuracy(%)	86.5 (82- 91)		86.21 (77.59- 94.83)		87.04 (77.78- 94.44)		86.36 (79.55- 93.18)	
AUC	0.91 (0.86- 0.96)		0.87 (0.634- 1)		0.76 (0.56- 0.97)		0.95 (0.91- 0.99)	

*PSA* prostate‑specific antigen, *PPV* positive predictive value, *NPV* negative predictive value, *AUC* area under curve

### Identification of clinically significant cancer

To further investigate the clinical utility of the two-gene model, we evaluated its diagnostic capacity specifically for the identification of csPCa and analyzed its performance stratification across increasing disease severity. Accurate identification of csPCa is paramount for optimizing patient stratification. We therefore evaluated the competency of our model in discriminating csPCa from non-malignant conditions. In the training cohort, the panel correctly classified 73.79% of ≥ GG2 cases, yielding a specificity of 90.50% and an overall accuracy of 85.51%. Notably, within the critical PSA gray zone (4–10 ng/mL), the sensitivity, specificity, and diagnostic accuracy reached 71.43%, 92.19%, and 87.06%, respectively (Table 3). This diagnostic efficacy was robustly reproduced in the validation cohort for ≥ GG2 detection, exhibiting a sensitivity of 80.36%, a specificity of 90.71%, a PPV of 77.78%, and an NPV of 92.09%. Furthermore, among patients with advanced PSA levels (> 10 ng/mL), the model’s performance escalated to a sensitivity of 83.67%, a specificity of 97.22%, and an overall accuracy of 89.41% (Table S11).

**Table 3:** The clinical performance of the diagnostic model (*ADD3_2*, *GSX2_2*) in different PSA levels of high-grade cancer (≥ GG2) in the training cohort

	**All**		**PSA < 4**		**4 ≤ PSA ≤ 10**		**PSA > 10**	
	**Case**	**Control**	**Case**	**Control**	**Case**	**Control**	**Case**	**Control**
Predicted positive	76	23	2	10	15	5	59	8
Predicted negative	27	219	2	110	6	59	19	50
Sensitivity(%)	73.79 (65.05- 81.55)		50 (0- 100)		71.43 (52.38- 90.48)		75.64 (65.38- 84.62)	
Specificity(%)	90.5 (86.78- 94.21)		91.67 (85.83- 95.83)		92.19 (85.94- 98.44)		86.21 (77.59- 94.83)	
PPV(%)	76.92 (69.81- 84.21)		16.67 (0- 37.5)		75 (60- 93.33)		88.06 (81.54- 95)	
NPV (%)	89.07 (85.94- 92.21)		98.21 (96.46- 100)		90.77 (85.51- 96.72)		72.46 (64.94- 80.95)	
Accuracy(%)	85.51 (81.74- 89.28)		90.32 (84.68- 95.16)		87.06 (80- 94.12)		80.15 (73.53- 86.03)	
AUC	0.87 (0.821- 0.916)		0.71 (0.266- 1)		0.84 (0.727- 0.959)		0.86 (0.792- 0.92)	

*PSA* prostate‑specific antigen, *PPV* positive predictive value, *NPV* negative predictive value, *AUC* area under curve

### Clinical utility of the diagnostic model

**Fig. 4:**
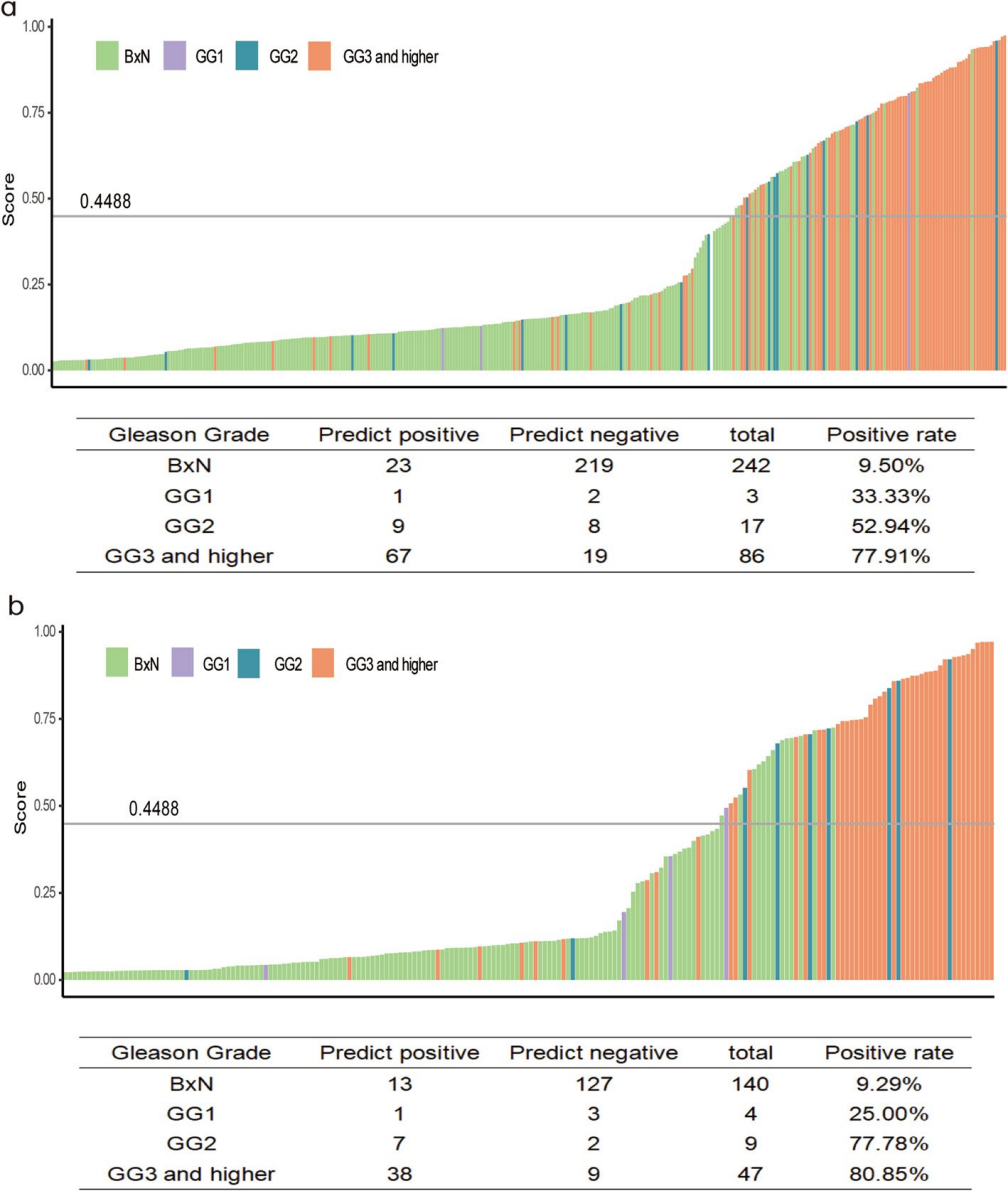
Clinical performance of the diagnostic model (*ADD3_2, GSX2_2*) in the training and validation cohorts. Waterfall plots and matching cross-tabulations illustrating the diagnostic performance of the two-gene methylation assay in the **a** training cohort and **b** validation cohort. Individual vertical bars represent single participants, color-coded by their histopathological diagnosis. GG, Grade Group; BxN, biopsy negative/benign; BPH, benign prostatic hyperplasia; OUMs, other urological malignancies

The model’s diagnostic performance across both the training and validation cohorts, stratified by the International Society of Urological Pathology (ISUP) GG, is summarized in Fig. 4. In the training cohort, the model delivered an overall diagnostic accuracy of 72.64% for general PCa detection, accurately identifying 77.91% of patients presenting with high-grade disease (≥ GG3, *n* = 84). Upon validation, the overall accuracy reached 76.67%, with the model successfully classifying 77.78% of ≥ GG2 cases and escalating to 80.85% for ≥ GG3 individuals. This progressive tracking underscores that the model’s classification efficacy scales concordantly with advancing histological malignancy.

## Discussion

Among non-invasive diagnostic modalities for PCa, a major clinical bottleneck remains the limited specificity of PSA-based screening, particularly in men undergoing initial biopsy or those within the conventional PSA gray zone. Although PSA testing is widely used, many patients with elevated PSA do not harbor malignancy, resulting in unnecessary invasive biopsies, potential overtreatment, and biopsy-related complications such as infection, hematuria, and hematochezia [13, 14]. Several advanced biomarker tests, including EPI (sensitivity 91%, specificity 34%), 4Kscore (sensitivity 81%–95%, specificity 28%–62%), SelectMDx (sensitivity 94%, specificity 46%), and PHI (sensitivity 96%, specificity 36%), have improved risk assessment but still face limitations related to specificity, clinical accessibility, or implementation cost [15–19]. Previous methylation-based urine assays have also shown promising diagnostic performance, but many rely on relatively large gene panels, which may increase assay complexity, cost, and inter-population variability [11, 12]. Therefore, there remains a clear need for a simple, accurate, and clinically scalable non-invasive assay that can refine biopsy decision-making.

In this study, we developed and multicenter-validated a urine-based DNA methylation assay tailored for the non-invasive detection of PCa, with a particular emphasis on identifying csPCa. Through systematic integration of tissue methylome profiling, public multi-omics datasets, targeted epigenetic verification, and multivariable modeling, we established a parsimonious two-gene diagnostic panel consisting of *ADD3_2* and *GSX2_2*. This model exhibited robust and reproducible diagnostic performance across both the retrospective derivation cohort and the independent prospective validation cohort. Importantly, the assay maintained favorable performance in clinically challenging subgroups, including patients within the PSA gray zone and those harboring ≥ GG2 disease. These findings support the potential value of this assay as a non-invasive test for reducing diagnostic ambiguity and improving personalized biopsy selection.

Compared with existing molecular approaches, the present two-gene model has several technical and translational advantages. Although previous methylation-based studies have reported improved diagnostic accuracy using 14-gene or 18-gene panels, the inclusion of larger marker sets inevitably increases experimental complexity and cost [11, 12]. Similarly, recently developed urine-based multi-omic algorithms may require large-scale targeted sequencing, whole-genome sequencing, mutation analysis, or copy-number profiling, which increases financial and computational burden and may hinder routine implementation [20]. In contrast, our assay uses a compact two-gene qPCR-based workflow, requires only a small amount of urine-derived DNA, and does not rely on expensive next-generation sequencing or complex bioinformatic interpretation. This streamlined design may facilitate assay standardization, reduce cost, and improve scalability for routine clinical practice.

The biological rationale for this assay is also supported by the inherent stability and tumor specificity of DNA methylation alterations. In contrast to expression-based or protein-based biomarkers, which may reflect downstream or dynamic changes in tumor biology, aberrant DNA methylation is widely regarded as a stable and early epigenetic event during prostate carcinogenesis [21]. Tumor-specific methylation signals can be maintained during disease progression and detected in prostate-derived DNA released into post-prostatic massage urine, providing a biologically robust foundation for non-invasive PCa detection [7, 22]. This may partly explain the favorable diagnostic performance observed in patients within the PSA gray zone and supports the integration of urine-based methylation assays into existing PCa diagnostic workflows.

From a clinical perspective, the primary value of this urine-based methylation assay lies in its potential role as a pre-biopsy decision-support tool. DCA indicated that incorporating the model into clinical workflows could provide additional net clinical benefit across relevant threshold probabilities and may help reduce unnecessary biopsies. This application may be particularly meaningful in settings where multiparametric MRI (mpMRI) is unavailable, costly, or yields equivocal findings. In such scenarios, a simple urine-based methylation assay could provide an additional layer of risk stratification before invasive biopsy. In addition, the predominance of high-grade PCa in several Chinese cohorts highlights the need for practical tools capable of identifying clinically significant disease in real-world clinical populations [23, 24]. However, comparisons with established biomarker tests should be interpreted cautiously because they are based mainly on historical literature rather than direct head-to-head prospective evaluation.

Several limitations merit consideration. Firstly, at the optimal risk cutoff, the model presented a false-negative rate of 23.3% (sensitivity 76.7%) in the validation cohort. Three of these missed cases were low-grade GG1 tumors that usually warrant active surveillance rather than immediate treatment, and this trade-off is comparable to current commercial benchmarks like PHI or 4Kscore. Future studies should prospectively define clinically acceptable threshold probabilities and systematically report the number and grade distribution of missed cancers, especially missed csPCa, across different decision thresholds. Additionally, the number of patients with PSA levels of 4–10 ng/mL as well as those with GG1 and GG2 disease was relatively limited. Because high-grade tumors generally exhibit greater tumor burden and more pronounced epigenetic alterations, they may release higher levels of tumor-derived methylated DNA into urine, potentially facilitating biomarker detection. As a result, the diagnostic performance observed in the present study may partially reflect enrichment for clinically significant disease rather than the performance expected in a true population-based screening setting enriched for low-risk or indolent tumors. Finally, while our validation cohort was multicenter, it was limited to Chinese institutions, and future studies integrating other clinical variables and imaging modalities should further validate this assay in larger, more diverse populations—including different ethnic groups, healthcare systems, biopsy indications, and disease stages—to confirm its generalizability.

Collectively, our findings support the utility of a compact urine-based DNA methylation assay as a non-invasive approach for detecting PCa and csPCa. By improving specificity beyond PSA-based screening while maintaining a simple qPCR-compatible workflow, this assay has the potential to reduce unnecessary biopsies and refine current early detection strategies. Future prospective studies incorporating larger numbers of GG1 and early-stage PCa cases will be essential to further validate the performance and generalizability of this assay in lower-risk screening populations. In addition, integrating urine methylation markers with imaging modalities and clinical risk models may further refine personalized diagnostic strategies and advance precision screening paradigms for PCa.

## Material and methods

### Study design and participants

This study was conducted in two sequential phases: biomarker discovery using tissue samples and public datasets, followed by model development and multicenter prospective validation using urine samples (Fig. 1).

For biomarker discovery, we integrated methylation data from an institutional tissue cohort with two independent public datasets generated using the Illumina HumanMethylation450 BeadChip platform to identify PCa-specific markers. The institutional cohort included 19 PCa, 29 BPH, and 19 BCa tissue samples collected at the Third Xiangya Hospital between February and May 2024. Public datasets comprised the TCGA cohort, including 484 PCa cases (49 with matched normal tissues), 814 RCC samples, and 418 BCa samples, together with the GEO dataset GSE40279 containing 656 peripheral blood leukocyte samples, which were used to filter non-specific blood-derived methylation signals.

For the model development and validation phases, liquid biopsy-based evaluations were performed using post-prostatic massage urine samples. First, a retrospective single-center model development cohort was established at the Third Xiangya Hospital, comprising 106 PCa, 225 BPH, and 17 OUMs collected between June 2024 and July 2025. From this cohort, a subset of 16 PCa and 22 BPH samples was randomly selected for preliminary small-sample screening. Second, a prospective multicenter validation cohort of 60 PCa, 134 BPH, and 6 OUMs patients was recruited between August and December 2025 across five Chinese hospitals: the Third Xiangya Hospital, the Second Xiangya Hospital, the First Hospital of Hunan University of Chinese Medicine, Hunan Provincial People’s Hospital, and Zhuzhou Central Hospital. OUMs across both cohorts included RCC, renal pelvic carcinoma, BCa, and ureteral carcinoma.

The inclusion criteria for the urine-based cohorts were males aged ≥ 18 years presenting with clinical indications suggestive of PCa or confounding urological conditions. Exclusion criteria included non-fresh or insufficient urine samples, incomplete clinical records, and prior prostate-related treatments (including surgery, radiotherapy, chemotherapy, or endocrine therapy). Indications for a subsequent prostate biopsy included a total serum PSA > 10.0 ng/mL, a total serum PSA of 4.0–10.0 ng/mL accompanied by a free-to-total PSA ratio (f/t PSA) < 0.16, a PSA density (PSAD) ≥ 0.15 ng/mL/cm^3^, highly suspicious lesions on mpMRI, Prostate Imaging Reporting and Data System (PI‑RADS) score ≥ 3, or palpable abnormalities on DRE. In the prospective validation cohort, all 200 enrolled patients completed the urine-based assay. Among them, 168 patients met the clinical criteria and underwent prostate biopsy, while the remaining 32 participants who did not undergo biopsy were retained in the cohort to maintain prospective evaluation integrity (individual patient details are provided in Table S12).

### Sample processing

For the tissue-based discovery phase, genomic DNA was extracted from freshly frozen tissues using a commercial DNA extraction kit (TIANGEN BIOTECH (BEIJING) CO., LTD, catalog DP304.) following the manufacturer’s instructions. Approximately 0.5 mg of tissue was used per extraction, yielding an average of 5 µg of genomic DNA, which was subsequently stored at −80 °C.

The primary experimental materials, reagents, and instruments utilized in this study are detailed in Table S13. For the subsequent urine-based training and prospective validation phases, 50–100 mL of first-void urine was obtained after prostatic massage, prior to any invasive examination or surgical procedure. Urine samples were stored at room temperature for no longer than five days in urine preservation buffer. DNA methylation markers remained sufficiently stable during this period without compromising the detection results (Fig. S10, Table S14). DNA was extracted using the Nucleic Acid Extraction or Purification Reagents (Xingchun (Changzhou) Biotechnology Co., Ltd, catalog M15R) according to the manufacturer’s protocol. Only samples yielding more than 50 ng of DNA were considered eligible for downstream analyses; qualified DNA samples were stored at −20 °C until use.

### MeDIP-seq analysis

To screen for genome-wide methylation variations in the discovery phase, MeDIP-seq was performed on the genomic DNA extracted from the 67 tissue samples mentioned above. Genomic DNA was extracted from collected tissue samples using a commercial DNA extraction kit (TIANGEN BIOTECH (Beijing) Co., Ltd., catalog DP304) according to the manufacturer’s instructions. A total of 1 µg of genomic DNA was sheared into 200–500 bp fragments using a Covaris ultrasonicator. Pre-libraries were constructed using the NadPrep® DNA Library Preparation Kit (Nanodigmbio (Nanjing) Biotechnology Co., Ltd., catalog 1002421), followed by enrichment of methylated DNA using the MagMeDIP qPCR Kit (Diagenode, catalog C02010021). The enriched DNA was subsequently amplified using P5/P7 primers, purified, quantified, and subjected to high-throughput sequencing on the DNBSEQ-T7 platform (MGI Tech, Shenzhen, China). Raw MeDIP-seq data were quality-controlled, and low-quality reads as well as adapter sequences were removed using fastp (v0.20.0). Clean reads were aligned to the human reference genome (GRCh37/hg19) using Bowtie, and differential DNA methylation analysis was performed using the MEDIPS (v1.48.0) and limma R packages to identify DMRs between PCa and control samples.

### Multiplex PCR–based targeted DNA methylation sequencing

As an intermediate validation step within the tissue discovery phase, the identified candidate DMRs were further verified in the same 67 tissue samples using high-depth targeted sequencing. For candidate differentially methylated regions, multiplex bisulfite PCR primer pools were designed using Methyl Primer Express v1.0 (Table S3), and potential primer–dimer formation was systematically evaluated using an in-house script. Following bisulfite conversion of genomic DNA extracted from tissue samples, multiplex PCR amplification was performed for 25–33 cycles. The resulting amplicons were purified using magnetic beads and subsequently indexed with P5/P7-containing primers to generate sequencing-ready libraries. Library quality and concentration were assessed using a Qubit 3.0 fluorometer and the Agilent 2100 Bioanalyzer. Qualified libraries were sequenced on the Illumina Nova platform in paired-end 150 bp mode, achieving an average target amplicon depth of ≥ 200 ×. After quality control filtering, FASTQ files were processed using Bismark v0.20.0 for alignment and DNA methylation calling. Cytosine methylation levels were calculated as follows: methylation ratio = number of methylated reads/(number of methylated reads + number of unmethylated reads).

### Identification of PCa-specific DNA methylation signatures

During the tissue-based discovery phase, candidate DNA methylation markers were identified by comparing PCa (*n* = 19) with BPH (*n* = 29) and BCa (*n* = 19) tissues from the Third Xiangya Hospital cohort using MeDIP-seq. DMRs enriched in PCa were selected using thresholds of BH-adjusted *P* < 0.05, logFC ≥ 1, and mean Reads Per Kilobase of transcript per Million mapped reads (RPKM) ≤ 0.5 in controls. To prioritize PCa-specific events, TCGA methylation data were analyzed using the limma package across multiple comparisons (PCa vs. AN, RCC, BCa, and peripheral blood leukocytes). Loci were defined as PCa-specific hypermethylated sites if they met BH-P ≤ 0.05 across all comparisons, with mean β ≥ 0.5 in PCa and low methylation in non-tumor tissues (β ≤ 0.1 in normal tissues and leukocytes, and ≤ 0.2 in RCC and BCa). Intersecting DMRs from MeDIP-seq and TCGA analyses were further validated in 67 tissue samples (19 PCa, 29 BPH, 19 BCa) using multiplex PCR-based targeted methylation sequencing. Candidate markers were defined as loci with < 10% methylation in controls, > 30% in cancer tissues, and BH-P ≤ 0.05.

### Methylation analysis by qPCR

To translate the tissue-derived biomarkers into noninvasive clinical assays, qPCR was employed to analyze the bisulfite-converted urine samples from both the training (*n* = 348) and prospective validation (*n* = 200) cohorts. qPCR validation primers and probes were designed based on differentially methylated sites identified through multiplex PCR-based targeted methylation sequencing (primer and probe sequences are provided in Table S5). For urine samples, 50–100 ng of genomic DNA was subjected to bisulfite conversion and subsequent purification using the EZ DNA Methylation-Lightning Kit (Zymo Research, catalog no. D5031), followed by qPCR analysis of the converted DNA. The relative methylation level of each target was quantified using the ΔCt method, calculated as: ΔCt = Ct_target gene_​ − Ct_reference gene_.

### Methylation model development for PCa detection

Based on the differentially methylated sites identified by targeted sequencing, qPCR primers and TaqMan probes were designed. To ensure translational feasibility, a screening subset of 38 urine samples (16 PCa and 22 BPH) was randomly partitioned from the training cohort (total *n* = 348) for initial CpG biomarker selection. Urine ΔCt values (Ct_target gene_​ − Ct_reference gene_) were analyzed using Least Absolute Shrinkage and Selection Operator (LASSO) regression and random forest algorithms within a 10-fold cross-validation framework, independently repeated 10 times. To prevent data leakage, feature selection and variable penalty tuning were strictly restricted to the internal training partition of each fold. Candidate markers were selected based on their high selection frequency and stable coefficients across all iterations.

Following this filtering, the three top-performing candidate genes were subjected to formal model construction in the complete training cohort (*n* = 348). For combinatorial diagnostic models, a 10-fold cross-validation approach partitioned the dataset to train and optimize logistic regression models. The optimal diagnostic threshold for each combination was determined by maximizing the Youden index. Finally, the best-performing two-gene combinatorial model (*ADD3_2* + *GSX2_2*) was blindly evaluated in the independent, prospective multicenter validation cohort (*n* = 200).

### Statistics

Differential DNA methylation analysis of the public TCGA and GEO datasets was performed using the limma R package by comparing β-values between groups, with *P*-values adjusted using the Benjamini–Hochberg method. For multiplex PCR–based targeted methylation sequencing, methylation ratios between groups were also compared using limma, and *P*-values were corrected with the same method. LASSO regression was applied to select candidate targets using the glmnet R package. Model performance was assessed using ROC and DCA, including evaluation of AUC, accuracy, sensitivity, specificity, and standardized net benefit (sNB). The sNB was calculated as follows: sNB^(opt−in)^(r) = TPR(r) − ($\frac{1-\rho }{\rho }$)($\frac{r}{1-r}$) FPR(r), where TPR is the true positive rate, FPR is the false positive rate, ρ represents the disease prevalence, and r denotes the clinical decision threshold probability. Additionally, the biopsy reduction rate was defined as: Biopsy Reduction Rate = (Standard Biopsy Indications—Model-Referred Biopsies)/Total Cohort Patients. ROC curves were generated with the pROC package, and DCA was performed using the rmda package. Heatmaps were generated using the ComplexHeatmap R package, and other visualizations were produced using ggplot2. All model construction, statistical analyses, and data visualization were conducted in R version 4.2.1.

## Supplementary Information

Supplementary Material 1.

## Data Availability

The datasets supporting the findings of this study are publicly available in the GEO and TCGA repositories under the following accession numbers: TCGA-PRAD (https://portal.gdc.cancer.gov/analysis_page?app=Downloads) and GSE40279 (https://www.ncbi.nlm.nih.gov/geo/query/acc.cgi?acc=GSE40279). These standardized public data can be freely downloaded from the UCSC Xena Browser (https://xena.ucsc.edu/). Sequencing, DNA methylation data have been deposited in the European Genome-Phenome Archive (https://www.ebi.ac.uk/ega/datasets) under accession EGAS50000001894. All clinical characteristic data are described within the main text. Due to confidentiality considerations, the raw sequencing data are available from the corresponding author, Dr. Long Wang, upon reasonable request (email: wanglong@csu.edu.cn).
