# Supplementary material for: Development and validation of a two-gene urine DNA methylation assay for noninvasive prostate cancer detection: a prospective multicenter study
Source: Mol Biomed. 2026 Aug 7;7:131. doi: 10.1186/s43556-026-00528-y (PMC13451169; doi:10.1186/s43556-026-00528-y)
Supplement: Supplementary file 1 [file 43556_2026_528_MOESM1_ESM.doc]

**Development and Validation of a Two-Gene Urine DNA Methylation Assay for Noninvasive Prostate Cancer Detection: A Prospective Multicenter Study**

Dingwen Liu^†1^,Lian Tang^†2^,Wei Xiong^†1^, Ran Xu^3^, Qing Zhou^4^,Genming Xu^2^,Zhen Li^5^, Wei Tang^2^, Jinru Feng^2^, Xiaowei Zeng^2^, Jinlei Liu^2^,Chao Li^1#^, Lei Chen^6#^ , Long Wang^1#^

^1^Department of Urology, The Third Xiangya Hospital, Central South University, Changsha, Hunan 410013, China.

^2^Yearth Biotechnology Co. Ltd., Changsha 410008, China.

^3^Department of Urology, The Second Xiangya Hospital of Central South University, Changsha, Hunan 410011, China.

^4^Department of Andrology, The First Hospital of Hunan University of Chinese Medicine, Changsha 410007, China.

^5^Department of Urology, Hunan Provincial People’s Hospital, The First Affiliated Hospital of Hunan Normal University, Changsha, Hunan, China.

^6^Department of Urology, Zhuzhou Central Hospital, Zhuzhou, Hunan 412007, China.

^#^Correspondence: Prof. Long Wang. Email: wanglong@csu.edu.cn.

^#^Correspondence: Prof. Lei Chen. Email: ChenL0412@126.com.

^#^Correspondence: Prof. Chao Li. Email: li4307chao@126.com.

†These authors contributed equally to this work.

**Supplemental Fig. S1. Discovery of DNA methylation markers to differentiate prostate cancer from BPH and bladder cancer tissues.**

**(a)Unsupervised hierarchical clustering of 3679 methylation markers among PCa (n = 484), adjacent normal tissues (n = 49), BCa (n = 418), normal bladder tissues (n = 21), RCC (n = 814), normal kidney tissues (n = 207) , and normal peripheral blood leukocytes (n = 656) in the TCGA and GEO cohorts. (b) Unsupervised hierarchical cluster**ing of 627 DMRs among PCa (n = 19), BPH (n = 29), and BCa tissues (n = 19) in the Third Xiangya Hospital cohort.

**DMRs, differential methylation regions;** PCa, prostate cancer; BCa, bladder cancer; BPH, Benign prostatic hyperplasia; **RCC, renal cell carcinoma;** TCGA, the Cancer Genome Atlas; GEO, Gene Expres sion Omnibus; RPKM, Reads Per Kilobase of transcript per Million mapped reads

**Supplementary Fig. S2. Distribution of DNA methylation β values for marker set I.** (a–n) Boxplots show the β-value distributions of 14 DNA methylation markers across normal bladder tissue (n = 21), bladder cancer (BCa; n = 418), normal kidney tissue (n = 207), renal cell carcinoma (RCC; n = 814), adjacent normal prostate tissue (n = 49), prostate cancer (PCa; n = 484), and white blood cells (WBC; n = 656), all from the TCGA and GEO cohorts. Center lines indicate medians, boxes indicate interquartile ranges, and points denote outliers. One-way ANOVA was performed for each marker (all P < 2.2 × 10^−16). PCa, prostate cancer; BCa, bladder cancer; RCC, renal cell carcinoma; WBC, white blood cells.

**Supplementary Fig. S3. Distribution of DNA methylation β values for marker set II.** (a–n) Boxplots show the β-value distributions of 14 DNA methylation markers across normal bladder tissue (n = 21), BCa (n = 418), normal kidney tissue (n = 207), RCC (n = 814), adjacent normal prostate tissue (n = 49), PCa (n = 484), and WBC (n = 656), all from the TCGA and GEO cohorts. Center lines indicate medians, boxes indicate interquartile ranges, and points denote outliers. One-way ANOVA was performed for each marker (all P < 2.2 × 10^−16). PCa, prostate cancer; BCa, bladder cancer; RCC, renal cell carcinoma; WBC, white blood cells.

**Supplementary Fig. S4. Distribution of DNA methylation β values for marker set III.** (a–n) Boxplots show the β-value distributions of 14 DNA methylation markers across normal bladder tissue (n = 21), BCa (n = 418), normal kidney tissue (n = 207), RCC (n = 814), adjacent normal prostate tissue (n = 49), PCa (n = 484), and WBC (n = 656), all from the TCGA and GEO cohorts. Center lines indicate medians, boxes indicate interquartile ranges, and points denote outliers. One-way ANOVA was performed for each marker (all P < 2.2 × 10^−16). PCa, prostate cancer; BCa, bladder cancer; RCC, renal cell carcinoma; WBC, white blood cells.

**Supplementary Fig. S5. RPKM distributions of DMR-associated transcripts, set I.** (a–k) Boxplots show the expression levels of the indicated transcripts in bladder cancer (BCa; red; n = 19), benign prostatic hyperplasia (BPH; green; n = 29), and prostate cancer (PCa; blue; n = 19) samples from the Third Xiangya Hospital cohort. Points represent individual samples; boxes indicate medians and interquartile ranges. RPKM, reads per kilobase of transcript per million mapped reads; DMR, differentially methylated region.

**Supplementary Fig. S6. RPKM distributions of DMR-associated transcripts, set II.** (a–k) Boxplots show the expression levels of the indicated transcripts in BCa (red; n = 19), BPH (green; n = 29), and PCa (blue; n = 19) samples from the Third Xiangya Hospital cohort. Points represent individual samples; boxes indicate medians and interquartile ranges. RPKM, reads per kilobase of transcript per million mapped reads; DMR, differentially methylated region; BCa, bladder cancer; BPH, benign prostatic hyperplasia; PCa, prostate cancer.

**Supplementary Fig. S7. RPKM distributions of DMR-associated transcripts, set III.** (a–j) Boxplots show the expression levels of the indicated transcripts in BCa (red; n = 19), BPH (green; n = 29), and PCa (blue; n = 19) samples from the Third Xiangya Hospital cohort. Points represent individual samples; boxes indicate medians and interquartile ranges. RPKM, reads per kilobase of transcript per million mapped reads; DMR, differentially methylated region; BCa, bladder cancer; BPH, benign prostatic hyperplasia; PCa, prostate cancer.

**Supplementary Fig. S8. RPKM distributions of DMR-associated transcripts, set IV. (a–j) Boxplots show the expression levels of the indicated transcripts in BCa (red; n = 19), BPH (green; n = 29), and PCa (blue; n = 19) samples from the Third Xiangya Hospital cohort. Points represent individual samples; boxes indicate medians and interquartile ranges. RPKM, reads per kilobase of transcript per million mapped reads; DMR, differentially methylated region; BCa, bladder cancer; BPH, benign prostatic hyperplasia; PCa, prostate cancer.**

**Supplemental Fig. S9 Net percentage reduction in biopsies calculated by the decision curve analysis (DCA).**

**The x‐axis details the range of accepted risks a clinician or patient may accept before deciding to biopsy. Panels show the percentage reduction in biopsies based upon the detection of varying levels of disease severity： a, the whole validation dataset; b, the subset of PSA grey zone (4-10 ng/ml) in the validation dataset; c, ≥GG2 vs. control group in the validation dataset; d, ≥GG3 vs. control group in the validation dataset.**

**DCA, decision curve analysis; PSA, prostate-specific antigen; GG, Gleason Grade Group**

**Supplemental Fig. S10 Correlation and Bland-Altman consistency analyses were conducted to evaluate the stability and reproducibility of ΔCt values at different time points. Correlation analysis for *ADD3_2* (Day 0 vs. Day 5: a; ΔCt: c) and *GSX2_2* detection (Day 0 vs. Day 5: e; ΔCt: g). Bland-Altman agreement analysis for *ADD3_2* (Day 0 vs. Day 5: b; ΔCt: d) and *GSX2_2* detection (Day 0 vs. Day 5: f; ΔCt: h).**

**ΔCt: Ct_target gene_ - Ct_reference gene_**

**Table S1. Subject baseline demographics and clinical characteristics in the Tissue cohort from the Third Xiangya Hospital.**

| Characteristics | case | control | |
| --- | --- | --- | --- |
|  | PCa(n=19) | BPH(n=29) | BCa(n=19) |
| Age |  | | |
| Median（IQR） | 70(62–76) | 72(61-77) | 60(56-73) |
| PSA |  | | |
| Median（IQR） | 21.29(12-63.42) | 5.03 (2.72–8.76) | 2.54(0.92-4.32) |
| Gleason |  | | |
| GG1 | 1(5%) |  |  |
| GG2 | 4(21%) |  |  |
| GG3 | 5(26%) |  |  |
| GG4 | 5(26%) |  |  |
| GG5 | 4(21%) |  |  |

PCa, prostate cancer; BPH, benign prostatic hyperplasia; BCa, bladder cancer

**Table S2 Genomic coordinates of differentially methylated regions (DMRs) and CpG site positions**

| DMR number | DMRs | | | Cg |
| --- | --- | --- | --- | --- |
|  | chr | start | stop |  |
| DMR 1 | chr1 | 48937201 | 48937500 | 48937404-48937405(SPATA6:cg01710865);  48937445-48937446(SPATA6:cg18267506); |
| DMR 2 | chr1 | 48937501 | 48937800 | 48937404-48937405(SPATA6:cg01710865);  48937445-48937446(SPATA6:cg18267506); |
| DMR 3 | chr1 | 116711101 | 116711400 | 116711075-116711076(.:cg27527108); |
| DMR 4 | chr2 | 29337901 | 29338200 | 29338113-29338114(CLIP4:cg23428985); |
| DMR 5 | chr3 | 25469401 | 25469700 | 25469720-25469721(RARB:cg02499249); |
| DMR 6 | chr3 | 134082901 | 134083200 | 134083080-134083081(AMOTL2:cg05304177);  134083158-134083159(AMOTL2:cg16371082); |
| DMR 7 | chr3 | 134083201 | 134083500 | 134083158-134083159(AMOTL2:cg16371082); |
| DMR 8 | chr3 | 193776001 | 193776300 | 193776240-193776241(LINC02028:cg08825929); |
| DMR 9 | chr4 | 54965701 | 54966000 | 54965863-54965864(GSX2:cg13506653); |
| DMR 10 | chr5 | 7850101 | 7850400 | 7850203-7850204(C5orf49:cg12539796); |
| DMR 11 | chr5 | 33891901 | 33892200 | 33892223-33892224(ADAMTS12:cg26573704); |
| DMR 12 | chr5 | 112629301 | 112629600 | 112629565-112629566(MCC:cg07249274); |
| DMR 13 | chr5 | 112629601 | 112629900 | 112629565-112629566(MCC:cg07249274); |
| DMR 14 | chr5 | 169064101 | 169064400 | 169064451-169064452(DOCK2:cg08862890); |
| DMR 15 | chr6 | 2841601 | 2841900 | 2841875-2841876(SERPINB1:cg07635623); |
| DMR 16 | chr6 | 79620301 | 79620600 | 79620363-79620364(AL450327.1:cg18132916); |
| DMR 17 | chr6 | 146136301 | 146136600 | 146136563-146136564(FBXO30:cg23095615); |
| DMR 18 | chr6 | 146136601 | 146136900 | 146136563-146136564(FBXO30:cg23095615); |
| DMR 19 | chr7 | 100464901 | 100465200 | 100465051-100465052(TRIP6:cg12507655); |
| DMR 20 | chr8 | 30243301 | 30243600 | 30243260-30243261(RBPMS:cg00447833); |
| DMR 21 | chr10 | 74020801 | 74021100 | 74020976-74020977(.:cg12799885); |
| DMR 22 | chr10 | 104433901 | 104434200 | 104434010-104434011(ARL3:cg06361405); |
| DMR 23 | chr10 | 111767101 | 111767400 | 111767379-111767380(ADD3:cg05098590); |
| DMR 24 | chr11 | 57250201 | 57250500 | 57250469-57250470(.:cg00264441); |
| DMR 25 | chr11 | 57250501 | 57250800 | 57250469-57250470(.:cg00264441); |
| DMR 26 | chr11 | 58940701 | 58941000 | 58940866-58940867(DTX4:cg05415131); |
| DMR 27 | chr11 | 61062901 | 61063200 | 61062910-61062911(VWCE:cg06282270); |
| DMR 28 | chr12 | 89744701 | 89745000 | 89744701-89744702(DUSP6:cg05769889); |
| DMR 29 | chr13 | 36919501 | 36919800 | 36919674-36919675(SPG20:cg20691205); |
| DMR 30 | chr15 | 59729401 | 59729700 | 59729553-59729554(FAM81A:cg25489866); |
| DMR 31 | chr16 | 68118601 | 68118900 | 68118754-68118755(NFATC3:cg00402172); |
| DMR 32 | chr16 | 88717501 | 88717800 | 88717456-88717457(CYBA:cg04879832);  88717464-88717465(CYBA:cg08843517); |
| DMR 33 | chr17 | 42386401 | 42386700 | 42386326-42386327(RUNDC3A:cg01635086);  42386419-42386420(RUNDC3A:cg03936721); |
| DMR 34 | chr18 | 59559901 | 59560200 | 59560289-59560290(RNF152:cg01037637); |
| DMR 35 | chr18 | 59560201 | 59560500 | 59560289-59560290(RNF152:cg01037637); |
| DMR 36 | chr19 | 51415801 | 51416100 | 51416098-51416099(KLK4:cg17355294); |
| DMR 37 | chr19 | 51416101 | 51416400 | 51416098-51416099(KLK4:cg17355294); |
| DMR 38 | chr19 | 51522601 | 51522900 | 51522561-51522562(KLK10:cg24512400);  51522588-51522589(KLK10:cg15910208);  51522894-51522895(KLK10:cg18437710); |
| DMR 39 | chr20 | 44097901 | 44098200 | 44098287-44098288(WFDC2:cg06385187); |
| DMR 40 | chr20 | 44098201 | 44098500 | 44098287-44098288(WFDC2:cg06385187);  44098387-44098388(WFDC2:cg23740882);  44098409-44098410(WFDC2:cg14151783); |
| DMR 41 | chr21 | 46143301 | 46143600 | 46143498-46143499(.:cg04285033); |
| DMR 42 | chr22 | 28839301 | 28839600 | 28839249-28839250(TTC28:cg04294888); |

**Table S3. List of Multiplex Bisulfite PCR Primers for Target Sequences**

| **Primer Name** | **Sequence** |
| --- | --- |
| SPA-bis-F1-P5 | AGATGTGTATAAGAGACAGYGGGTATTGGTTTAGGGA |
| SPA-bis-R1-P7 | GTGACTGGAGTTCAGACGTGTGCTCTTCCGATCTAACAAAATAAAACAAAACCRAA |
| SPA-bis-F2-P5 | AGATGTGTATAAGAGACAGAGAAGYGTTTTTTTATTTYGG |
| SPA-bis-R2-P7 | GTGACTGGAGTTCAGACGTGTGCTCTTCCGATCTATCTAAAAACRCRCAAATT |
| SPA-bis-F3-P5 | AGATGTGTATAAGAGACAGAATTTTTTAGTGAGAGAGAGTGTATG |
| SPA-bis-R3-P7 | GTGACTGGAGTTCAGACGTGTGCTCTTCCGATCTACCCCCAAACTTAAAAAAA |
| CLI-bis-F1-P5 | AGATGTGTATAAGAGACAGGGGTGTAGTACGGATTAGGTTT |
| CLI-bis-R1-P7 | GTGACTGGAGTTCAGACGTGTGCTCTTCCGATCTTCTTAAACTCCCCAAAATCC |
| CLI-bis-F2-P5 | AGATGTGTATAAGAGACAGGGTTTTAGTAGTTGGTGGGG |
| CLI-bis-R2-P7 | GTGACTGGAGTTCAGACGTGTGCTCTTCCGATCTATCGCAATATTACCTCCC |
| CLI-bis-F3-P5 | AGATGTGTATAAGAGACAGAGAYGTTGTTAGTYGGGGT |
| CLI-bis-R3-P7 | GTGACTGGAGTTCAGACGTGTGCTCTTCCGATCTCCTACRCTAACTCRCCTAACT |
| RARB-bis-F5-P5 | AGATGTGTATAAGAGACAGAGAGAAGTTGGTGTTTAAYGTGAGT |
| RARB-bis-R5-P7 | GTGACTGGAGTTCAGACGTGTGCTCTTCCGATCTTCACCTTAAAAACAATACAAAAAATAC |
| RARB-bis-F6-P5 | AGATGTGTATAAGAGACAGGTTAGATTAGTTGGGTTATTTGAA |
| RARB-bis-R6-P7 | GTGACTGGAGTTCAGACGTGTGCTCTTCCGATCTCACACAAAATAAAAAATTAAATTACC |
| RARB-bis-F7-P5 | AGATGTGTATAAGAGACAGGTTAAAGGGGGGATTAGAATT |
| RARB-bis-R7-P7 | GTGACTGGAGTTCAGACGTGTGCTCTTCCGATCTATACCCAAACAAACCCTACTC |
| AMO-bis-F1-P5 | AGATGTGTATAAGAGACAGGTTTTTGGGAGTTTGTTAGTGG |
| AMO-bis-R1-P7 | GTGACTGGAGTTCAGACGTGTGCTCTTCCGATCTATAACTCCAACRAAAAACCAAA |
| AMO-bis-F2-P5 | AGATGTGTATAAGAGACAGGGTGATTTGGATGTTTGGTATT |
| AMO-bis-R2-P7 | GTGACTGGAGTTCAGACGTGTGCTCTTCCGATCTCCCAAAACCRAACRAAAA |
| AMO-bis-F3-P5 | AGATGTGTATAAGAGACAGGAGGGTGAGTATAAAGGGAGT |
| AMO-bis-R3-P7 | GTGACTGGAGTTCAGACGTGTGCTCTTCCGATCTCRAAAACCAACRAAAAAA |
| LIN-bis-F1-P5 | AGATGTGTATAAGAGACAGAGGGTTTTGGAGAAGGTGG |
| LIN-bis-R1-P7 | GTGACTGGAGTTCAGACGTGTGCTCTTCCGATCTCRCACAAACCCRAATAAC |
| LIN-bis-F2-P5 | AGATGTGTATAAGAGACAGTGATTTTTGAGTAGTTGYGTT |
| LIN-bis-R2-P7 | GTGACTGGAGTTCAGACGTGTGCTCTTCCGATCTAAAATACCAAAAACCTAACCAAA |
| LIN-bis-F3-P5 | AGATGTGTATAAGAGACAGTGTTTGAGAGGTTTTGGG |
| LIN-bis-R3-P7 | GTGACTGGAGTTCAGACGTGTGCTCTTCCGATCTCAACCCCAACATCAACTC |
| GSX-bis-F1-P5 | AGATGTGTATAAGAGACAGGTTTTTTAGTTGGGAGGTTTTA |
| GSX-bis-R1-P7 | GTGACTGGAGTTCAGACGTGTGCTCTTCCGATCTCCCTATCCACAACTTTTAAACT |
| GSX-bis-F2-P5 | AGATGTGTATAAGAGACAGGYGTTTAGGGATTGGAYG |
| GSX-bis-R2-P7 | GTGACTGGAGTTCAGACGTGTGCTCTTCCGATCTCAATATTTCCAACTCCTCTATAATAA |
| GSX-bis-F3-P5 | AGATGTGTATAAGAGACAGAAGTTTTTATTTGGTAYGTTTTG |
| GSX-bis-R3-P7 | GTGACTGGAGTTCAGACGTGTGCTCTTCCGATCTACRCTTTACTAAAAAATCACAA |
| ADA-bis-F3-P5 | AGATGTGTATAAGAGACAGTAGTAGGAAGATGTAGGGGTGT |
| ADA-bis-R3-P7 | GTGACTGGAGTTCAGACGTGTGCTCTTCCGATCTCCTTTCTACTTAAAAAAACACAAAA |
| ADA-bis-F4-P5 | AGATGTGTATAAGAGACAGGGGAGTGATTTAGTTGATAGTGT |
| ADA-bis-R4-P7 | GTGACTGGAGTTCAGACGTGTGCTCTTCCGATCTCCRCRAAACATACRAAAA |
| ADA-bis-F5-P5 | AGATGTGTATAAGAGACAGTYGGTTAAATAGGGGAGA |
| ADA-bis-R5-P7 | GTGACTGGAGTTCAGACGTGTGCTCTTCCGATCTATTTTAAACTTATATTTAAACACRCC |
| MC-bis-F1-P5 | AGATGTGTATAAGAGACAGGAGGYGTTTTATTTAYGATAGT |
| MC-bis-R1-P7 | GTGACTGGAGTTCAGACGTGTGCTCTTCCGATCTTTACCRATCAAAAAAATAAAAAA |
| MC-bis-F2-P5 | AGATGTGTATAAGAGACAGGGAYGTATTGGATTATTTTATTT |
| MC-bis-R2-P7 | GTGACTGGAGTTCAGACGTGTGCTCTTCCGATCTAATCATTCATCTCAACCTAAAA |
| MC-bis-F3-P5 | AGATGTGTATAAGAGACAGTGGATTTTTGAGTTTTTGAGGT |
| MC-bis-R3-P7 | GTGACTGGAGTTCAGACGTGTGCTCTTCCGATCTTTCAAAACTAATCTCAAACACTCAA |
| SER-bis-F3-P5 | AGATGTGTATAAGAGACAGGGATTAGAGGTYGTTTGTTT |
| SER-bis-R3-P7 | GTGACTGGAGTTCAGACGTGTGCTCTTCCGATCTAAACTCAAACCCCTCAAA |
| FB-bis-F4-P5 | AGATGTGTATAAGAGACAGATAGGGTTGTATTAAAGAATGGATG |
| FB-bis-R4-P7 | GTGACTGGAGTTCAGACGTGTGCTCTTCCGATCTACTCCCAACACCCAAAAC |
| TRI-bis-F1-P5 | AGATGTGTATAAGAGACAGGTTGATTTAGGGAGAAATGGTAGT |
| TRI-bis-R1-P7 | GTGACTGGAGTTCAGACGTGTGCTCTTCCGATCTCCTCTTCCTCCCTAATTTTAAAA |
| TRI-bis-F2-P5 | AGATGTGTATAAGAGACAGAAAAAGTTTTTTTTTTTGGAGTT |
| TRI-bis-R2-P7 | GTGACTGGAGTTCAGACGTGTGCTCTTCCGATCTTCCAAACAACRATCTTAAAAC |
| TRI-bis-F3-P5 | AGATGTGTATAAGAGACAGAGTTTTTTAGGGGAGGGYG |
| TRI-bis-R3-P7 | GTGACTGGAGTTCAGACGTGTGCTCTTCCGATCTCTCTTCTATCTCACAAAAACTACCTT |
| RBP-bis-F1-P5 | AGATGTGTATAAGAGACAGTATAGTGTGGTTYGGGGAG |
| RBP-bis-R1-P7 | GTGACTGGAGTTCAGACGTGTGCTCTTCCGATCTAATCTCCRAAAAATCCTTAAAA |
| RBP-bis-F2-P5 | AGATGTGTATAAGAGACAGGAGGGTTAGGATTTAGGGT |
| RBP-bis-R2-P7 | GTGACTGGAGTTCAGACGTGTGCTCTTCCGATCTAAAAATATCTTTTAACRAAAACTTTC |
| RBP-bis-F3-P5 | AGATGTGTATAAGAGACAGTTAAAGATTTYGAGTTTGGG |
| RBP-bis-R3-P7 | GTGACTGGAGTTCAGACGTGTGCTCTTCCGATCTAAAAATACCCTCRATAAACATTTA |
| ADD-bis-F1-P5 | AGATGTGTATAAGAGACAGGTAGTTAGYGTGGGYGGT |
| ADD-bis-R1-P7 | GTGACTGGAGTTCAGACGTGTGCTCTTCCGATCTATCTCCCRCCCTAAATAAA |
| ADD-bis-F2-P5 | AGATGTGTATAAGAGACAGTTTTTATYGGGGTYGTTTTTT |
| ADD-bis-R2-P7 | GTGACTGGAGTTCAGACGTGTGCTCTTCCGATCTCTACCTAACRCCTTTTCAAC |
| ADD-bis-F3-P5 | AGATGTGTATAAGAGACAGYGGTTGTYGGGTTTGTAG |
| ADD-bis-R3-P7 | GTGACTGGAGTTCAGACGTGTGCTCTTCCGATCTCCTCRAAAATCCTAATCC |
| DTX-bis-F1-P5 | AGATGTGTATAAGAGACAGAGTTGGGTTTTGGGTGTG |
| DTX-bis-R1-P7 | GTGACTGGAGTTCAGACGTGTGCTCTTCCGATCTTAATCTCTCCTCCCAAAAAACC |
| DTX-bis-F2-P5 | AGATGTGTATAAGAGACAGGGGTTGGGGTATTTTGGA |
| DTX-bis-R2-P7 | GTGACTGGAGTTCAGACGTGTGCTCTTCCGATCTCTCTCCATCTACTCCRACCTATT |
| VWC-bis-F1-P5 | AGATGTGTATAAGAGACAGGGGGTAGGTTTAGAATTGTT |
| VWC-bis-R1-P7 | GTGACTGGAGTTCAGACGTGTGCTCTTCCGATCTCCRAACATAACCCCTATC |
| VWC-bis-F2-P5 | AGATGTGTATAAGAGACAGTAGTTTTGTTTTYGAAGTTTGTTT |
| VWC-bis-R2-P7 | GTGACTGGAGTTCAGACGTGTGCTCTTCCGATCTCCCCTACCCCRATTTAAA |
| VWC-bis-F3-P5 | AGATGTGTATAAGAGACAGTTAGGGGTTTTTGATTTTTGT |
| VWC-bis-R3-P7 | GTGACTGGAGTTCAGACGTGTGCTCTTCCGATCTAAACRAAACACACAAAAACAA |
| RNF-bis-F1-P5 | AGATGTGTATAAGAGACAGTTTTTTTTTTAGGGTTTATTGTTG |
| RNF-bis-R1-P7 | GTGACTGGAGTTCAGACGTGTGCTCTTCCGATCTATCTCTACAAAATAAATAACCACCTC |
| RNF-bis-F2-P5 | AGATGTGTATAAGAGACAGTAATTAGGAGTYGTGAYGTT |
| RNF-bis-R2-P7 | GTGACTGGAGTTCAGACGTGTGCTCTTCCGATCTCCCTCCTAAACAAACRAA |
| RNF-bis-F3-P5 | AGATGTGTATAAGAGACAGTGTTATTAYGTAGAAGGGGTGAA |
| RNF-bis-R3-P7 | GTGACTGGAGTTCAGACGTGTGCTCTTCCGATCTATAACCCAAAAACACCCAAAC |
| WFD-bis-F1-P5 | AGATGTGTATAAGAGACAGTTTAGGAGGGAGGTTAGGAG |
| WFD-bis-R1-P7 | GTGACTGGAGTTCAGACGTGTGCTCTTCCGATCTAAATCAAATATCACTCTTAACCCC |
| WFD-bis-F2-P5 | AGATGTGTATAAGAGACAGATTGTGAAATGATTTTATGTGG |
| WFD-bis-R2-P7 | GTGACTGGAGTTCAGACGTGTGCTCTTCCGATCTCRATACTCAAATACRAAAATTTAA |
| TTC-bis-F1-P5 | AGATGTGTATAAGAGACAGGGTTTGATTGTTTGTTTAATTTT |
| TTC-bis-R1-P7 | GTGACTGGAGTTCAGACGTGTGCTCTTCCGATCTTCCCTCAAAAAAAAAACTAAA |
| TTC-bis-F2-P5 | AGATGTGTATAAGAGACAGGGTTTTTGTAGTTAGTTTGGAGA |
| TTC-bis-R2-P7 | GTGACTGGAGTTCAGACGTGTGCTCTTCCGATCTAAAATCCCAACAACCACC |
| SPART-bis-F1-P5 | AGATGTGTATAAGAGACAGGGTTGGGTAGTTTTTTTTTG |
| SPART-bis-R1-P7 | GTGACTGGAGTTCAGACGTGTGCTCTTCCGATCTTCAATCCCTAAAAAACATAAAAA |
| SPART-bis-F2-P5 | AGATGTGTATAAGAGACAGATGTTTTTTAGGGATTGAGTTG |
| SPART-bis-R2-P7 | GTGACTGGAGTTCAGACGTGTGCTCTTCCGATCTAAAACTAAATCAACCCTAAAATCAC |
| SPART-bis-F3-P5 | AGATGTGTATAAGAGACAGAGTTTTTTATTTTTTAATTGTAAGGA |
| SPART-bis-R3-P7 | GTGACTGGAGTTCAGACGTGTGCTCTTCCGATCTCCATTATTTCAAAAACTATAACAAC |
| FAM81-bis-F1-P5 | AGATGTGTATAAGAGACAGGTYGGAGAGAGAATTTTAAAAT |
| FAM81-bis-R1-P7 | GTGACTGGAGTTCAGACGTGTGCTCTTCCGATCTAAAACTCCTATCTATCTCCCC |
| FAM81-bis-F2-P5 | AGATGTGTATAAGAGACAGTTTTGTGTTGGGTAAGTATTAGTAT |
| FAM81-bis-R2-P7 | GTGACTGGAGTTCAGACGTGTGCTCTTCCGATCTAAACCCATTCACTCATAATTATACT |
| FAM81-bis-F3-P5 | AGATGTGTATAAGAGACAGTTTGATGAGAATTTAAAATYGAGA |
| FAM81-bis-R3-P7 | GTGACTGGAGTTCAGACGTGTGCTCTTCCGATCTAAAAAAAAACCCCRAACAC |
| FAM81-bis-F4-P5 | AGATGTGTATAAGAGACAGGTGTTYGGGGTTTTTTTTT |
| FAM81-bis-R4-P7 | GTGACTGGAGTTCAGACGTGTGCTCTTCCGATCTATCATTTACTAAATCRCCTTAACAC |
| NFAT-bis-F1-P5 | AGATGTGTATAAGAGACAGTTGTTTATTGGGGTTYGTTG |
| NFAT-bis-R1-P7 | GTGACTGGAGTTCAGACGTGTGCTCTTCCGATCTATAACTACRCCTTTATCCCCACT |
| NFAT-bis-F2-P5 | AGATGTGTATAAGAGACAGGGTTYGTTAGTGGGGATAAA |
| NFAT-bis-R2-P7 | GTGACTGGAGTTCAGACGTGTGCTCTTCCGATCTAAAACCRAAAAAAACAAATT |
| NFAT-bis-F3-P5 | AGATGTGTATAAGAGACAGGTAATTTGTTTTTTTCGGTTT |
| NFAT-bis-R3-P7 | GTGACTGGAGTTCAGACGTGTGCTCTTCCGATCTCCCACCTCAACTTTAATAACTAA |
| CYBA-bis-F1-P5 | AGATGTGTATAAGAGACAGGTTGGGGTTTTGGGATAT |
| CYBA-bis-R1-P7 | GTGACTGGAGTTCAGACGTGTGCTCTTCCGATCTACCATATAAACCAACRAACAA |
| CYBA-bis-F2-P5 | AGATGTGTATAAGAGACAGGGAYGTTAGYGTTTGTTYGT |
| CYBA-bis-R2-P7 | GTGACTGGAGTTCAGACGTGTGCTCTTCCGATCTCAATACRCRCCTAACAATATCC |
| CYBA-bis-F3-P5 | AGATGTGTATAAGAGACAGTTAGGYGYGTATTGTYGT |
| CYBA-bis-R3-P7 | GTGACTGGAGTTCAGACGTGTGCTCTTCCGATCTCAACCCTACACCCTACAAAT |
| CYBA-bis-F4-P5 | AGATGTGTATAAGAGACAGTATTTGTAGGGTGTAGGGTTGTT |
| CYBA-bis-R4-P7 | GTGACTGGAGTTCAGACGTGTGCTCTTCCGATCTACTCCCCRCCTAAACTAA |
| CYBA-bis-F5-P5 | AGATGTGTATAAGAGACAGAGTTTAGGYGGGGAGTTTT |
| CYBA-bis-R5-P7 | GTGACTGGAGTTCAGACGTGTGCTCTTCCGATCTACTATTAATAAACRCCTAATTAACAC |
| RUND-bis-F1-P5 | AGATGTGTATAAGAGACAGGATAGAGGGTTTAGTYGTGAT |
| RUND-bis-R1-P7 | GTGACTGGAGTTCAGACGTGTGCTCTTCCGATCTTTTCTTAAAAAACAACCCCA |
| RUND-bis-F2-P5 | AGATGTGTATAAGAGACAGATTATGGTTTTGGGGTTGTTTT |
| RUND-bis-R2-P7 | GTGACTGGAGTTCAGACGTGTGCTCTTCCGATCTCCCACTAACRATACCCACCT |
| RUND-bis-F3-P5 | AGATGTGTATAAGAGACAGTTGTGGAGYGTAAGAATTTGAT |
| RUND-bis-R3-P7 | GTGACTGGAGTTCAGACGTGTGCTCTTCCGATCTACAACRCTCAATCCRAATTT |
| RUND-bis-F4-P5 | AGATGTGTATAAGAGACAGGATTGYGGAAATTYGGAT |
| RUND-bis-R4-P7 | GTGACTGGAGTTCAGACGTGTGCTCTTCCGATCTACCAAACTACAACCTCCCTCTAAA |
| C5orf-bis-F1-P5 | AGATGTGTATAAGAGACAGGTAGGAGGGTAGAGGGTTTT |
| C5orf-bis-R1-P7 | GTGACTGGAGTTCAGACGTGTGCTCTTCCGATCTTCCCTCTAAAATCTATCTACRAC |
| C5orf-bis-F2-P5 | AGATGTGTATAAGAGACAGTTGTTAGTTTGGGTTTGTYG |
| C5orf-bis-R2-P7 | GTGACTGGAGTTCAGACGTGTGCTCTTCCGATCTAAACRCTCTTTCTACCCA |
| C5orf-bis-F3-P5 | AGATGTGTATAAGAGACAGATTTTGGGTAGAAAGAGYG |
| C5orf-bis-R3-P7 | GTGACTGGAGTTCAGACGTGTGCTCTTCCGATCTCAAATAAAACCCRATACTCTTC |
| C5orf-bis-F4-P5 | AGATGTGTATAAGAGACAGAAGAGTATCGGGTTTTATTTGA |
| C5orf-bis-R4-P7 | GTGACTGGAGTTCAGACGTGTGCTCTTCCGATCTCAACTAAACCCCTAAAACRA |
| C5orf-bis-F5-P5 | AGATGTGTATAAGAGACAGTTGATYGTAGTTAAGATATTTATTYG |
| C5orf-bis-R5-P7 | GTGACTGGAGTTCAGACGTGTGCTCTTCCGATCTCCCCAAAAAATACAACTCA |
| KLK4-bis-F1-P5 | AGATGTGTATAAGAGACAGTGGTTTTAGTTAGAGGAGGGTA |
| KLK4-bis-R1-P7 | GTGACTGGAGTTCAGACGTGTGCTCTTCCGATCTACCCAAACTCCTTAATCCTAA |
| KLK4-bis-F2-P5 | AGATGTGTATAAGAGACAGTTTTAYGGATTTTGTGGAG |
| KLK4-bis-R2-P7 | GTGACTGGAGTTCAGACGTGTGCTCTTCCGATCTCTCCCCTAATAAATCCCC |
| KLK4-bis-F3-P5 | AGATGTGTATAAGAGACAGGGATTTATTAGGGGAGAGGTG |
| KLK4-bis-R3-P7 | GTGACTGGAGTTCAGACGTGTGCTCTTCCGATCTCCTACRAACACTATCCCTCAC |
| KLK4-bis-F4-P5 | AGATGTGTATAAGAGACAGGTGAGATTATTTAAGGAGTGGG |
| KLK4-bis-R4-P7 | GTGACTGGAGTTCAGACGTGTGCTCTTCCGATCTAAAACACAAAAACATTCCTACC |
| KLK10-bis-F1-P5 | AGATGTGTATAAGAGACAGAGTTGYGTTATTAGTAGYGGTAG |
| KLK10-bis-R1-P7 | GTGACTGGAGTTCAGACGTGTGCTCTTCCGATCTCTACACACACCCCAACAAAT |
| KLK10-bis-F2-P5 | AGATGTGTATAAGAGACAGTTTGTTGGGGTGTGTGTAG |
| KLK10-bis-R2-P7 | GTGACTGGAGTTCAGACGTGTGCTCTTCCGATCTCRTCTACCAACCCCTAAAATTA |
| KLK10-bis-F3-P5 | AGATGTGTATAAGAGACAGTAGGGGTTGGTAGAYGGG |
| KLK10-bis-R3-P7 | GTGACTGGAGTTCAGACGTGTGCTCTTCCGATCTCACTCRAACACAAAAAAAAC |
| KLK10-bis-F4-P5 | AGATGTGTATAAGAGACAGTGGAATAGYGGTAATGGGTATAATTA |
| KLK10-bis-R4-P7 | GTGACTGGAGTTCAGACGTGTGCTCTTCCGATCTAAAAAAAAACCTCRAACRC |
| KLK10-bis-F5-P5 | AGATGTGTATAAGAGACAGGYGTTTTTYGYGTTTTAG |
| KLK10-bis-R5-P7 | GTGACTGGAGTTCAGACGTGTGCTCTTCCGATCTCCCACTAACTCCRAACCT |
| KLK10-bis-F6-P5 | AGATGTGTATAAGAGACAGTTTTTTAATTTTATAGYGGGGG |
| KLK10-bis-R6-P7 | GTGACTGGAGTTCAGACGTGTGCTCTTCCGATCTTACACCCCACCCRAATTA |

**Table S4 Methylation levels of candidate prostate cancer–specific biomarkers identified by targeted bisulfite sequencing.**

| **Name** | **Mean.BPH.BCa** | **Mean.PCA** | **logFC** | **adj.P.Val** |
| --- | --- | --- | --- | --- |
| **ADA_5_33892402** | 2.65 | 35.64 | 3.16 | <0.001 |
| **ADA_5_33892429** | 2.77 | 30.88 | 2.85 | <0.001 |
| **ADD_1_111767228** | 0.83 | 30.51 | 4.20 | <0.001 |
| **ADD_1_111767249** | 1.82 | 42.43 | 3.93 | <0.001 |
| **ADD_2_111767345** | 0.65 | 35.98 | 4.58 | <0.001 |
| **ADD_2_111767350** | 0.62 | 36.27 | 4.65 | <0.001 |
| **ADD_3_111767479** | 0.99 | 39.22 | 4.42 | <0.001 |
| **ADD_3_111767481** | 0.72 | 40.06 | 4.90 | <0.001 |
| **ADD_3_111767493** | 1.63 | 32.93 | 3.48 | <0.001 |
| **ADD_3_111767530** | 0.93 | 33.85 | 4.93 | <0.001 |
| **AMO_1_134083159** | 1.00 | 31.42 | 3.98 | <0.001 |
| **C5orf_1_7849974** | 1.89 | 42.62 | 3.84 | <0.001 |
| **C5orf_2_7850036** | 1.72 | 42.12 | 4.43 | <0.001 |
| **C5orf_2_7850070** | 1.95 | 38.67 | 3.72 | <0.001 |
| **C5orf_5_7850244** | 2.16 | 37.18 | 3.37 | <0.001 |
| **CYBA_3_88717489** | 1.35 | 32.28 | 4.20 | <0.001 |
| **CYBA_4_88717571** | 0.86 | 38.28 | 4.78 | <0.001 |
| **CYBA_4_88717602** | 1.76 | 46.07 | 4.87 | <0.001 |
| **DTX_1_58940863** | 1.75 | 36.90 | 3.78 | <0.001 |
| **DTX_2_58940911** | 2.65 | 35.45 | 3.27 | <0.001 |
| **DTX_2_58940922** | 2.75 | 35.98 | 3.34 | <0.001 |
| **GSX_2_54965883** | 3.66 | 31.25 | 2.75 | <0.001 |
| **GSX_3_54966019** | 3.86 | 30.04 | 2.68 | <0.001 |
| **GSX_3_54966058** | 3.86 | 32.49 | 2.68 | <0.001 |
| **KLK4_3_51416160** | 2.15 | 32.86 | 3.73 | <0.001 |
| **LIN_2_193776257** | 1.04 | 32.80 | 3.88 | <0.001 |
| **LIN_3_193776078** | 2.48 | 35.69 | 3.17 | <0.001 |
| **LIN_3_193776093** | 1.53 | 34.95 | 3.74 | <0.001 |
| **LIN_3_193776121** | 1.13 | 33.11 | 3.90 | <0.001 |
| **MC_3_112629505** | 1.78 | 43.13 | 4.80 | <0.001 |
| **RBP_3_30243234** | 1.82 | 36.09 | 3.57 | <0.001 |
| **RNF_2_59560286** | 1.84 | 50.62 | 4.61 | <0.001 |
| **RNF_2_59560290** | 1.96 | 50.85 | 4.88 | <0.001 |
| **RNF_2_59560301** | 1.18 | 48.10 | 5.08 | <0.001 |
| **RNF_2_59560306** | 1.55 | 48.57 | 5.39 | <0.001 |
| **SPA_1_48937501** | 2.46 | 36.82 | 3.10 | <0.001 |
| **SPA_2_48937405** | 2.32 | 32.37 | 3.39 | <0.001 |
| **TRI_2_100465046** | 2.00 | 47.12 | 4.11 | <0.001 |
| **TRI_2_100465078** | 2.07 | 46.71 | 4.03 | <0.001 |
| **TRI_3_100465214** | 1.37 | 37.35 | 4.00 | <0.001 |

**Table S5. Sequences of qPCR Validation Primers and Probes for Targeted Methylation Sites.**

| **Primer Name** | **Sequences and Modifications** |
| --- | --- |
| LINC02028-Q-F1 | GTGATTTTTGAGTAGTTGCGTTATC |
| LINC02028-Q-R1 | ACATCTCTCCCCACCGACAAC |
| LINC02028-Q-P1 | FAM-ACTCGAATTTCCCCCGCCGCGAAACT-MGB |
| LINC02028-Q-F2 | GGTGGGGAGAGATGTCGC |
| LINC02028-Q-R2 | CCCCAACATCAACTCCGATTC |
| LINC02028-Q-P2 | FAM-ACAAACGTTCAACCCCTTCGAAAAT-MGB |
| LINC02028-Q-F3 | GAGAGGTTTTGGGAGGTCGTC |
| LINC02028-Q-R3 | CCCAACATCAACTCCGATTC |
| LINC02028-Q-P3 | FAM-ACAAACGTTCAACCCCTTCGAAA-MGB |
| KLK4-Q-F3 | GGATTTATTAGGGGAGAGGTG |
| KLK4-Q-R3 | CCTACGAACACTATCCCTCAC |
| KLK4-Q-P3 | FAM-CGCCGACTCCGCGATCTAC-MGB |
| ADAMTS12-Q-F5 | ATAGGGGAGATTCGGGAGAGTC |
| ADAMTS12-Q-R5 | TTTAAACTTATATTTAAACACGCCG |
| ADAMTS12-Q-P5 | FAM-CCTCTCACTCGCAACTTCCTCCAATA-MGB |
| SPATA6-Q-F1 | ATTTTTAGGGAGCGTCGATTTTAC |
| SPATA6-Q-R1 | AACGCGCAAATTAAAAATCG |
| SPATA6-Q-P1 | FAM-CAAAAATCAAACCTCTACGTAACCG-MGB |
| RNF152-Q-F2 | GAGGGGGGAGGTGGTTATTTATT |
| RNF152-Q-R2 | CTCCTAAACAAACGAAACTAAAACG |
| RNF152-Q-P2 | FAM-AACGACCGAAAAAAAAACGTCACGACT-MGB |
| C5orf49-Q-F2 | TTTTGTTAGTTTGGGTTTGTCGT |
| C5orf49-Q-R2 | AAACAACAAAACGCTCTTTCTACC |
| C5orf49-Q-P2 | FAM-CAACGAACTAAACGCGACT-MGB |
| ADD3-Q-F1 | AGTAGTTAGCGTGGGCGGTC |
| ADD3-Q-R1 | AAAAACGCCTCGAAAATATCTC |
| ADD3-Q-P1 | FAM-ACTCCCGAAACTAAACCGCCGCTT-MGB |
| ADD3-Q-F2 | TTTTTTGGTTTTTCGGGGAC |
| ADD3-Q-R2 | CCCGACAACCGAAAAAACCT |
| ADD3-Q-P2 | FAM-CGACTACCTAACGCCTTTTCA-MGB |
| GSX2-Q-F2 | GAGTTGCGTTTAGGGATTGGAC |
| GSX2-Q-R2 | CTCTATAATAAAAATAACTACGACGCG |
| GSX2-Q-P2 | FAM-AAACAAAATTATACGAACGACGAACT-MGB |
| GSX2-Q-F3 | AAGTTTTTATTTGGTACGTTTTGC |
| GSX2-Q-R3 | CGCTTTACTAAAAAATCACAACG |
| GSX2-Q-P3 | FAM-CTCTCGCTACCCTAACCGCAA-MGB |
| CYBA-Q-F1 | ATTGTTAGGCGCGTATTGTCG |
| CYBA-Q-R1 | CAACCCTACACCCTACAAATACG |
| CYBA-Q-P1 | FAM-CCAACCGCGATCACCTA-MGB |
| CYBA-Q-F2 | TCGGACGTTAGCGTTTGTTC |
| CYBA-Q-R2 | ACGAAATTCGACCGAAAACG |
| CYBA-Q-P2 | FAM-ACGACAATACGCGCCTAAC-MGB |
| CYBA-Q-F3 | TTGTAGGGTGTAGGGTTGTTTCG |
| CYBA-Q-R3 | CCGCCTAAACTAAAACTACCGAA |
| CYBA-Q-P3 | FAM-AAAAAACGAAAACAAAAAACTCC-MGB |
| AMOTL2-Q-F1 | TTTGGGAGTTTGTTAGTGGGTG |
| AMOTL2-Q-R1 | AACCAAAAACAACTCGACGCTC |
| AMOTL2-Q-P1 | FAM-ACGAAAACGCCTCCCGACCGA-MGB |
| AMOTL2-Q-F2 | GGATGTTTGGTATTTTAGAGTCGC |
| AMOTL2-Q-R2 | CCTCCTCCCAAAACCGAAC |
| AMOTL2-Q-P2 | FAM-AACGCCAAACAACGCGCACGA-MGB |
| DTX4-Q-F1 | GGGGGAGGAAGAAGGGATTT |
| DTX4-Q-R1 | CAACCCTAATCTCTCCTCCCA |
| DTX4-Q-P1 | FAM-CCGACGCTTCCCTTACCCGACT-MGB |
| DTX4-Q-F2 | ATAGTCGGGTAAGGGAAGCGTC |
| DTX4-Q-R2 | CATCTACTCCGACCTATTACTTCCG |
| DTX4-Q-P2 | FAM-AAACATTCGCCAATACCGC-MGB |
| MCC-Q-F2 | GGTGAGGGGGACGTATTGGAT |
| MCC-Q-R2 | AACCACCTTACCTAAACGTAACCA |
| MCC-Q-P2 | FAM-CGTAACCAAACGCATCGCACG-MGB |
| RBPMS-Q-F3 | TTTTAGTCGAGGTTTTTTGGATTA |
| RBPMS-Q-R3 | TTACTCTACTCCCCTCAACTACACG |
| RBPMS-Q-P3 | FAM-CACCGCCCAAACTCGAAATC-MGB |
| TRIP6-Q-F2 | TTATCGGAGGCGTGGTCGTT |
| TRIP6-Q-R2 | TAAAACCCCGAACCTCTAACC |
| TRIP6-Q-P2 | FAM-TTCCGTCCCGCACCTCGTTTA-MGB |
| TRIP6-Q-F3 | CGGAGTTCGTTAGAGTTTTTTAGG |
| TRIP6-Q-R3 | CCTACTCCGTAAACCGATAATAACC |
| TRIP6-Q-P3 | FAM-TACCGCGAAAAATCGCCCTC-MGB |
| mACTB-F(housekeeping gene) | GGTGTTTAAGATAGTGTTGTGG |
| mACTB-R(housekeeping gene) | CTACTTAATACACACTCCAAAACC |
| mACTB-P(housekeeping gene) | VIC-CTTTACACCAACCTCATAACCTTATC-MGB |

**Table S6 Diagnostic performance of 23 pairs of primers and probes in the small-sample training cohort.**

| **Lable** | **Specificity(%)** | **Sensitivity(%)** | **Accuracy(%)** | **Pvale** |
| --- | --- | --- | --- | --- |
| ***ADD3_2*** | 100.00 | 93.75 | 97.37 | <0.001 |
| ***ADD3_1*** | 100.00 | 87.50 | 94.74 | <0.001 |
| ***CYBA_1*** | 90.91 | 100.00 | 94.74 | <0.001 |
| ***CYBA_3*** | 95.45 | 87.50 | 92.11 | <0.001 |
| ***GSX2_2*** | 95.45 | 81.25 | 89.47 | <0.001 |
| ***CYBA_2*** | 100.00 | 68.75 | 86.84 | <0.001 |
| ***FBXO30_1*** | 95.45 | 75.00 | 86.84 | <0.001 |
| ***LINC02028_1*** | 86.36 | 87.50 | 86.84 | <0.001 |
| ***AMOTL2_1*** | 95.45 | 68.75 | 84.21 | 0.003 |
| ***LINC02028_3*** | 77.27 | 87.50 | 81.58 | <0.001 |
| ***TRIP6_2*** | 81.82 | 81.25 | 81.58 | <0.001 |
| ***DTX4_2*** | 95.45 | 56.25 | 78.95 | 0.028 |
| ***GSX2_3*** | 81.82 | 75.00 | 78.95 | 0.003 |
| ***LINC02028_2*** | 77.27 | 81.25 | 78.95 | <0.001 |
| ***KLK4_3*** | 95.45 | 56.25 | 78.95 | 0.002 |
| ***SPATA6_1*** | 100.00 | 50.00 | 78.95 | 0.036 |
| ***TRIP6_3*** | 72.73 | 87.50 | 78.95 | <0.001 |
| ***C5orf49_2*** | 86.36 | 62.50 | 76.32 | 0.013 |
| ***ADAMTS12_5*** | 90.91 | 50.00 | 73.68 | 0.098 |
| ***RNF152_2*** | 100.00 | 37.50 | 73.68 | 0.21 |
| ***AMOTL2_2*** | 54.55 | 87.50 | 68.42 | 0.013 |
| ***MCC_2*** | 86.36 | 43.75 | 68.42 | 0.142 |
| ***DTX4_1*** | 77.27 | 50.00 | 65.79 | 0.515 |

**Table S7 . The clinical performance of *ADD3_2, GSX2_2, CYBA_1*, and their combinations at different PSA levels in the training cohort.**

| **lable** | **specificity(%)** | **sensitivity(%)** | **Npv(%)** | **Ppv(%)** | **accuracy(%)** |
| --- | --- | --- | --- | --- | --- |
| ***ADD3_2, GSX2_2*** | 90.50  (86.78- 93.8) | 72.64  (64.15- 80.19) | 88.38  (85.04- 91.43) | 76.92  (70.3-84.15) | 85.06  (81.32-88.51) |
| ***ADD3_2,GSX2_2,CYBA_1*** | 85.95  (81.4- 90.08) | 76.42  (67.92- 83.96) | 89.27  (85.95- 92.48) | 70.43  (64- 77.27) | 83.05  (79.02- 86.78) |
| ***ADD3_2*** | 84.71  (80.17- 88.84) | 77.36  (68.87- 85.85) | 89.58  (86.16- 93.06) | 68.89  (62.5- 75.89) | 82.47  (78.45- 86.49) |
| ***ADD3_2, CYBA_1*** | 80.99  (76.03- 85.95) | 83.02  (75.47- 89.62) | 91.63  (88.34- 94.84) | 65.65  (60- 72.22) | 81.61  (77.59- 85.63) |
| ***CYBA_1*** | 88.43  (84.71- 92.15) | 66.98  (57.55- 75.47) | 85.95  (82.69- 89.36) | 71.72  (64.65- 79.17) | 81.9  (77.87- 85.63) |
| ***GSX2_2,CYBA_1*** | 80.99  (75.62- 85.95) | 74.53  (66.04- 82.08) | 87.91  (84.39- 91.24) | 63.28  (56.69- 70.09) | 79.02  (74.43- 83.05) |
| ***GSX2_2*** | 85.95  (81.4- 90.08) | 61.32  (51.89- 70.75) | 83.47  (80.16- 87.2) | 65.69  (58.26- 73.5) | 78.45  (74.43- 82.47) |

**Table S8 . The clinical performance of diagnostic model (*ADD3_2, GSX2_2*) in different PSA levels in the Training cohort.**

|  |  | **All** | | **PSA<4** | | **4≤PSA≤10** | | **PSA>10** | |
| --- | --- | --- | --- | --- | --- | --- | --- | --- | --- |
|  |  | **Case** | **Control** | **Case** | **Control** | **Case** | **Control** | **Case** | **Control** |
| **Predicted positive** | | 77 | 23 | 2 | 10 | 15 | 5 | 60 | 8 |
| **Predicted negative** | | 29 | 219 | 2 | 110 | 7 | 59 | 20 | 50 |
| **sensitivity（%）** | | 72.64  (64.15-80.19) | | 50  (0- 100) | | 68.18  (45.45- 86.36) | | 75  (65- 83.75) | |
| **specificity（%）** | | 90.5  (86.78- 93.8) | | 91.67  (86.65- 95.83) | | 92.19  (85.94- 98.44) | | 86.21  (75.86- 94.83) | |
| **PPV（%）** | | 76.92  (70.3- 84.15) | | 16.67  (0- 36.36) | | 75  (59.09- 93.33) | | 88.37  (81.43- 94.92) | |
| **NPV（%）** | | 88.38  (85.04- 91.43) | | 98.21  (96.46- 100) | | 89.39  (83.33- 95.24) | | 71.62  (63.63- 79.69) | |
| **accuracy（%）** | | 85.06  (81.32-88.51) | | 90.32  (84.68- 95.16) | | 86.05  (79.07- 93.02) | | 79.71  (72.46- 86.23) | |
| **AUC** | | 0.86  (0.82- 0.91) | | 0.71  (0.27- 1) | | 0.83  (0.714- 0.94) | | 0.85  (0.79- 0.92) | |

**Table S9 . The clinical performance of ADD3_2, GSX2_2, CYBA_1, and their combinations at different PSA levels in the validation cohort.**

| **lable** | **specificity(%)** | **sensitivity(%)** | **Npv(%)** | **Ppv(%)** | **accuracy(%)** |
| --- | --- | --- | --- | --- | --- |
| ***ADD3_2, GSX2_2*** | 90.71  (85.71- 95) | 76.67  (66.67- 86.67) | 90.14  (86.01- 94.12) | 78.12  (69.22- 87.27) | 86.50  (82.00- 91.00) |
| ***ADD3_2,GSX2_2,CYBA_1*** | 87.14  (81.43- 92.14) | 81.67  (71.67- 91.67) | 91.91  (87.59- 95.97) | 73.44  (64.7- 82.35) | 85.50  (80.50- 90.00) |
| ***GSX2_2,CYBA_1*** | 84.29  (77.86- 90) | 76.67  (66.67- 86.67) | 89.55  (85.12- 93.75) | 67.69  (59.21- 77.05) | 82.00  (76.50- 87.00) |
| ***ADD3_2*** | 85.00  (78.57- 90.71) | 78.33  (66.67- 88.33) | 90.30  (85.71- 94.53) | 69.33  (60.94- 78.33) | 83.00  (77.50- 88.00) |
| ***ADD3_2, CYBA_1*** | 79.29  (72.14- 85.73) | 83.33  (73.33- 93.33) | 91.87  (87.29- 96.23) | 63.41  (55.95- 72.06) | 80.50  (75.00- 86.00) |
| ***GSX2_2*** | 87.14  (81.43- 92.14) | 66.67  (55- 78.33) | 85.96  (81.56- 90.25) | 69.09  (58.93- 78.85) | 81.00  (75.49- 86.00) |
| ***CYBA_1*** | 84.29  (77.86- 90) | 73.33  (61.67- 83.33) | 87.97  (83.67- 92.48) | 66.67  (57.75- 76.27) | 81.00  (75.50- 86.00) |

**Table S10 The net biopsy reduction rate in the validation cohort.**

| **Thresholds** | **TN** | **TP** | **FN** | **FP** | **FPR** | **TPR** | **TNR** | **FNR** | **BRR** |
| --- | --- | --- | --- | --- | --- | --- | --- | --- | --- |
| 0.05 | 49 | 58 | 2 | 91 | 0.65 | 0.97 | 0.35 | 3.3% | 9.5% |
| 0.1 | 88 | 55 | 5 | 52 | 0.37 | 0.92 | 0.63 | 8.3% | 30.5% |
| 0.2 | 111 | 50 | 10 | 29 | 0.21 | 0.83 | 0.79 | 16.7% | 44.5% |
| 0.3 | 115 | 49 | 11 | 25 | 0.18 | 0.82 | 0.82 | 18.3% | 47.0% |
| 0.4 | 123 | 47 | 13 | 17 | 0.12 | 0.78 | 0.88 | 21.7% | 52.0% |
| 0.4488 | 127 | 46 | 14 | 13 | 0.09 | 0.77 | 0.91 | 23.3% | 54.5% |
| 0.5 | 128 | 45 | 15 | 12 | 0.09 | 0.75 | 0.91 | 25.0% | 55.5% |
| 0.6 | 129 | 42 | 18 | 11 | 0.08 | 0.70 | 0.92 | 30.0% | 57.5% |
| 0.7 | 137 | 39 | 21 | 3 | 0.02 | 0.65 | 0.98 | 35.0% | 63.0% |
| 0.8 | 140 | 26 | 34 | / | 0.00 | 0.43 | 1.00 | 56.7% | 71.0% |
| 0.9 | 140 | 12 | 48 | / | 0.00 | 0.20 | 1.00 | 80.0% | 78.0% |

TN:True Negative; TP: True Positive; FN: False Negative; FP: False Positive;TPR:True Positive Rate; FPR: False Positive Rate;TNR:True Negative Rate;FNR: False Negative Rate; BRR:Biopsy Reduction Rate.

**Table S11 The clinical performance of diagnostic model (*ADD3_2, GSX2_2*) in different PSA levels of high-grade cancer (≥GG2) in validation cohort.**

|  | **All** | | **PSA<4** | | **4≤PSA≤10** | | **PSA>10** | |
| --- | --- | --- | --- | --- | --- | --- | --- | --- |
|  | **Case** | **Control** | **Case** | **Control** | **Case** | **Control** | **Case** | **Control** |
| **Predicted positive** | 45 | 13 | 3 | 7 | 1 | 5 | 41 | 1 |
| **Predicted negative** | 11 | 127 | 1 | 47 | 2 | 45 | 8 | 35 |
| **sensitivity（%）** | 80.36  (69.64- 91.07) | | 75  (25- 100) | | 33.33  (0- 100) | | 83.67  (73.47- 93.88) | |
| **specificity（%）** | 90.71  (85.71- 95) | | 87.04  (77.78- 94.44) | | 90  (82- 98) | | 97.22  (91.67- 100) | |
| **PPV（%）** | 77.78  (68.85- 87.27) | | 30  (12.5- 57.14) | | 16.67  (0- 50) | | 97.67  (92.5- 100) | |
| **NPV（%）** | 92.09  (88.11- 96.15) | | 97.96  (94- 100) | | 95.74  (93.33- 100) | | 81.4  (72- 91.89) | |
| **accuracy（%）** | 87.76  (83.16- 91.85) | | 86.21  (77.59- 94.83) | | 86.79  (77.36- 94.34) | | 89.41  (82.35- 95.29) | |
| **AUC** | 0.92  (0.876- 0.968) | | 0.87  (0.634- 1) | | 0.71  (0.467- 0.959) | | 0.96  (0.922- 0.999) | |

**Table S12. QPCR and related clinical data for the training and validation cohorts.**

| **cohort** | **sampleID** | **PCAID** | ***ADD*_**  **FAM** | ***ADD*_**  **FAM-**  **VIC** | ***GSX*_**  **FAM** | ***GSX*_**  **FAM-**  **VIC** | ***CYB*_**  **FAM** | ***CYB*_**  **FAM-VIC** | **ISUP** | **Prostate Biopsy** | **class** | **Result** | **2gene**  **Predict value** | **2gene**  **predictresult** | **3gene**  **Predict**  **value** | **3gene**  **Predict**  **result** |
| --- | --- | --- | --- | --- | --- | --- | --- | --- | --- | --- | --- | --- | --- | --- | --- | --- |
| Training | 283DRE | PCA230180 | 50 | 27.38 | 37.43 | 15.62 | 29.44 | 6.98 | 0 | 0 | BPH | 0 | 0.0767 | 0 | 0.2636 | 0 |
| Training | 601DRE | PCA230536 | 50 | 27.24 | 35.56 | 12.8 | 50 | 27.24 | 0 | 0 | BPH | 0 | 0.1019 | 0 | 0.0736 | 0 |
| Training | 506DRE | PCA230488 | 50 | 26.24 | 36.93 | 13.17 | 34.11 | 10.35 | 0 | 0 | BPH | 0 | 0.1152 | 0 | 0.2769 | 0 |
| Training | 605DRE | PCA230537 | 50 | 27.79 | 34.07 | 11.86 | 50 | 27.79 | 0 | 0 | OUM | 0 | 0.1019 | 0 | 0.0716 | 0 |
| Training | 581DRE | PCA230527 | 50 | 24.11 | 31.87 | 5.98 | 50 | 24.11 | 0 | 0 | BPH | 0 | 0.2827 | 0 | 0.2225 | 0 |
| Training | 310DRE | PCA230251 | 50 | 23.44 | 50 | 23.24 | 50 | 23.51 | 0 | 0 | BPH | 0 | 0.0708 | 0 | 0.0636 | 0 |
| Training | 486DRE | PCA230416 | 50 | 25.13 | 50 | 25.13 | 50 | 25.13 | 0 | 0 | BPH | 0 | 0.0446 | 0 | 0.0388 | 0 |
| Training | 249DRE | PCA230147 | 50 | 27.54 | 38.61 | 13.05 | 50 | 28.82 | 0 | 0 | BPH | 0 | 0.095 | 0 | 0.0612 | 0 |
| Training | 568DRE | PCA230523 | 38.36 | 13.07 | 36.18 | 10.89 | 50 | 24.71 | 0 | 0 | BPH | 0 | 0.6235 | 1 | 0.403 | 1 |
| Training | 585DRE | PCA230532 | 50 | 26.71 | 50 | 26.71 | 50 | 26.71 | 0 | 0 | BPH | 0 | 0.0293 | 0 | 0.0244 | 0 |
| Training | 257DRE | PCA230153 | 50 | 22.81 | 44.8 | 17.83 | 50 | 23.09 | 0 | 0 | BPH | 0 | 0.1287 | 0 | 0.111 | 0 |
| Training | 587DRE | PCA230535 | 50 | 25.66 | 37.54 | 13.2 | 37.34 | 13 | 0 | 0 | OUM | 0 | 0.1257 | 0 | 0.2453 | 0 |
| Training | 440DRE | PCA230301 | 50 | 23.75 | 50 | 23.75 | 50 | 23.75 | 0 | 0 | BPH | 0 | 0.0641 | 0 | 0.0576 | 0 |
| Training | 579DRE | PCA230552 | 50 | 24.87 | 36.28 | 11.15 | 39.12 | 13.99 | 0 | 0 | BPH | 0 | 0.1691 | 0 | 0.2836 | 0 |
| Training | 604DRE | PCA230540 | 50 | 26.69 | 29.53 | 6.22 | 37.36 | 14.05 | 0 | 0 | OUM | 0 | 0.1965 | 0 | 0.3303 | 0 |
| Training | 261DRE | PCA230120 | 50 | 24.75 | 44 | 17.61 | 50 | 22.29 | 0 | 0 | BPH | 0 | 0.0971 | 0 | 0.0971 | 0 |
| Training | 474DRE | PCA230450 | 50 | 25.29 | 50 | 25.29 | 50 | 25.29 | 0 | 0 | BPH | 0 | 0.0428 | 0 | 0.037 | 0 |
| Training | 210DRE | PCA230177 | 44.78 | 17.73 | 40.99 | 14.21 | 40.97 | 13.29 | 0 | 0 | BPH | 0 | 0.3426 | 0 | 0.434 | 1 |
| Training | 606DRE | PCA230539 | 50 | 26.14 | 36.24 | 12.38 | 50 | 26.14 | 0 | 0 | OUM | 0 | 0.1256 | 0 | 0.0943 | 0 |
| Training | 221DRE | PCA230179 | 50 | 24.64 | 39.37 | 14.09 | 50 | 21.13 | 0 | 0 | BPH | 0 | 0.1357 | 0 | 0.1426 | 0 |
| Training | 265DRE | PCA230100 | 50 | 22.03 | 40.66 | 13.32 | 39.92 | 12.49 | 0 | 0 | BPH | 0 | 0.2116 | 0 | 0.3445 | 1 |
| Training | 562DRE | PCA230530 | 50 | 27.13 | 50 | 27.13 | 50 | 27.13 | 0 | 0 | BPH | 0 | 0.0261 | 0 | 0.0216 | 0 |
| Training | 238DRE | PCA230154 | 50 | 25.26 | 43.97 | 16.81 | 50 | 22.72 | 0 | 0 | BPH | 0 | 0.0964 | 0 | 0.0947 | 0 |
| Training | 242DRE | PCA230168 | 50 | 25.63 | 43.15 | 15.52 | 50 | 23.52 | 0 | 0 | BPH | 0 | 0.1024 | 0 | 0.0949 | 0 |
| Training | 496DRE | PCA230430 | 50 | 26.56 | 39.72 | 16.28 | 50 | 26.56 | 0 | 0 | BPH | 0 | 0.0823 | 0 | 0.0626 | 0 |
| Training | 470DRE | PCA230438 | 38.47 | 14.73 | 34.94 | 11.2 | 37.61 | 13.87 | 0 | 0 | BPH | 0 | 0.5453 | 1 | 0.5809 | 1 |
| Training | 271DRE | PCA230094 | 50 | 24.51 | 39.51 | 14.47 | 50 | 22.69 | 0 | 0 | BPH | 0 | 0.1338 | 0 | 0.1247 | 0 |
| Training | 572DRE | PCA230546 | 39.54 | 16.43 | 36.66 | 13.55 | 27.78 | 4.67 | 0 | 0 | OUM | 0 | 0.4119 | 0 | 0.6707 | 1 |
| Training | 493DRE | PCA230413 | 39.06 | 15.32 | 50 | 26.26 | 34.26 | 10.52 | 0 | 0 | BPH | 0 | 0.1888 | 0 | 0.3049 | 0 |
| Training | 337DRE | PCA230297 | 50 | 23.62 | 39.7 | 13.32 | 50 | 23.62 | 0 | 0 | BPH | 0 | 0.1688 | 0 | 0.1399 | 0 |
| Training | 214DRE | PCA230166 | 42.98 | 16.69 | 47.24 | 20.68 | 50 | 22.30 | 0 | 0 | BPH | 0 | 0.2443 | 0 | 0.1796 | 0 |
| Training | 427DRE | PCA230317 | 50 | 25.85 | 50 | 25.85 | 45.63 | 21.48 | 0 | 0 | BPH | 0 | 0.0368 | 0 | 0.0454 | 0 |
| Training | 608DRE | PCA230513 | 50 | 27.13 | 35.16 | 12.29 | 37.11 | 14.24 | 0 | 0 | OUM | 0 | 0.1086 | 0 | 0.209 | 0 |
| Training | 302DRE | PCA230086 | 50 | 25 | 44.52 | 17.54 | 50 | 23.57 | 0 | 0 | BPH | 0 | 0.0939 | 0 | 0.0857 | 0 |
| Training | 600DRE | PCA230580 | 50 | 26.45 | 36.47 | 12.92 | 50 | 26.45 | 0 | 0 | BPH | 0 | 0.1141 | 0 | 0.085 | 0 |
| Training | 245DRE | PCA230133 | 50 | 26 | 42.54 | 14.96 | 38.01 | 10.97 | 0 | 0 | BPH | 0 | 0.1017 | 0 | 0.2408 | 0 |
| Training | 296DRE | PCA230087 | 50 | 21.41 | 45.41 | 16.71 | 50 | 21.68 | 0 | 0 | BPH | 0 | 0.1748 | 0 | 0.1567 | 0 |
| Training | 308DRE | PCA230090 | 38.95 | 12.12 | 41.3 | 14.3 | 50 | 23.17 | 0 | 0 | BPH | 0 | 0.5801 | 1 | 0.3891 | 1 |
| Training | 237DRE | PCA230135 | 36.19 | 7.23 | 39.76 | 10.83 | 38.85 | 9.88 | 0 | 0 | BPH | 0 | 0.8228 | 1 | 0.8366 | 1 |
| Training | 478DRE | PCA230415 | 50 | 25.19 | 50 | 25.19 | 50 | 25.19 | 0 | 0 | BPH | 0 | 0.0439 | 0 | 0.0381 | 0 |
| Training | 544DRE | PCA230492 | 50 | 26.66 | 35.47 | 12.13 | 50 | 26.66 | 0 | 1 | BPH | 0 | 0.1186 | 0 | 0.0871 | 0 |
| Training | 526DRE | PCA230495 | 37.87 | 13.57 | 50 | 25.7 | 36.95 | 12.65 | 0 | 0 | BPH | 0 | 0.2509 | 0 | 0.3219 | 0 |
| Training | 224DRE | PCA230167 | 50 | 22.97 | 42.27 | 14.83 | 50 | 23.49 | 0 | 0 | BPH | 0 | 0.1633 | 0 | 0.1344 | 0 |
| Training | 180DRE | PCA230077 | 39.04 | 10.1 | 38.64 | 8.72 | 37.08 | 8.347 | 0 | 0 | BPH | 0 | 0.7768 | 1 | 0.833 | 1 |
| Training | 590DRE | PCA230518 | 50 | 24.37 | 43.95 | 18.32 | 45.06 | 19.43 | 0 | 0 | OUM | 0 | 0.0966 | 0 | 0.1196 | 0 |
| Training | 577DRE | PCA230543 | 50 | 25.43 | 40.39 | 15.82 | 50 | 25.43 | 0 | 0 | BPH | 0 | 0.1028 | 0 | 0.0812 | 0 |
| Training | 614DRE | PCA230578 | 50 | 25.78 | 50 | 25.78 | 50 | 25.78 | 0 | 0 | OUM | 0 | 0.0375 | 0 | 0.0321 | 0 |
| Training | 469DRE | PCA230422 | 50 | 26.5 | 39.74 | 16.24 | 50 | 26.5 | 0 | 0 | BPH | 0 | 0.0834 | 0 | 0.0636 | 0 |
| Training | 292DRE | PCA230183 | 50 | 23.94 | 50 | 24.23 | 50 | 22.84 | 0 | 0 | BPH | 0 | 0.0593 | 0 | 0.0583 | 0 |
| Training | 223DRE | PCA230158 | 50 | 21.49 | 50 | 22.85 | 50 | 23.42 | 0 | 0 | BPH | 0 | 0.1004 | 0 | 0.0828 | 0 |
| Training | 231DRE | PCA230175 | 50 | 25.85 | 50 | 23.32 | 50 | 22.42 | 0 | 0 | BPH | 0 | 0.0472 | 0 | 0.0523 | 0 |
| Training | 233DRE | PCA230134 | 50 | 23.93 | 43.9 | 17.96 | 50 | 23.45 | 0 | 0 | BPH | 0 | 0.107 | 0 | 0.0943 | 0 |
| Training | 428DRE | PCA230370 | 50 | 25.81 | 50 | 25.81 | 50 | 25.81 | 0 | 0 | BPH | 0 | 0.0372 | 0 | 0.0318 | 0 |
| Training | 584DRE | PCA230531 | 37.23 | 13.53 | 34.45 | 10.75 | 35.64 | 11.94 | 0 | 0 | BPH | 0 | 0.6078 | 1 | 0.6648 | 1 |
| Training | 321DRE | PCA230282 | 50 | 25.98 | 41.56 | 14.87 | 50 | 23.61 | 0 | 0 | BPH | 0 | 0.1028 | 0 | 0.0956 | 0 |
| Training | 209DRE | PCA230176 | 50 | 24.21 | 48.34 | 22.57 | 50 | 23.34 | 0 | 0 | BPH | 0 | 0.0665 | 0 | 0.0625 | 0 |
| Training | 458DRE | PCA230425 | 50 | 24.14 | 41.07 | 15.21 | 50 | 24.14 | 0 | 0 | BPH | 0 | 0.1326 | 0 | 0.1093 | 0 |
| Training | 503DRE | PCA230476 | 50 | 26.21 | 37.2 | 13.41 | 50 | 26.21 | 0 | 0 | BPH | 0 | 0.1133 | 0 | 0.0854 | 0 |
| Training | 263DRE | PCA230155 | 50 | 27.41 | 37.81 | 14.54 | 50 | 27.60 | 0 | 0 | BPH | 0 | 0.0844 | 0 | 0.0605 | 0 |
| Training | 215DRE | PCA230140 | 50 | 22.85 | 39.09 | 11.58 | 39.89 | 10.69 | 0 | 0 | BPH | 0 | 0.2173 | 0 | 0.3946 | 1 |
| Training | 177DRE | PCA230075 | 40.96 | 16.22 | 48.88 | 21.43 | 50 | 23.15 | 0 | 0 | BPH | 0 | 0.2454 | 0 | 0.1675 | 0 |
| Training | 497DRE | PCA230417 | 50 | 26.62 | 50 | 26.62 | 50 | 26.62 | 0 | 0 | BPH | 0 | 0.03 | 0 | 0.0251 | 0 |
| Training | 535DRE | PCA230462 | 50 | 23.97 | 41.49 | 15.46 | 50 | 23.97 | 0 | 0 | BPH | 0 | 0.1331 | 0 | 0.1105 | 0 |
| Training | 501DRE | PCA230481 | 50 | 26.13 | 38.34 | 14.47 | 50 | 26.13 | 0 | 0 | BPH | 0 | 0.1042 | 0 | 0.0794 | 0 |
| Training | 236DRE | PCA230131 | 40.44 | 13.22 | 45.6 | 19.34 | 50 | 23.01 | 0 | 0 | BPH | 0 | 0.4052 | 0 | 0.2627 | 0 |
| Training | 188DRE | PCA230073 | 50 | 24.09 | 38.95 | 11.09 | 39.9 | 13.38 | 0 | 0 | BPH | 0 | 0.1902 | 0 | 0.3161 | 0 |
| Training | 264DRE | PCA230109 | 50 | 22.17 | 43.04 | 15.58 | 50 | 22.75 | 0 | 0 | BPH | 0 | 0.1722 | 0 | 0.1459 | 0 |
| Training | 222DRE | PCA230178 | 50 | 24.93 | 40.36 | 15.02 | 50 | 25.52 | 0 | 0 | BPH | 0 | 0.1195 | 0 | 0.0912 | 0 |
| Training | 495DRE | PCA230404 | 50 | 24.88 | 50 | 24.88 | 50 | 24.88 | 0 | 0 | BPH | 0 | 0.0477 | 0 | 0.0417 | 0 |
| Training | 252DRE | PCA230152 | 50 | 21.77 | 48.39 | 20.24 | 50 | 21.01 | 0 | 0 | BPH | 0 | 0.1218 | 0 | 0.1203 | 0 |
| Training | 524DRE | PCA230459 | 50 | 26.35 | 50 | 26.35 | 50 | 26.35 | 0 | 0 | BPH | 0 | 0.0322 | 0 | 0.0271 | 0 |
| Training | 552DRE | PCA230595 | 50 | 24.52 | 35.56 | 10.08 | 39.54 | 14.06 | 0 | 0 | BPH | 0 | 0.1944 | 0 | 0.3116 | 0 |
| Training | 299DRE | PCA230116 | 50 | 23.8 | 50 | 23.66 | 50 | 23.67 | 0 | 0 | BPH | 0 | 0.0641 | 0 | 0.0581 | 0 |
| Training | 298DRE | PCA230113 | 42.1 | 15.66 | 38.79 | 12.45 | 50 | 20.89 | 0 | 0 | BPH | 0 | 0.4728 | 1 | 0.3733 | 1 |
| Training | 466DRE | PCA230401 | 38.6 | 14.22 | 50 | 25.62 | 36.36 | 11.98 | 0 | 0 | BPH | 0 | 0.2315 | 0 | 0.3189 | 0 |
| Training | 304DRE | PCA230107 | 50 | 25.03 | 39.59 | 13.09 | 50 | 23.70 | 0 | 0 | BPH | 0 | 0.1397 | 0 | 0.1217 | 0 |
| Training | 176DRE | PCA230076 | 50 | 25.38 | 44.72 | 20.36 | 50 | 23.42 | 0 | 0 | BPH | 0 | 0.0678 | 0 | 0.0656 | 0 |
| Training | 330DRE | PCA230288 | 41.04 | 19.18 | 37.47 | 12.32 | 37.98 | 13.41 | 0 | 0 | BPH | 0 | 0.3289 | 0 | 0.4299 | 1 |
| Training | 344DRE | PCA230292 | 50 | 24.99 | 50 | 25.12 | 50 | 24.36 | 0 | 0 | BPH | 0 | 0.0457 | 0 | 0.0421 | 0 |
| Training | 607DRE | PCA230579 | 50 | 27.28 | 40.03 | 17.31 | 50 | 27.28 | 0 | 0 | OUM | 0 | 0.0664 | 0 | 0.0497 | 0 |
| Training | 612DRE | PCA230573 | 38.8 | 16.04 | 35.58 | 12.82 | 38.23 | 15.47 | 0 | 0 | BPH | 0 | 0.4469 | 0 | 0.4694 | 1 |
| Training | 530DRE | PCA230452 | 50 | 26.03 | 41.08 | 17.11 | 50 | 26.03 | 0 | 0 | BPH | 0 | 0.0829 | 0 | 0.0648 | 0 |
| Training | 516DRE | PCA230480 | 50 | 25.57 | 49.79 | 25.36 | 50 | 25.57 | 0 | 0 | BPH | 0 | 0.0405 | 0 | 0.0347 | 0 |
| Training | 294DRE | PCA230082 | 50 | 23.12 | 41.84 | 14.76 | 50 | 23.13 | 0 | 0 | BPH | 0 | 0.1607 | 0 | 0.1367 | 0 |
| Training | 550DRE | PCA230591 | 50 | 24.21 | 50 | 24.21 | 50 | 24.21 | 0 | 0 | BPH | 0 | 0.0569 | 0 | 0.0505 | 0 |
| Training | 187DRE | PCA230045 | 37.46 | 7.94 | 42.24 | 15.61 | 50 | 20.57 | 0 | 0 | BPH | 0 | 0.7157 | 1 | 0.5428 | 1 |
| Training | 301DRE | PCA230084 | 50 | 23.75 | 47.79 | 21.38 | 50 | 23.78 | 0 | 0 | BPH | 0 | 0.0802 | 0 | 0.0706 | 0 |
| Training | 228DRE | PCA230162 | 50 | 26.73 | 39.56 | 14.63 | 50 | 23.23 | 0 | 0 | BPH | 0 | 0.0934 | 0 | 0.0924 | 0 |
| Training | 595DRE | PCA230584 | 50 | 26.32 | 50 | 26.32 | 50 | 26.32 | 0 | 0 | BPH | 0 | 0.0325 | 0 | 0.0274 | 0 |
| Training | 303DRE | PCA230101 | 50 | 22.81 | 50 | 22.6 | 50 | 22.66 | 0 | 0 | BPH | 0 | 0.0833 | 0 | 0.0774 | 0 |
| Training | 334DRE | PCA230266 | 50 | 23.27 | 50 | 24.14 | 50 | 23.11 | 0 | 0 | BPH | 0 | 0.0668 | 0 | 0.0621 | 0 |
| Training | 487DRE | PCA230432 | 50 | 25.97 | 37.55 | 13.52 | 50 | 25.97 | 0 | 0 | BPH | 0 | 0.1164 | 0 | 0.0887 | 0 |
| Training | 512DRE | PCA230489 | 50 | 26.97 | 39.33 | 16.3 | 37.3 | 14.27 | 0 | 0 | BPH | 0 | 0.0769 | 0 | 0.1572 | 0 |
| Training | 499DRE | PCA230407 | 37.55 | 12.21 | 41.95 | 16.61 | 38.39 | 13.05 | 0 | 0 | BPH | 0 | 0.518 | 1 | 0.5537 | 1 |
| Training | 473DRE | PCA230448 | 50 | 25.47 | 44.98 | 20.45 | 38.2 | 13.67 | 0 | 0 | BPH | 0 | 0.0662 | 0 | 0.1394 | 0 |
| Training | 220DRE | PCA230145 | 50 | 24.77 | 43.67 | 18.27 | 50 | 23.88 | 0 | 0 | BPH | 0 | 0.0911 | 0 | 0.0808 | 0 |
| Training | 498DRE | PCA230426 | 50 | 26.65 | 50 | 26.65 | 50 | 26.65 | 0 | 0 | BPH | 0 | 0.0297 | 0 | 0.0248 | 0 |
| Training | 208DRE | PCA230071 | 40.97 | 14.91 | 40.51 | 15.53 | 39.26 | 15.73 | 0 | 0 | BPH | 0 | 0.4276 | 0 | 0.4369 | 1 |
| Training | 502DRE | PCA230490 | 37.89 | 13.3 | 34.13 | 9.54 | 50 | 25.41 | 0 | 0 | BPH | 0 | 0.6461 | 1 | 0.4111 | 1 |
| Training | 256DRE | PCA230144 | 50 | 24.04 | 43.87 | 16.79 | 50 | 23.12 | 0 | 0 | BPH | 0 | 0.1169 | 0 | 0.1052 | 0 |
| Training | 251DRE | PCA230150 | 40.26 | 12.49 | 41.3 | 13.22 | 38.03 | 10.26 | 0 | 0 | BPH | 0 | 0.5911 | 1 | 0.6759 | 1 |
| Training | 243DRE | PCA230172 | 50 | 23.05 | 42.69 | 15.44 | 50 | 23.20 | 0 | 0 | BPH | 0 | 0.1531 | 0 | 0.1298 | 0 |
| Training | 509DRE | PCA230475 | 50 | 26.68 | 50 | 26.68 | 50 | 26.68 | 0 | 0 | BPH | 0 | 0.0295 | 0 | 0.0246 | 0 |
| Training | 536DRE | PCA230469 | 50 | 25 | 50 | 25 | 50 | 25 | 0 | 0 | BPH | 0 | 0.0462 | 0 | 0.0402 | 0 |
| Training | 523DRE | PCA230484 | 50 | 26.62 | 37.92 | 14.54 | 50 | 26.62 | 0 | 0 | BPH | 0 | 0.0958 | 0 | 0.0717 | 0 |
| Training | 315DRE | PCA230254 | 39.53 | 14.63 | 42.71 | 16.92 | 37.96 | 10.70 | 0 | 0 | BPH | 0 | 0.4051 | 0 | 0.524 | 1 |
| Training | 609DRE | PCA230515 | 50 | 27.71 | 34.46 | 12.17 | 50 | 27.71 | 0 | 0 | BPH | 0 | 0.1003 | 0 | 0.0708 | 0 |
| Training | 225DRE | PCA230165 | 50 | 23.35 | 39.25 | 13 | 50 | 22.971 | 0 | 0 | BPH | 0 | 0.1803 | 0 | 0.1548 | 0 |
| Training | 262DRE | PCA230148 | 50 | 22.78 | 42 | 16 | 50 | 23.74 | 0 | 0 | BPH | 0 | 0.1519 | 0 | 0.1226 | 0 |
| Training | 518DRE | PCA230486 | 50 | 25.98 | 37.62 | 13.6 | 50 | 25.98 | 0 | 0 | BPH | 0 | 0.1154 | 0 | 0.0879 | 0 |
| Training | 589DRE | PCA230589 | 50 | 27.64 | 37.06 | 14.7 | 50 | 27.64 | 0 | 0 | BPH | 0 | 0.0802 | 0 | 0.0579 | 0 |
| Training | 467DRE | PCA230412 | 50 | 24.75 | 43.38 | 18.13 | 50 | 24.75 | 0 | 0 | BPH | 0 | 0.0925 | 0 | 0.0763 | 0 |
| Training | 343DRE | PCA230273 | 50 | 28.24 | 38.65 | 14.74 | 50 | 25.79 | 0 | 0 | BPH | 0 | 0.0725 | 0 | 0.0627 | 0 |
| Training | 275DRE | PCA230115 | 50 | 22.03 | 39.2 | 13.36 | 50 | 24.34 | 0 | 0 | BPH | 0 | 0.211 | 0 | 0.1561 | 0 |
| Training | 293DRE | PCA230099 | 38.77 | 12 | 50 | 23.4 | 50 | 21.75 | 0 | 0 | BPH | 0 | 0.358 | 0 | 0.2421 | 0 |
| Training | 449DRE | PCA230379 | 50 | 26.16 | 50 | 26.16 | 50 | 26.16 | 0 | 0 | BPH | 0 | 0.0339 | 0 | 0.0287 | 0 |
| Training | 500DRE | PCA230468 | 50 | 26.06 | 36.22 | 12.28 | 50 | 26.06 | 0 | 0 | BPH | 0 | 0.1283 | 0 | 0.0966 | 0 |
| Training | 597DRE | PCA230590 | 50 | 26.65 | 36.74 | 13.39 | 36.54 | 13.19 | 0 | 0 | BPH | 0 | 0.106 | 0 | 0.2176 | 0 |
| Training | 537DRE | PCA230472 | 50 | 25.42 | 35.86 | 11.28 | 35.7 | 11.12 | 0 | 0 | BPH | 0 | 0.1542 | 0 | 0.3199 | 0 |
| Training | 203DRE | PCA230065 | 50 | 21.74 | 44.86 | 16.27 | 50 | 19.74 | 0 | 0 | BPH | 0 | 0.1729 | 0 | 0.1804 | 0 |
| Training | 284DRE | PCA230180A | 50 | 24.12 | 42.61 | 17.56 | 42.88 | 15.16 | 0 | 0 | BPH | 0 | 0.1077 | 0 | 0.1792 | 0 |
| Training | 564DRE | PCA230554 | 50 | 26.21 | 50 | 26.21 | 50 | 26.21 | 0 | 0 | BPH | 0 | 0.0335 | 0 | 0.0283 | 0 |
| Training | 569DRE | PCA230528 | 50 | 26.81 | 50 | 26.81 | 50 | 26.81 | 0 | 0 | BPH | 0 | 0.0285 | 0 | 0.0237 | 0 |
| Training | 217DRE | PCA230171 | 50 | 23.43 | 43.81 | 17.17 | 50 | 22.01 | 0 | 0 | BPH | 0 | 0.1242 | 0 | 0.119 | 0 |
| Training | 513DRE | PCA230466 | 50 | 26.74 | 50 | 26.74 | 36.36 | 13.1 | 0 | 0 | BPH | 0 | 0.029 | 0 | 0.0759 | 0 |
| Training | 588DRE | PCA230586 | 34.71 | 12.47 | 35.97 | 13.73 | 33.12 | 10.88 | 0 | 0 | BPH | 0 | 0.5794 | 1 | 0.654 | 1 |
| Training | 457DRE | PCA230423 | 50 | 26.13 | 35.42 | 11.55 | 39.17 | 15.3 | 0 | 0 | BPH | 0 | 0.1354 | 0 | 0.2256 | 0 |
| Training | 268DRE | PCA230121 | 40.6 | 12.63 | 41.55 | 15.29 | 40.18 | 14.89 | 0 | 0 | BPH | 0 | 0.5332 | 1 | 0.5306 | 1 |
| Training | 240DRE | PCA230159 | 50 | 25.7 | 45.52 | 20.66 | 50 | 23.58 | 0 | 0 | BPH | 0 | 0.0625 | 0 | 0.0608 | 0 |
| Training | 464DRE | PCA230418 | 50 | 25.72 | 36.68 | 12.4 | 50 | 25.72 | 0 | 0 | BPH | 0 | 0.1337 | 0 | 0.102 | 0 |
| Training | 348DRE | PCA230253 | 50 | 23.98 | 38.68 | 14.03 | 50 | 27.55 | 0 | 0 | BPH | 0 | 0.1506 | 0 | 0.0936 | 0 |
| Training | 438DRE | PCA230312 | 50 | 27.05 | 37.71 | 14.76 | 50 | 27.05 | 0 | 0 | BPH | 0 | 0.0877 | 0 | 0.0647 | 0 |
| Training | 472DRE | PCA230445 | 50 | 24.86 | 37.24 | 12.1 | 37.69 | 12.55 | 0 | 0 | BPH | 0 | 0.1562 | 0 | 0.292 | 0 |
| Training | 485DRE | PCA230414 | 50 | 25.46 | 50 | 25.46 | 50 | 25.46 | 0 | 1 | BPH | 0 | 0.0409 | 0 | 0.0352 | 0 |
| Training | 207DRE | PCA230058 | 50 | 23.64 | 39.82 | 13.51 | 50 | 22.56 | 0 | 0 | BPH | 0 | 0.1656 | 0 | 0.1489 | 0 |
| Training | 327DRE | PCA230263 | 50 | 23.29 | 40.79 | 14.18 | 41.93 | 15.21 | 0 | 0 | BPH | 0 | 0.1647 | 0 | 0.2468 | 0 |
| Training | 515DRE | PCA230471 | 38.14 | 13.79 | 39.75 | 15.4 | 37.38 | 13.03 | 0 | 0 | BPH | 0 | 0.4796 | 1 | 0.5333 | 1 |
| Training | 181DRE | PCA230049 | 50 | 20.91 | 49.95 | 19.45 | 50 | 21.22 | 0 | 0 | BPH | 0 | 0.1488 | 0 | 0.1381 | 0 |
| Training | 599DRE | PCA230576 | 40.57 | 17.91 | 35.08 | 12.42 | 50 | 27.34 | 0 | 0 | BPH | 0 | 0.3774 | 0 | 0.2045 | 0 |
| Training | 522DRE | PCA230477 | 50 | 24.59 | 36.17 | 10.76 | 50 | 24.59 | 0 | 0 | BPH | 0 | 0.182 | 0 | 0.1434 | 0 |
| Training | 226DRE | PCA230141 | 50 | 24.46 | 43.27 | 17.78 | 50 | 17.41 | 0 | 1 | BPH | 0 | 0.1001 | 0 | 0.1441 | 0 |
| Training | 350DRE | PCA230274 | 50 | 26.6 | 43.1 | 17.69 | 48.54 | 23.8 | 0 | 1 | BPH | 0 | 0.0716 | 0 | 0.0693 | 0 |
| Training | 246DRE | PCA230137 | 50 | 23.16 | 46.03 | 20.17 | 50 | 22.31 | 0 | 1 | BPH | 0 | 0.0987 | 0 | 0.0937 | 0 |
| Training | 193DRE | PCA230059 | 33.51 | 4.45 | 33.68 | 4.66 | 36.98 | 8.01 | 0 | 1 | BPH | 0 | 0.9342 | 1 | 0.9371 | 1 |
| Training | 481DRE | PCA230409 | 50 | 23.7 | 50 | 23.7 | 50 | 23.7 | 0 | 1 | OUM | 0 | 0.0649 | 0 | 0.0585 | 0 |
| Training | 333DRE | PCA230255 | 50 | 24.69 | 40.41 | 14.91 | 50 | 24.80 | 0 | 1 | BPH | 0 | 0.1252 | 0 | 0.1001 | 0 |
| Training | 432DRE | PCA230344 | 50 | 26.4 | 35.74 | 12.14 | 50 | 26.4 | 0 | 1 | BPH | 0 | 0.1233 | 0 | 0.0915 | 0 |
| Training | 525DRE | PCA230460 | 50 | 25.95 | 36.6 | 12.55 | 50 | 25.95 | 0 | 1 | BPH | 0 | 0.1274 | 0 | 0.0964 | 0 |
| Training | 325DRE | PCA230295 | 50 | 26.03 | 41.66 | 15.14 | 50 | 24.40 | 0 | 1 | BPH | 0 | 0.0995 | 0 | 0.0873 | 0 |
| Training | 532DRE | PCA230455 | 37.82 | 13.58 | 34.32 | 10.08 | 35.42 | 11.18 | 0 | 1 | BPH | 0 | 0.6219 | 1 | 0.6913 | 1 |
| Training | 459DRE | PCA230434 | 50 | 26.41 | 50 | 26.41 | 50 | 26.41 | 0 | 1 | BPH | 0 | 0.0317 | 0 | 0.0267 | 0 |
| Training | 289DRE | PCA230114 | 50 | 22.3 | 50 | 22.6 | 50 | 22.09 | 0 | 1 | BPH | 0 | 0.0904 | 0 | 0.0859 | 0 |
| Training | 539DRE | PCA230494 | 50 | 26.14 | 50 | 26.14 | 50 | 26.14 | 0 | 1 | BPH | 0 | 0.0341 | 0 | 0.0289 | 0 |
| Training | 566DRE | PCA230551 | 35.11 | 13.69 | 33.7 | 12.28 | 34.12 | 12.7 | 0 | 1 | OUM | 0 | 0.5632 | 1 | 0.6127 | 1 |
| Training | 602DRE | PCA230511 | 50 | 26.91 | 34.97 | 11.88 | 37.36 | 14.27 | 0 | 1 | BPH | 0 | 0.1167 | 0 | 0.2194 | 0 |
| Training | 567DRE | PCA230597 | 50 | 27.09 | 50 | 27.09 | 50 | 27.09 | 0 | 1 | OUM | 0 | 0.0264 | 0 | 0.0218 | 0 |
| Training | 317DRE | PCA230285 | 50 | 25.16 | 50 | 22.69 | 50 | 26.63 | 0 | 1 | BPH | 0 | 0.0562 | 0 | 0.0421 | 0 |
| Training | 307DRE | PCA230089 | 50 | 23.81 | 50 | 23.64 | 50 | 24.18 | 0 | 1 | BPH | 0 | 0.0642 | 0 | 0.0558 | 0 |
| Training | 563DRE | PCA230545 | 50 | 26.63 | 40.55 | 17.18 | 50 | 26.63 | 0 | 1 | BPH | 0 | 0.0748 | 0 | 0.0572 | 0 |
| Training | 266DRE | PCA230111 | 50 | 22.96 | 40.61 | 14.24 | 50 | 23.93 | 0 | 1 | BPH | 0 | 0.1719 | 0 | 0.1363 | 0 |
| Training | 250DRE | PCA230149 | 50 | 22.67 | 42.97 | 17.05 | 50 | 23.52 | 0 | 1 | BPH | 0 | 0.1409 | 0 | 0.116 | 0 |
| Training | 482DRE | PCA230441 | 50 | 25.97 | 35.36 | 11.33 | 50 | 25.97 | 0 | 1 | OUM | 0 | 0.1415 | 0 | 0.1062 | 0 |
| Training | 189DRE | PCA230048 | 38.61 | 12.99 | 46.88 | 19.32 | 50 | 24.02 | 0 | 1 | BPH | 0 | 0.4155 | 0 | 0.2516 | 0 |
| Training | 555DRE | PCA230521 | 50 | 25.98 | 33.52 | 9.5 | 37.23 | 13.21 | 0 | 1 | OUM | 0 | 0.1654 | 0 | 0.3006 | 0 |
| Training | 534DRE | PCA230457 | 50 | 24.69 | 38.1 | 12.79 | 36.29 | 10.98 | 0 | 1 | BPH | 0 | 0.1509 | 0 | 0.3122 | 0 |
| Training | 593DRE | PCA230542 | 50 | 27 | 36.78 | 13.78 | 50 | 27 | 0 | 1 | BPH | 0 | 0.0967 | 0 | 0.071 | 0 |
| Training | 272DRE | PCA230095 | 50 | 26.74 | 39.95 | 17.51 | 50 | 26.73 | 0 | 1 | BPH | 0 | 0.0712 | 0 | 0.0544 | 0 |
| Training | 305DRE | PCA230119 | 50 | 25.21 | 39.73 | 12.62 | 50 | 23.77 | 0 | 1 | BPH | 0 | 0.1417 | 0 | 0.1233 | 0 |
| Training | 316DRE | PCA230284 | 50 | 23.67 | 41.41 | 15.01 | 50 | 25.02 | 0 | 1 | BPH | 0 | 0.1449 | 0 | 0.1091 | 0 |
| Training | 546DRE | PCA230497 | 50 | 25.72 | 36.49 | 12.21 | 50 | 25.72 | 0 | 1 | BPH | 0 | 0.136 | 0 | 0.1036 | 0 |
| Training | 460DRE | PCA230436 | 50 | 26.7 | 38.44 | 15.14 | 50 | 26.7 | 0 | 1 | BPH | 0 | 0.0895 | 0 | 0.0671 | 0 |
| Training | 338DRE | PCA230258 | 50 | 24.2 | 50 | 24.2 | 50 | 24.2 | 0 | 1 | BPH | 0 | 0.057 | 0 | 0.0507 | 0 |
| Training | 285DRE | PCA230181 | 50 | 22.51 | 50 | 22.75 | 50 | 21.73 | 0 | 1 | BPH | 0 | 0.0862 | 0 | 0.0852 | 0 |
| Training | 451DRE | PCA230315 | 50 | 25.77 | 35.26 | 11.03 | 50 | 25.77 | 0 | 1 | BPH | 0 | 0.1496 | 0 | 0.1129 | 0 |
| Training | 241DRE | PCA230161 | 50 | 24.62 | 44.07 | 16.02 | 50 | 21.24 | 0 | 1 | BPH | 0 | 0.1146 | 0 | 0.1217 | 0 |
| Training | 230DRE | PCA230170 | 50 | 21.91 | 41.64 | 12.75 | 50 | 22.30 | 0 | 1 | BPH | 0 | 0.2251 | 0 | 0.1919 | 0 |
| Training | 471DRE | PCA230439 | 50 | 25.42 | 50 | 25.42 | 50 | 25.42 | 0 | 1 | BPH | 0 | 0.0413 | 0 | 0.0356 | 0 |
| Training | 295DRE | PCA230085 | 50 | 21.97 | 42.11 | 13.9 | 50 | 22.07 | 0 | 1 | BPH | 0 | 0.2036 | 0 | 0.178 | 0 |
| Training | 504DRE | PCA230478 | 38.41 | 13.84 | 50 | 25.43 | 37.14 | 12.57 | 0 | 1 | BPH | 0 | 0.2472 | 0 | 0.3216 | 0 |
| Training | 455DRE | PCA230442 | 50 | 23.01 | 38.25 | 11.26 | 50 | 23.01 | 0 | 0 | OUM | 0 | 0.2181 | 0 | 0.1824 | 0 |
| Training | 533DRE | PCA230456 | 50 | 26.23 | 38.95 | 15.18 | 37.09 | 13.32 | 0 | 1 | BPH | 0 | 0.0961 | 0 | 0.1974 | 0 |
| Training | 336DRE | PCA230281 | 50 | 25.34 | 39.56 | 13.82 | 50 | 25.38 | 0 | 1 | BPH | 0 | 0.1249 | 0 | 0.0972 | 0 |
| Training | 211DRE | PCA230067 | 50 | 23.95 | 43.7 | 17.21 | 50 | 23.22 | 0 | 1 | BPH | 0 | 0.1142 | 0 | 0.1019 | 0 |
| Training | 253DRE | PCA230157 | 50 | 23.49 | 42.98 | 18.86 | 50 | 23.41 | 0 | 1 | BPH | 0 | 0.1056 | 0 | 0.0922 | 0 |
| Training | 281DRE | PCA230093 | 50 | 22.6 | 38.24 | 10.85 | 50 | 22.48 | 0 | 1 | BPH | 0 | 0.2381 | 0 | 0.2033 | 0 |
| Training | 247DRE | PCA230139 | 50 | 23.43 | 45.93 | 18.74 | 50 | 23.56 | 0 | 1 | BPH | 0 | 0.1078 | 0 | 0.0927 | 0 |
| Training | 258DRE | PCA230102 | 36.31 | 11.12 | 36.99 | 10.2 | 36.26 | 8.959 | 0 | 1 | BPH | 0 | 0.7144 | 1 | 0.7847 | 1 |
| Training | 583DRE | PCA230553 | 39.23 | 15.36 | 38.42 | 14.55 | 37.57 | 13.7 | 0 | 1 | BPH | 0 | 0.4328 | 0 | 0.4897 | 1 |
| Training | 206DRE | PCA230078 | 50 | 24.03 | 38.73 | 12.63 | 50 | 23.62 | 0 | 1 | BPH | 0 | 0.1686 | 0 | 0.1414 | 0 |
| Training | 463DRE | PCA230424 | 50 | 25.68 | 37.27 | 12.95 | 50 | 25.68 | 0 | 1 | OUM | 0 | 0.1281 | 0 | 0.0982 | 0 |
| Training | 286DRE | PCA230182 | 50 | 23.24 | 48.08 | 22.14 | 44.71 | 17.74 | 0 | 1 | BPH | 0 | 0.0811 | 0 | 0.1134 | 0 |
| Training | 441DRE | PCA230324 | 37.58 | 13.17 | 36.68 | 12.27 | 37.83 | 13.42 | 0 | 1 | BPH | 0 | 0.5857 | 1 | 0.6131 | 1 |
| Training | 447DRE | PCA230323 | 50 | 26.42 | 50 | 26.42 | 50 | 26.42 | 0 | 1 | BPH | 0 | 0.0316 | 0 | 0.0266 | 0 |
| Training | 480DRE | PCA230428 | 50 | 25.95 | 50 | 25.95 | 39.47 | 15.42 | 0 | 1 | BPH | 0 | 0.0359 | 0 | 0.0735 | 0 |
| Training | 527DRE | PCA230491 | 50 | 26.63 | 35.64 | 12.27 | 50 | 26.63 | 0 | 1 | BPH | 0 | 0.1176 | 0 | 0.0866 | 0 |
| Training | 179DRE | PCA230031 | 50 | 26.22 | 36.53 | 13.99 | 50 | 25.04 | 0 | 1 | BPH | 0 | 0.1073 | 0 | 0.0893 | 0 |
| Training | 521DRE | PCA230474 | 50 | 25.53 | 37.62 | 13.15 | 37.45 | 12.98 | 0 | 1 | BPH | 0 | 0.1288 | 0 | 0.2494 | 0 |
| Training | 291DRE | PCA230181A | 50 | 22.31 | 44.65 | 16.95 | 50 | 21.8 | 0 | 1 | BPH | 0 | 0.15 | 0 | 0.1387 | 0 |
| Training | 229DRE | PCA230164 | 50 | 28.14 | 43.16 | 15.96 | 50 | 22.57 | 0 | 1 | BPH | 0 | 0.0656 | 0 | 0.0744 | 0 |
| Training | 465DRE | PCA230410 | 50 | 25.81 | 50 | 25.81 | 37.57 | 13.38 | 0 | 1 | BPH | 0 | 0.0372 | 0 | 0.0891 | 0 |
| Training | 494DRE | PCA230405 | 50 | 26.51 | 36.17 | 12.68 | 37.4 | 13.91 | 0 | 1 | BPH | 0 | 0.1155 | 0 | 0.2208 | 0 |
| Training | 594DRE | PCA230582 | 50 | 26.85 | 38.77 | 15.62 | 50 | 26.85 | 0 | 1 | BPH | 0 | 0.0835 | 0 | 0.0626 | 0 |
| Training | 450DRE | PCA230361 | 50 | 26.61 | 50 | 26.61 | 50 | 26.61 | 0 | 1 | BPH | 0 | 0.03 | 0 | 0.0251 | 0 |
| Training | 320DRE | PCA230278 | 37.86 | 11.36 | 38 | 11.51 | 37.23 | 12.61 | 0 | 1 | BPH | 0 | 0.6773 | 1 | 0.695 | 1 |
| Training | 575DRE | PCA230556 | 50 | 26.4 | 50 | 26.4 | 50 | 26.4 | 0 | 1 | BPH | 0 | 0.0318 | 0 | 0.0267 | 0 |
| Training | 274DRE | PCA230097 | 50 | 23.03 | 41.29 | 15.67 | 50 | 23.10 | 0 | 1 | BPH | 0 | 0.1506 | 0 | 0.1287 | 0 |
| Training | 309DRE | PCA230283 | 49.25 | 22.06 | 50 | 22.74 | 50 | 22.85 | 0 | 1 | BPH | 0 | 0.0927 | 0 | 0.082 | 0 |
| Training | 347DRE | PCA230250 | 50 | 24.38 | 39.16 | 13.22 | 40.02 | 13.39 | 0 | 1 | BPH | 0 | 0.1522 | 0 | 0.2684 | 0 |
| Training | 273DRE | PCA230096 | 50 | 22.92 | 50 | 23.03 | 50 | 23.03 | 0 | 1 | BPH | 0 | 0.0786 | 0 | 0.0715 | 0 |
| Training | 349DRE | PCA230261 | 50 | 24.63 | 40.44 | 14.67 | 50 | 26.83 | 0 | 1 | BPH | 0 | 0.1291 | 0 | 0.0874 | 0 |
| Training | 288DRE | PCA230112 | 50 | 22.4 | 37.87 | 12.31 | 50 | 22.10 | 0 | 1 | BPH | 0 | 0.2181 | 0 | 0.1914 | 0 |
| Training | 479DRE | PCA230429 | 50 | 25.89 | 50 | 25.89 | 50 | 25.89 | 0 | 1 | BPH | 0 | 0.0365 | 0 | 0.0311 | 0 |
| Training | 297DRE | PCA230091 | 50 | 22.89 | 50 | 22.72 | 50 | 22.99 | 0 | 1 | BPH | 0 | 0.0813 | 0 | 0.0739 | 0 |
| Training | 592DRE | PCA230538 | 50 | 26.8 | 35.67 | 12.47 | 38.72 | 15.52 | 0 | 1 | BPH | 0 | 0.1125 | 0 | 0.1948 | 0 |
| Training | 436DRE | PCA230314 | 36.51 | 12.55 | 36.44 | 12.48 | 36.87 | 12.91 | 0 | 1 | BPH | 0 | 0.6067 | 1 | 0.637 | 1 |
| Training | 205DRE | PCA230043 | 50 | 24.97 | 39.88 | 14.71 | 50 | 26.09 | 0 | 1 | BPH | 0 | 0.1221 | 0 | 0.089 | 0 |
| Training | 520DRE | PCA230473 | 35.72 | 12.68 | 50 | 26.96 | 37.48 | 14.44 | 0 | 1 | BPH | 0 | 0.2561 | 0 | 0.2875 | 0 |
| Training | 267DRE | PCA230117 | 50 | 23.89 | 50 | 24.88 | 50 | 23.93 | 0 | 1 | BPH | 0 | 0.0562 | 0 | 0.0507 | 0 |
| Training | 430DRE | PCA230378 | 50 | 25.55 | 45.82 | 21.37 | 50 | 25.55 | 0 | 1 | BPH | 0 | 0.0599 | 0 | 0.0494 | 0 |
| Training | 437DRE | PCA230380 | 50 | 25.92 | 50 | 25.92 | 50 | 25.92 | 0 | 1 | BPH | 0 | 0.0362 | 0 | 0.0308 | 0 |
| Training | 260DRE | PCA230110 | 50 | 24.95 | 39.68 | 11.39 | 50 | 24.06 | 0 | 1 | BPH | 0 | 0.1638 | 0 | 0.1367 | 0 |
| Training | 570DRE | PCA230592 | 34.66 | 11.21 | 34.37 | 10.92 | 34.67 | 11.22 | 0 | 1 | BPH | 0 | 0.6959 | 1 | 0.7346 | 1 |
| Training | 335DRE | PCA230267 | 50 | 24.16 | 43.86 | 17.84 | 50 | 24.65 | 0 | 1 | BPH | 0 | 0.1043 | 0 | 0.0843 | 0 |
| Training | 508DRE | PCA230467 | 34.61 | 10.53 | 48.51 | 24.43 | 35 | 10.92 | 0 | 1 | BPH | 0 | 0.3939 | 0 | 0.4742 | 1 |
| Training | 248DRE | PCA230146 | 50 | 22.49 | 50 | 22.33 | 50 | 23.16 | 0 | 1 | BPH | 0 | 0.0899 | 0 | 0.0788 | 0 |
| Training | 543DRE | PCA230458 | 50 | 26.37 | 37.32 | 13.69 | 50 | 26.37 | 0 | 1 | BPH | 0 | 0.1077 | 0 | 0.0809 | 0 |
| Training | 477DRE | PCA230408 | 50 | 26.35 | 35.65 | 12 | 50 | 26.35 | 0 | 1 | BPH | 0 | 0.1258 | 0 | 0.0935 | 0 |
| Training | 426DRE | PCA230308 | 50 | 25.61 | 50 | 25.61 | 50 | 25.61 | 0 | 1 | BPH | 0 | 0.0393 | 0 | 0.0337 | 0 |
| Training | 259DRE | PCA230106 | 50 | 24.71 | 39.15 | 11.97 | 50 | 22.76 | 0 | 1 | BPH | 0 | 0.1614 | 0 | 0.1478 | 0 |
| Training | 342DRE | PCA230271 | 50 | 25.22 | 40.95 | 15.76 | 50 | 23.13 | 0 | 1 | BPH | 0 | 0.1068 | 0 | 0.1004 | 0 |
| Training | 424DRE | PCA230366 | 50 | 26.72 | 50 | 26.72 | 50 | 26.72 | 0 | 1 | BPH | 0 | 0.0292 | 0 | 0.0243 | 0 |
| Training | 232DRE | PCA230132 | 50 | 26.29 | 45.44 | 18.1 | 50 | 23.53 | 0 | 1 | BPH | 0 | 0.0724 | 0 | 0.0709 | 0 |
| Training | 542DRE | PCA230498 | 39.98 | 15.8 | 38.39 | 14.21 | 36.72 | 12.54 | 0 | 1 | BPH | 0 | 0.4224 | 0 | 0.5094 | 1 |
| Training | 422DRE | PCA230319 | 35.38 | 11.34 | 32.08 | 8.04 | 35.48 | 11.44 | 0 | 1 | BPH | 0 | 0.75 | 1 | 0.7764 | 1 |
| Training | 578DRE | PCA230544 | 50 | 24.67 | 36.44 | 11.11 | 50 | 24.67 | 0 | 1 | OUM | 0 | 0.1747 | 0 | 0.1375 | 0 |
| Training | 574DRE | PCA230600 | 50 | 26.62 | 50 | 26.62 | 50 | 26.62 | 0 | 1 | BPH | 0 | 0.03 | 0 | 0.0251 | 0 |
| Training | 580DRE | PCA230588 | 50 | 26.41 | 38.5 | 14.91 | 38.02 | 14.43 | 0 | 1 | BPH | 0 | 0.0957 | 0 | 0.1828 | 0 |
| Training | 549DRE | PCA230525 | 50 | 26.79 | 40 | 16.79 | 37.67 | 14.46 | 0 | 1 | BPH | 0 | 0.0756 | 0 | 0.1521 | 0 |
| Training | 545DRE | PCA230493 | 38.05 | 14.15 | 50 | 26.1 | 50 | 26.1 | 0 | 1 | BPH | 0 | 0.225 | 0 | 0.1157 | 0 |
| Training | 255DRE | PCA230138 | 50 | 22.61 | 38.97 | 11.95 | 50 | 22.80 | 0 | 1 | BPH | 0 | 0.218 | 0 | 0.1831 | 0 |
| Training | 517DRE | PCA230483 | 50 | 26.66 | 50 | 26.66 | 37.41 | 14.07 | 0 | 1 | BPH | 0 | 0.0296 | 0 | 0.0713 | 0 |
| Training | 213DRE | PCA230174 | 50 | 23.56 | 40.04 | 13.44 | 50 | 23.41 | 0 | 1 | BPH | 0 | 0.1686 | 0 | 0.1416 | 0 |
| Training | 557DRE | PCA230529 | 38.79 | 15.71 | 50 | 26.92 | 36.56 | 13.48 | 5 | 1 | PCa | 1 | 0.1688 | 0 | 0.2329 | 0 |
| Training | 339DRE | PCA230269 | 50 | 26.59 | 39.75 | 14.22 | 50 | 25.51 | 3 | 1 | PCa | 1 | 0.0991 | 0 | 0.0809 | 0 |
| Training | 212DRE | PCA230173 | 28.82 | 2.51 | 29.12 | 2.58 | 30.33 | 4.54 | 5 | 1 | PCa | 1 | 0.961 | 1 | 0.9688 | 1 |
| Training | 280DRE | PCA230108 | 35.95 | 15.21 | 35.61 | 12.04 | 38.12 | 12.38 | 4 | 1 | PCa | 1 | 0.503 | 1 | 0.5798 | 1 |
| Training | 591DRE | PCA230534 | 39.07 | 14.99 | 38.55 | 14.47 | 38.53 | 14.45 | 5 | 1 | PCa | 1 | 0.4508 | 1 | 0.4864 | 1 |
| Training | 433DRE | PCA230321 | 37.69 | 9.65 | 37.54 | 9.5 | 37.43 | 9.39 | 3 | 1 | PCa | 1 | 0.7767 | 1 | 0.8176 | 1 |
| Training | 446DRE | PCA230311 | 38.04 | 11.56 | 36.22 | 9.74 | 42.91 | 16.43 | 5 | 1 | PCa | 1 | 0.7082 | 1 | 0.6513 | 1 |
| Training | 491DRE | PCA230411 | 37.62 | 13.37 | 50 | 25.75 | 35.9 | 11.65 | 2 | 1 | PCa | 1 | 0.2565 | 0 | 0.3459 | 1 |
| Training | 461DRE | PCA230406 | 38.96 | 14.63 | 37.34 | 13.01 | 50 | 25.67 | 2 | 1 | PCa | 1 | 0.5037 | 1 | 0.2969 | 0 |
| Training | 312DRE | PCA230286 | 34.69 | 12.47 | 34.74 | 12.52 | 35.01 | 12.79 | 5 | 1 | PCa | 1 | 0.6091 | 1 | 0.6409 | 1 |
| Training | 573DRE | PCA230555 | 50 | 26.47 | 50 | 26.47 | 37.23 | 13.7 | 4 | 1 | PCa | 1 | 0.0312 | 0 | 0.0763 | 0 |
| Training | 282DRE | PCA230123 | 50 | 22.56 | 41.56 | 16.14 | 40.4 | 12.87 | 3 | 1 | PCa | 1 | 0.155 | 0 | 0.2691 | 0 |
| Training | 505DRE | PCA230465 | 50 | 25.99 | 38.92 | 14.91 | 39.01 | 15 | 2 | 1 | PCa | 1 | 0.1023 | 0 | 0.183 | 0 |
| Training | 287DRE | PCA230105 | 38.67 | 11.69 | 38.91 | 16.26 | 39.03 | 10.87 | 2 | 1 | PCa | 1 | 0.5495 | 1 | 0.6231 | 1 |
| Training | 576DRE | PCA230557 | 36.83 | 13.65 | 50 | 26.82 | 37.43 | 14.25 | 3 | 0 | PCa | 1 | 0.2275 | 0 | 0.2695 | 0 |
| Training | 322DRE | PCA230260 | 37.91 | 14.38 | 39.36 | 12.56 | 37.08 | 11.86 | 3 | 1 | PCa | 1 | 0.5261 | 1 | 0.6041 | 1 |
| Training | 324DRE | PCA230287 | 50 | 26.29 | 39.42 | 12.34 | 50 | 25.36 | 1 | 1 | PCa | 1 | 0.1232 | 0 | 0.099 | 0 |
| Training | 185DRE | PCA230042 | 37.62 | 12.73 | 39.12 | 13.92 | 37.74 | 11.01 | 2 | 1 | PCa | 1 | 0.5634 | 1 | 0.6401 | 1 |
| Training | 616DRE | PCA230514 | 33.8 | 10.37 | 33.36 | 9.93 | 32.76 | 9.33 | 3 | 1 | PCa | 1 | 0.7458 | 1 | 0.7986 | 1 |
| Training | 200DRE | PCA230064 | 31.85 | 5.37 | 34.14 | 7.83 | 34.09 | 6.292 | 5 | 1 | PCa | 1 | 0.8973 | 1 | 0.9206 | 1 |
| Training | 323DRE | PCA230291 | 50 | 26.88 | 39.13 | 12.78 | 50 | 25.06 | 2 | 1 | PCa | 1 | 0.108 | 0 | 0.0916 | 0 |
| Training | 182DRE | PCA230080 | 35.87 | 11.42 | 36.35 | 11.88 | 37.94 | 10.7 | 3 | 1 | PCa | 1 | 0.6666 | 1 | 0.7212 | 1 |
| Training | 462DRE | PCA230431 | 50 | 25.85 | 50 | 25.85 | 38.67 | 14.52 | 3 | 1 | PCa | 1 | 0.0368 | 0 | 0.0806 | 0 |
| Training | 345DRE | PCA230293 | 42.56 | 16.6 | 39.6 | 13.89 | 50 | 25.38 | 2 | 1 | PCa | 1 | 0.3963 | 0 | 0.2388 | 0 |
| Training | 194DRE | PCA230030 | 34.52 | 6.41 | 36.47 | 8.48 | 36.96 | 10.96 | 3 | 1 | PCa | 1 | 0.872 | 1 | 0.8645 | 1 |
| Training | 560DRE | PCA230559 | 36.08 | 12.45 | 50 | 26.37 | 36.39 | 12.76 | 3 | 1 | PCa | 1 | 0.2757 | 0 | 0.3367 | 1 |
| Training | 507DRE | PCA230463 | 28.54 | 4.71 | 26.84 | 3.01 | 27.98 | 4.15 | 3 | 1 | PCa | 1 | 0.9413 | 1 | 0.9593 | 1 |
| Training | 514DRE | PCA230470 | 34.65 | 10.05 | 36.14 | 11.54 | 33.24 | 8.64 | 2 | 1 | PCa | 1 | 0.7247 | 1 | 0.7909 | 1 |
| Training | 192DRE | PCA230069 | 50 | 24.47 | 41.59 | 13.61 | 39.38 | 11.88 | 5 | 1 | PCa | 1 | 0.1452 | 0 | 0.2857 | 0 |
| Training | 216DRE | PCA230169 | 50 | 24.25 | 41.38 | 15.32 | 50 | 22.85 | 1 | 1 | PCa | 1 | 0.1291 | 0 | 0.1183 | 0 |
| Training | 300DRE | PCA230088 | 39.08 | 11.58 | 39.36 | 11.82 | 50 | 22.73 | 3 | 1 | PCa | 1 | 0.6618 | 1 | 0.4699 | 1 |
| Training | 218DRE | PCA230142 | 36.24 | 10.42 | 35.86 | 10 | 37.58 | 12.00 | 2 | 1 | PCa | 1 | 0.7427 | 1 | 0.7558 | 1 |
| Training | 547DRE | PCA230500 | 34.29 | 8.87 | 38.57 | 13.15 | 34.63 | 9.21 | 3 | 1 | PCa | 1 | 0.7332 | 1 | 0.7823 | 1 |
| Training | 195DRE | PCA230046 | 35.33 | 9.01 | 34.87 | 8.8 | 36.73 | 9.72 | 1 | 1 | PCa | 1 | 0.8069 | 1 | 0.8339 | 1 |
| Training | 548DRE | PCA230499 | 32.11 | 9.18 | 32.17 | 9.24 | 32.02 | 9.09 | 3 | 1 | PCa | 1 | 0.795 | 1 | 0.8331 | 1 |
| Training | 615DRE | PCA230583 | 38.4 | 13.91 | 35.97 | 11.48 | 37.92 | 13.43 | 2 | 1 | PCa | 1 | 0.5737 | 1 | 0.6084 | 1 |
| Training | 603DRE | PCA230516 | 39 | 15.98 | 50 | 26.98 | 36.47 | 13.45 | 2 | 1 | PCa | 1 | 0.1615 | 0 | 0.2265 | 0 |
| Training | 270DRE | PCA230122 | 38.4 | 12.91 | 35.08 | 7.87 | 35.17 | 9.62 | 5 | 1 | PCa | 1 | 0.6986 | 1 | 0.7734 | 1 |
| Training | 196DRE | PCA230054 | 38.05 | 9.6 | 37.71 | 9.4 | 37.58 | 9.18 | 3 | 1 | PCa | 1 | 0.7799 | 1 | 0.8225 | 1 |
| Training | 234DRE | PCA230163 | 36.18 | 8.84 | 36.91 | 9.69 | 37.54 | 10.90 | 4 | 1 | PCa | 1 | 0.7972 | 1 | 0.8099 | 1 |
| Training | 586DRE | PCA230533 | 34.01 | 11.66 | 33.72 | 11.37 | 32.78 | 10.43 | 2 | 1 | PCa | 1 | 0.6688 | 1 | 0.7293 | 1 |
| Training | 538DRE | PCA230479 | 50 | 26.04 | 38.48 | 14.52 | 50 | 26.04 | 5 | 1 | PCa | 1 | 0.1052 | 0 | 0.0805 | 0 |
| Training | 423DRE | PCA230313 | 36.15 | 10.72 | 28.19 | 2.76 | 29.33 | 3.9 | 4 | 1 | PCa | 1 | 0.8515 | 1 | 0.9216 | 1 |
| Training | 484DRE | PCA230435 | 36.15 | 10.4 | 33.58 | 7.83 | 36.22 | 10.47 | 4 | 1 | PCa | 1 | 0.7833 | 1 | 0.8123 | 1 |
| Training | 571DRE | PCA230541 | 50 | 26.84 | 37.29 | 14.13 | 46.36 | 23.2 | 4 | 1 | PCa | 1 | 0.0961 | 0 | 0.0954 | 0 |
| Training | 421DRE | PCA230318 | 31.14 | 6.08 | 31.49 | 6.43 | 32.59 | 7.53 | 4 | 1 | PCa | 1 | 0.899 | 1 | 0.9155 | 1 |
| Training | 313DRE | PCA230289 | 38.4 | 11.41 | 37.85 | 10.87 | 37.37 | 12.63 | 5 | 1 | PCa | 1 | 0.6895 | 1 | 0.7056 | 1 |
| Training | 541DRE | PCA230454 | 50 | 26.45 | 50 | 26.45 | 50 | 26.45 | 2 | 1 | PCa | 1 | 0.0314 | 0 | 0.0263 | 0 |
| Training | 197DRE | PCA230060 | 36.55 | 11.03 | 39.67 | 13.22 | 37.98 | 11.51 | 3 | 1 | PCa | 1 | 0.6513 | 1 | 0.691 | 1 |
| Training | 227DRE | PCA230160 | 35.26 | 7.7 | 36.65 | 8.75 | 35.85 | 7.87 | 3 | 1 | PCa | 1 | 0.8409 | 1 | 0.8745 | 1 |
| Training | 559DRE | PCA230560 | 30.75 | 6.94 | 34.25 | 10.44 | 30.91 | 7.1 | 5 | 1 | PCa | 1 | 0.8356 | 1 | 0.8753 | 1 |
| Training | 328DRE | PCA230272 | 50 | 23.97 | 40.27 | 14.28 | 38.59 | 12.527 | 2 | 1 | PCa | 1 | 0.1476 | 0 | 0.2743 | 0 |
| Training | 329DRE | PCA230268 | 38.56 | 10.63 | 39.21 | 11.64 | 50 | 23.307 | 3 | 1 | PCa | 1 | 0.7019 | 1 | 0.4906 | 1 |
| Training | 561DRE | PCA230598 | 28.86 | 4.84 | 27 | 2.98 | 27.99 | 3.97 | 5 | 1 | PCa | 1 | 0.9402 | 1 | 0.9594 | 1 |
| Training | 332DRE | PCA230299 | 32.4 | 6.25 | 40.67 | 14.69 | 31.72 | 5.87 | 5 | 1 | PCa | 1 | 0.7881 | 1 | 0.8525 | 1 |
| Training | 453DRE | PCA230437 | 34.82 | 10.98 | 35.22 | 11.38 | 37.61 | 13.77 | 5 | 1 | PCa | 1 | 0.6945 | 1 | 0.6858 | 1 |
| Training | 456DRE | PCA230449 | 37.56 | 13.97 | 36.23 | 12.64 | 38.6 | 15.01 | 4 | 1 | PCa | 1 | 0.5419 | 1 | 0.5471 | 1 |
| Training | 613DRE | PCA230577 | 30.76 | 5.39 | 30.43 | 5.06 | 29.6 | 4.23 | 3 | 1 | PCa | 1 | 0.9203 | 1 | 0.9469 | 1 |
| Training | 431DRE | PCA230345 | 37.78 | 10.33 | 35.32 | 7.87 | 36.96 | 9.51 | 4 | 1 | PCa | 1 | 0.7847 | 1 | 0.8255 | 1 |
| Training | 186DRE | PCA230051 | 33.9 | 7.27 | 38.72 | 9.49 | 39.49 | 9.60 | 4 | 1 | PCa | 1 | 0.8409 | 1 | 0.8552 | 1 |
| Training | 596DRE | PCA230585 | 36.08 | 12.53 | 36.56 | 13.01 | 35.19 | 11.64 | 5 | 1 | PCa | 1 | 0.5946 | 1 | 0.6521 | 1 |
| Training | 178DRE | PCA230081 | 38.59 | 11.81 | 44.88 | 16.44 | 38.52 | 11.03 | 3 | 1 | PCa | 1 | 0.5398 | 1 | 0.6125 | 1 |
| Training | 190DRE | PCA230070 | 35.82 | 9 | 37.97 | 9.35 | 37.26 | 8.855 | 3 | 1 | PCa | 1 | 0.7983 | 1 | 0.8376 | 1 |
| Training | 306DRE | PCA230083 | 50 | 24.68 | 50 | 23.95 | 50 | 23.52 | 2 | 1 | PCa | 1 | 0.0539 | 0 | 0.0518 | 0 |
| Training | 318DRE | PCA230279 | 50 | 23.2 | 39.36 | 12.45 | 50 | 23.01 | 2 | 1 | PCa | 1 | 0.1928 | 0 | 0.1636 | 0 |
| Training | 202DRE | PCA230055 | 37.89 | 9.72 | 39.95 | 11.39 | 40.93 | 12.23 | 5 | 1 | PCa | 1 | 0.7391 | 1 | 0.7445 | 1 |
| Training | 492DRE | PCA230419 | 38.24 | 15.04 | 35.08 | 11.88 | 37.31 | 14.11 | 5 | 1 | PCa | 1 | 0.5145 | 1 | 0.5512 | 1 |
| Training | 429DRE | PCA230377 | 28.66 | 4.26 | 28.08 | 3.68 | 29.04 | 4.64 | 5 | 1 | PCa | 1 | 0.9419 | 1 | 0.9573 | 1 |
| Training | 435DRE | PCA230325 | 34.09 | 9.34 | 32.62 | 7.87 | 36.38 | 11.63 | 5 | 1 | PCa | 1 | 0.8126 | 1 | 0.8161 | 1 |
| Training | 235DRE | PCA230151 | 50 | 24.51 | 40.3 | 12.67 | 39.86 | 13.88 | 5 | 1 | PCa | 1 | 0.1566 | 0 | 0.2667 | 0 |
| Training | 476DRE | PCA230444 | 50 | 23.46 | 50 | 23.46 | 50 | 23.46 | 5 | 1 | PCa | 1 | 0.0691 | 0 | 0.0626 | 0 |
| Training | 510DRE | PCA230485 | 32.64 | 7.95 | 31.54 | 6.85 | 32.63 | 7.94 | 5 | 1 | PCa | 1 | 0.86 | 1 | 0.8887 | 1 |
| Training | 269DRE | PCA230104 | 29.57 | 3.33 | 30.44 | 5.96 | 30.98 | 6.257 | 4 | 1 | PCa | 1 | 0.938 | 1 | 0.9466 | 1 |
| Training | 219DRE | PCA230143 | 50 | 22.42 | 39.54 | 12.12 | 39.77 | 12.14 | 5 | 1 | PCa | 1 | 0.2208 | 0 | 0.3656 | 1 |
| Training | 198DRE | PCA230066 | 34.27 | 6.79 | 35.64 | 8.3 | 37 | 9.27 | 4 | 1 | PCa | 1 | 0.8665 | 1 | 0.8778 | 1 |
| Training | 511DRE | PCA230487 | 33.8 | 11.37 | 36.08 | 13.65 | 35.32 | 12.89 | 2 | 1 | PCa | 1 | 0.6274 | 1 | 0.6463 | 1 |
| Training | 326DRE | PCA230257 | 33.68 | 7.57 | 34.41 | 7.72 | 34.19 | 8.19 | 5 | 1 | PCa | 1 | 0.8573 | 1 | 0.8832 | 1 |
| Training | 290DRE | PCA230118 | 31.09 | 4.12 | 32.93 | 4.92 | 33.1 | 4.42 | 5 | 1 | PCa | 1 | 0.9361 | 1 | 0.9541 | 1 |
| Training | 191DRE | PCA230035 | 36.12 | 11.27 | 33.24 | 9.19 | 36.55 | 11.27 | 4 | 1 | PCa | 1 | 0.7298 | 1 | 0.762 | 1 |
| Training | 314DRE | PCA230290 | 33.74 | 8.11 | 36.51 | 8.15 | 35.53 | 6.723 | 4 | 1 | PCa | 1 | 0.8395 | 1 | 0.8856 | 1 |
| Training | 331DRE | PCA230252 | 50 | 26.78 | 39.99 | 15.56 | 50 | 26.80 | 4 | 1 | PCa | 1 | 0.085 | 0 | 0.0637 | 0 |
| Training | 340DRE | PCA230270 | 35.64 | 9.61 | 37.08 | 12.97 | 37.47 | 10.99 | 5 | 1 | PCa | 1 | 0.7108 | 1 | 0.7404 | 1 |
| Training | 425DRE | PCA230343 | 50 | 21.49 | 39.28 | 10.77 | 50 | 21.49 | 4 | 1 | PCa | 1 | 0.2768 | 0 | 0.2432 | 0 |
| Training | 439DRE | PCA230320 | 30.21 | 5.98 | 29.81 | 5.58 | 31.43 | 7.2 | 5 | 1 | PCa | 1 | 0.9081 | 1 | 0.9242 | 1 |
| Training | 442DRE | PCA230322 | 35.79 | 9.76 | 34.04 | 8.01 | 36.34 | 10.31 | 4 | 1 | PCa | 1 | 0.7988 | 1 | 0.8237 | 1 |
| Training | 443DRE | PCA230303 | 26.78 | 1.28 | 25.71 | 0.21 | 27.21 | 1.71 | 4 | 1 | PCa | 1 | 0.975 | 1 | 0.9829 | 1 |
| Training | 444DRE | PCA230302 | 29.06 | 7.21 | 28.62 | 6.77 | 30.01 | 8.16 | 4 | 1 | PCa | 1 | 0.8758 | 1 | 0.8963 | 1 |
| Training | 445DRE | PCA230329 | 26.43 | 2.71 | 26.23 | 2.51 | 26.84 | 3.12 | 2 | 1 | PCa | 1 | 0.96 | 1 | 0.9718 | 1 |
| Training | 454DRE | PCA230440 | 27.63 | 2.66 | 28.07 | 3.1 | 28.22 | 3.25 | 5 | 1 | PCa | 1 | 0.9579 | 1 | 0.9702 | 1 |
| Training | 468DRE | PCA230446 | 24.91 | 0.87 | 26.31 | 2.27 | 25.62 | 1.58 | 5 | 1 | PCa | 1 | 0.9714 | 1 | 0.9806 | 1 |
| Training | 475DRE | PCA230421 | 33.38 | 7.01 | 32.99 | 6.62 | 34.06 | 7.69 | 4 | 1 | PCa | 1 | 0.8811 | 1 | 0.9035 | 1 |
| Training | 488DRE | PCA230427 | 36.48 | 12.98 | 34.13 | 10.63 | 36.05 | 12.55 | 4 | 1 | PCa | 1 | 0.6334 | 1 | 0.6703 | 1 |
| Training | 489DRE | PCA230420 | 29.82 | 3.82 | 29.63 | 3.63 | 28.68 | 2.68 | 5 | 1 | PCa | 1 | 0.9462 | 1 | 0.9658 | 1 |
| Training | 490DRE | PCA230447 | 32.95 | 9.44 | 31.33 | 7.82 | 34.82 | 11.31 | 5 | 1 | PCa | 1 | 0.8107 | 1 | 0.8191 | 1 |
| Training | 528DRE | PCA230496 | 30.11 | 6.42 | 31.18 | 7.49 | 29.93 | 6.24 | 4 | 1 | PCa | 1 | 0.8827 | 1 | 0.9135 | 1 |
| Training | 551DRE | PCA230593 | 50 | 26.56 | 33.73 | 10.29 | 35.78 | 12.34 | 5 | 1 | PCa | 1 | 0.1418 | 0 | 0.2867 | 0 |
| Training | 553DRE | PCA230526 | 34.03 | 10.38 | 32.58 | 8.93 | 33.79 | 10.14 | 5 | 1 | PCa | 1 | 0.7643 | 1 | 0.8016 | 1 |
| Training | 554DRE | PCA230596 | 38.35 | 13.85 | 39.75 | 15.25 | 37.74 | 13.24 | 5 | 1 | PCa | 1 | 0.4808 | 1 | 0.5302 | 1 |
| Training | 556DRE | PCA230599 | 31.08 | 7.58 | 29.1 | 5.6 | 33.02 | 9.52 | 5 | 1 | PCa | 1 | 0.8816 | 1 | 0.8908 | 1 |
| Training | 558DRE | PCA230594 | 32.86 | 9.78 | 33.56 | 10.48 | 34.78 | 11.7 | 5 | 1 | PCa | 1 | 0.7547 | 1 | 0.7671 | 1 |
| Training | 582DRE | PCA230547 | 29.12 | 6.28 | 28.45 | 5.61 | 29.21 | 6.37 | 4 | 1 | PCa | 1 | 0.9033 | 1 | 0.9265 | 1 |
| Training | 201DRE | PCA230063 | 33.54 | 6.08 | 39.73 | 11.9 | 36.26 | 7.51 | 3 | 1 | PCa | 1 | 0.8359 | 1 | 0.8683 | 1 |
| Training | 204DRE | PCA230057 | 50 | 23.12 | 39.41 | 12.26 | 41.19 | 12.73 | 4 | 1 | PCa | 1 | 0.1981 | 0 | 0.3313 | 1 |
| Training | 239DRE | PCA230156 | 50 | 22.47 | 36.01 | 8.15 | 36.37 | 8.84 | 5 | 1 | PCa | 1 | 0.2963 | 0 | 0.5236 | 1 |
| Training | 540DRE | PCA230461 | 29.09 | 3.88 | 29.74 | 4.53 | 29.26 | 4.05 | 4 | 1 | PCa | 1 | 0.9408 | 1 | 0.9582 | 1 |
| Training | 254DRE | PCA230130 | 37.58 | 10.85 | 39.38 | 12.37 | 38.79 | 15.20 | 5 | 1 | PCa | 1 | 0.6777 | 1 | 0.6412 | 1 |
| validation | 650DRE | PCA230667 | 50 | 26.75 | 35.14 | 11.89 | 50 | 26.75 | 0 | 0 | BPH | 0 | 0.1195 | 0 | 0.0874 | 0 |
| validation | 781DRE | PCA231198 | 50 | 25.3 | 39.17 | 14.47 | 40.21 | 15.51 | 0 | 1 | BPH | 0 | 0.1186 | 0 | 0.1953 | 0 |
| validation | 759DRE | PCA231193 | 50 | 27.15 | 50 | 27.15 | 50 | 27.15 | 0 | 1 | BPH | 0 | 0.026 | 0 | 0.0214 | 0 |
| validation | 768DRE | PCA231199 | 50 | 23.45 | 50 | 23.45 | 50 | 23.45 | 0 | 1 | BPH | 0 | 0.0693 | 0 | 0.0628 | 0 |
| validation | 729DRE | PCA230646 | 38.3 | 14.09 | 37.02 | 12.81 | 50 | 25.79 | 0 | 1 | BPH | 0 | 0.5324 | 1 | 0.3126 | 0 |
| validation | 657DRE | PCA230669 | 50 | 27.79 | 50 | 27.79 | 34.04 | 11.83 | 0 | 0 | BPH | 0 | 0.0218 | 0 | 0.0683 | 0 |
| validation | 715DRE | PCA230618 | 37.12 | 11.14 | 36.78 | 10.8 | 36.16 | 10.18 | 0 | 1 | BPH | 0 | 0.7011 | 1 | 0.7556 | 1 |
| validation | 635DRE | PCA230659 | 36.55 | 13.14 | 33.37 | 9.96 | 35.72 | 12.31 | 0 | 0 | BPH | 0 | 0.6427 | 1 | 0.684 | 1 |
| validation | 661DRE | PCA230689 | 45.44 | 22.3 | 50 | 26.86 | 50 | 26.86 | 0 | 0 | OUM | 0 | 0.0604 | 0 | 0.0402 | 0 |
| validation | 627DRE | PCA230692 | 37.32 | 17.09 | 36.43 | 16.2 | 32.88 | 12.65 | 0 | 0 | OUM | 0 | 0.3225 | 0 | 0.4226 | 1 |
| validation | 666DRE | PCA230675 | 36.88 | 12.6 | 36.16 | 11.88 | 35.63 | 11.35 | 0 | 0 | BPH | 0 | 0.6192 | 1 | 0.6787 | 1 |
| validation | 840DRE | PCA231246 | 50 | 27.17 | 38.7 | 15.87 | 50 | 27.17 | 0 | 0 | BPH | 0 | 0.0775 | 0 | 0.0575 | 0 |
| validation | 822DRE | PCA231241 | 50 | 27.6 | 36.93 | 14.53 | 37.96 | 15.56 | 0 | 1 | BPH | 0 | 0.082 | 0 | 0.1533 | 0 |
| validation | 763DRE | PCA231174 | 36.36 | 10.91 | 35.87 | 10.42 | 37.73 | 12.28 | 0 | 1 | BPH | 0 | 0.7174 | 1 | 0.7325 | 1 |
| validation | 847DRE | PCA231238 | 50 | 27.34 | 37.14 | 14.48 | 37.23 | 14.57 | 0 | 0 | BPH | 0 | 0.0859 | 0 | 0.1701 | 0 |
| validation | 754DRE | PCA231187 | 50 | 27 | 39.84 | 16.84 | 35.56 | 12.56 | 0 | 1 | BPH | 0 | 0.0727 | 0 | 0.1705 | 0 |
| validation | 795DRE | PCA231190 | 50 | 26.8 | 37.84 | 14.64 | 50 | 26.8 | 0 | 1 | BPH | 0 | 0.0922 | 0 | 0.0687 | 0 |
| validation | 691DRE | PCA230632 | 50 | 26.84 | 35.64 | 12.48 | 35.65 | 12.49 | 0 | 1 | BPH | 0 | 0.1117 | 0 | 0.2389 | 0 |
| validation | 699DRE | PCA230633 | 50 | 26.77 | 50 | 26.77 | 37.2 | 13.97 | 0 | 1 | OUM | 0 | 0.0288 | 0 | 0.0703 | 0 |
| validation | 626DRE | PCA230691 | 50 | 26.33 | 36.23 | 12.56 | 39.57 | 15.9 | 0 | 0 | BPH | 0 | 0.1201 | 0 | 0.1974 | 0 |
| validation | 701DRE | PCA231140 | 50 | 26.49 | 38.21 | 14.7 | 50 | 26.49 | 0 | 0 | BPH | 0 | 0.0964 | 0 | 0.0726 | 0 |
| validation | 755DRE | PCA231188 | 37.49 | 13.29 | 35.46 | 11.26 | 37.33 | 13.13 | 0 | 1 | BPH | 0 | 0.6055 | 1 | 0.6372 | 1 |
| validation | 705DRE | PCA231152 | 50 | 27.67 | 44.43 | 22.1 | 50 | 27.67 | 0 | 0 | BPH | 0 | 0.0392 | 0 | 0.0302 | 0 |
| validation | 625DRE | PCA230615 | 50 | 24 | 50 | 24 | 40.14 | 14.14 | 0 | 0 | BPH | 0 | 0.0601 | 0 | 0.1188 | 0 |
| validation | 641DRE | PCA230684 | 37.33 | 14.61 | 40.83 | 18.11 | 50 | 27.28 | 0 | 0 | BPH | 0 | 0.377 | 0 | 0.1876 | 0 |
| validation | 655DRE | PCA230642 | 35.61 | 11.37 | 35.22 | 10.98 | 34.55 | 10.31 | 0 | 1 | BPH | 0 | 0.6886 | 1 | 0.7451 | 1 |
| validation | 623DRE | PCA230698 | 50 | 27.66 | 41.46 | 19.12 | 36.63 | 14.29 | 0 | 0 | OUM | 0 | 0.0524 | 0 | 0.1168 | 0 |
| validation | 798DRE | PCA231205 | 50 | 27.17 | 40.44 | 17.61 | 50 | 27.17 | 0 | 1 | BPH | 0 | 0.0657 | 0 | 0.0495 | 0 |
| validation | 810DRE | PCA231240 | 38.27 | 11.93 | 50 | 23.66 | 44.47 | 18.13 | 0 | 1 | BPH | 0 | 0.3548 | 0 | 0.3019 | 0 |
| validation | 726DRE | PCA230612 | 37.76 | 12.35 | 34.43 | 9.02 | 37.83 | 12.42 | 0 | 1 | BPH | 0 | 0.6946 | 1 | 0.7202 | 1 |
| validation | 716DRE | PCA230636 | 50 | 26.83 | 50 | 26.83 | 50 | 26.83 | 0 | 1 | BPH | 0 | 0.0283 | 0 | 0.0236 | 0 |
| validation | 752DRE | PCA231182 | 50 | 27.17 | 35.45 | 12.62 | 50 | 27.17 | 0 | 1 | BPH | 0 | 0.1048 | 0 | 0.0758 | 0 |
| validation | 772DRE | PCA231207 | 50 | 27.37 | 50 | 27.37 | 50 | 27.37 | 0 | 1 | BPH | 0 | 0.0245 | 0 | 0.0201 | 0 |
| validation | 654DRE | PCA230640 | 50 | 24.73 | 50 | 24.73 | 37.45 | 12.18 | 0 | 1 | BPH | 0 | 0.0496 | 0 | 0.1204 | 0 |
| validation | 630DRE | PCA230610 | 50 | 26.44 | 42.37 | 18.81 | 37.22 | 13.66 | 0 | 0 | OUM | 0 | 0.0661 | 0 | 0.1432 | 0 |
| validation | 717DRE | PCA230645 | 45.38 | 21.95 | 34.6 | 11.17 | 37.07 | 13.64 | 0 | 1 | BPH | 0 | 0.2532 | 0 | 0.3685 | 1 |
| validation | 773DRE | PCA231119 | 50 | 23.66 | 37.17 | 10.83 | 50 | 23.66 | 0 | 0 | BPH | 0 | 0.2064 | 0 | 0.1683 | 0 |
| validation | 651DRE | PCA230674 | 50 | 27.13 | 36.05 | 13.18 | 36.51 | 13.64 | 0 | 0 | BPH | 0 | 0.1002 | 0 | 0.2044 | 0 |
| validation | 631DRE | PCA230616 | 46.56 | 23.97 | 35.16 | 12.57 | 50 | 27.41 | 0 | 0 | BPH | 0 | 0.1709 | 0 | 0.1068 | 0 |
| validation | 624DRE | PCA230699 | 50 | 24.94 | 41.14 | 16.08 | 50 | 24.94 | 0 | 0 | BPH | 0 | 0.1084 | 0 | 0.0874 | 0 |
| validation | 647DRE | PCA230683 | 37.89 | 10.69 | 50 | 22.8 | 50 | 22.8 | 0 | 0 | BPH | 0 | 0.4273 | 0 | 0.2655 | 0 |
| validation | 648DRE | PCA230678 | 50 | 26.86 | 50 | 26.86 | 50 | 26.86 | 0 | 0 | BPH | 0 | 0.0281 | 0 | 0.0234 | 0 |
| validation | 837DRE | PCA231235 | 50 | 25.21 | 40.12 | 15.33 | 50 | 25.21 | 0 | 0 | BPH | 0 | 0.1113 | 0 | 0.0883 | 0 |
| validation | 764DRE | PCA231180 | 50 | 27.36 | 50 | 27.36 | 42.55 | 19.91 | 0 | 1 | BPH | 0 | 0.0245 | 0 | 0.038 | 0 |
| validation | 846DRE | PCA231217 | 50 | 27.62 | 50 | 27.62 | 50 | 27.62 | 0 | 0 | BPH | 0 | 0.0229 | 0 | 0.0187 | 0 |
| validation | 652DRE | PCA230607 | 50 | 23.83 | 50 | 23.83 | 50 | 23.83 | 0 | 1 | BPH | 0 | 0.0628 | 0 | 0.0563 | 0 |
| validation | 841DRE | PCA231249 | 50 | 26.74 | 33.53 | 10.27 | 50 | 26.74 | 0 | 0 | BPH | 0 | 0.1382 | 0 | 0.1001 | 0 |
| validation | 757DRE | PCA231191 | 41.44 | 17.19 | 36.26 | 12.01 | 37.83 | 13.58 | 0 | 1 | BPH | 0 | 0.4177 | 0 | 0.4939 | 1 |
| validation | 756DRE | PCA231189 | 50 | 23.08 | 50 | 23.08 | 38.41 | 11.49 | 0 | 1 | BPH | 0 | 0.0762 | 0 | 0.1716 | 0 |
| validation | 618DRE | PCA230512 | 39.65 | 17.4 | 35.44 | 13.19 | 39.19 | 16.94 | 0 | 0 | BPH | 0 | 0.38 | 0 | 0.3887 | 1 |
| validation | 719DRE | PCA231115 | 50 | 26.46 | 34.63 | 11.09 | 50 | 26.46 | 0 | 0 | BPH | 0 | 0.1341 | 0 | 0.0987 | 0 |
| validation | 633DRE | PCA230644 | 50 | 27.4 | 50 | 27.4 | 37.29 | 14.69 | 0 | 0 | BPH | 0 | 0.0243 | 0 | 0.0584 | 0 |
| validation | 622DRE | PCA230697 | 50 | 27.46 | 36.04 | 13.5 | 50 | 27.46 | 0 | 0 | BPH | 0 | 0.0923 | 0 | 0.0665 | 0 |
| validation | 697DRE | PCA230628 | 50 | 27.06 | 50 | 27.06 | 40.1 | 17.16 | 0 | 1 | BPH | 0 | 0.0266 | 0 | 0.051 | 0 |
| validation | 634DRE | PCA230641 | 50 | 26.91 | 50 | 26.91 | 50 | 26.91 | 0 | 0 | OUM | 0 | 0.0277 | 0 | 0.023 | 0 |
| validation | 791DRE | PCA231173 | 50 | 24.62 | 50 | 24.62 | 36.88 | 11.5 | 0 | 1 | BPH | 0 | 0.0511 | 0 | 0.1295 | 0 |
| validation | 758DRE | PCA231192 | 50 | 27.04 | 36.96 | 14 | 50 | 27.04 | 0 | 1 | BPH | 0 | 0.0942 | 0 | 0.0692 | 0 |
| validation | 619DRE | PCA230581 | 50 | 27.18 | 34.84 | 12.02 | 50 | 27.18 | 0 | 0 | BPH | 0 | 0.1105 | 0 | 0.0795 | 0 |
| validation | 765DRE | PCA231186 | 50 | 26.75 | 39.3 | 16.05 | 41.23 | 17.98 | 0 | 1 | BPH | 0 | 0.0815 | 0 | 0.124 | 0 |
| validation | 632DRE | PCA230639 | 34.48 | 10.21 | 35.52 | 11.25 | 35.43 | 11.16 | 0 | 1 | BPH | 0 | 0.725 | 1 | 0.7533 | 1 |
| validation | 737DRE | PCA230604 | 50 | 25.17 | 48.79 | 23.96 | 37.85 | 13.02 | 0 | 1 | BPH | 0 | 0.0497 | 0 | 0.1145 | 0 |
| validation | 808DRE | PCA231215 | 50 | 26.87 | 50 | 26.87 | 50 | 26.87 | 0 | 1 | BPH | 0 | 0.028 | 0 | 0.0233 | 0 |
| validation | 790DRE | PCA231171 | 50 | 22.93 | 50 | 22.93 | 50 | 22.93 | 0 | 1 | BPH | 0 | 0.0792 | 0 | 0.0726 | 0 |
| validation | 636DRE | PCA230662 | 37.95 | 13.47 | 40.72 | 16.24 | 50 | 25.52 | 0 | 1 | BPH | 0 | 0.4722 | 1 | 0.2688 | 0 |
| validation | 805DRE | PCA231147 | 38.34 | 14.9 | 39.51 | 16.07 | 37.85 | 14.41 | 0 | 1 | BPH | 0 | 0.4146 | 0 | 0.4538 | 1 |
| validation | 817DRE | PCA231253 | 50 | 23.63 | 50 | 23.63 | 50 | 23.63 | 0 | 1 | BPH | 0 | 0.0661 | 0 | 0.0596 | 0 |
| validation | 820DRE | PCA231276 | 50 | 25.98 | 38.01 | 13.99 | 50 | 25.98 | 0 | 1 | BPH | 0 | 0.1114 | 0 | 0.0851 | 0 |
| validation | 685DRE | PCA231144 | 50 | 26.91 | 50 | 26.91 | 50 | 26.91 | 0 | 1 | BPH | 0 | 0.0277 | 0 | 0.023 | 0 |
| validation | 788DRE | PCA231168 | 50 | 23.84 | 50 | 23.84 | 50 | 23.84 | 0 | 1 | BPH | 0 | 0.0626 | 0 | 0.0562 | 0 |
| validation | 696DRE | PCA230619 | 50 | 26.97 | 50 | 26.97 | 50 | 26.97 | 0 | 1 | BPH | 0 | 0.0273 | 0 | 0.0226 | 0 |
| validation | 767DRE | PCA231196 | 50 | 27.06 | 37.1 | 14.16 | 37.72 | 14.78 | 0 | 1 | BPH | 0 | 0.0925 | 0 | 0.1765 | 0 |
| validation | 762DRE | PCA231099 | 35.72 | 12.18 | 50 | 26.46 | 35.58 | 12.04 | 0 | 1 | BPH | 0 | 0.2834 | 0 | 0.3567 | 1 |
| validation | 834DRE | PCA231264 | 50 | 26.82 | 50 | 26.82 | 50 | 26.82 | 0 | 1 | BPH | 0 | 0.0284 | 0 | 0.0236 | 0 |
| validation | 813DRE | PCA231130 | 50 | 24.55 | 50 | 24.55 | 50 | 24.55 | 0 | 1 | BPH | 0 | 0.052 | 0 | 0.0458 | 0 |
| validation | 774DRE | PCA231135 | 50 | 27.45 | 44.48 | 21.93 | 50 | 27.45 | 0 | 1 | BPH | 0 | 0.0413 | 0 | 0.032 | 0 |
| validation | 783DRE | PCA231116 | 50 | 26.94 | 37.36 | 14.3 | 50 | 26.94 | 0 | 1 | BPH | 0 | 0.0931 | 0 | 0.0688 | 0 |
| validation | 787DRE | PCA231167 | 50 | 26.6 | 36.29 | 12.89 | 50 | 26.6 | 0 | 1 | BPH | 0 | 0.1118 | 0 | 0.0827 | 0 |
| validation | 672DRE | PCA230638 | 50 | 25.34 | 49.34 | 24.68 | 37.22 | 12.56 | 0 | 1 | BPH | 0 | 0.045 | 0 | 0.1099 | 0 |
| validation | 639DRE | PCA230672 | 34.43 | 10.98 | 37.76 | 14.31 | 34.39 | 10.94 | 0 | 1 | BPH | 0 | 0.6277 | 1 | 0.6818 | 1 |
| validation | 723DRE | PCA231143 | 50 | 27.09 | 37.09 | 14.18 | 50 | 27.09 | 0 | 1 | BPH | 0 | 0.0919 | 0 | 0.0675 | 0 |
| validation | 653DRE | PCA230609 | 50 | 27.34 | 50 | 27.34 | 50 | 27.34 | 0 | 1 | BPH | 0 | 0.0247 | 0 | 0.0203 | 0 |
| validation | 793DRE | PCA231181 | 50 | 26.69 | 46.58 | 23.27 | 50 | 26.69 | 0 | 1 | BPH | 0 | 0.0412 | 0 | 0.0332 | 0 |
| validation | 831DRE | PCA231251 | 50 | 27.06 | 37.25 | 14.31 | 50 | 27.06 | 0 | 1 | BPH | 0 | 0.0912 | 0 | 0.0671 | 0 |
| validation | 779DRE | PCA231177 | 50 | 27.75 | 37.31 | 15.06 | 50 | 27.75 | 0 | 1 | BPH | 0 | 0.0761 | 0 | 0.0549 | 0 |
| validation | 688DRE | PCA231160 | 50 | 27.18 | 37.36 | 14.54 | 50 | 27.18 | 0 | 1 | BPH | 0 | 0.0876 | 0 | 0.0643 | 0 |
| validation | 658DRE | PCA230671 | 50 | 26.71 | 35.15 | 11.86 | 50 | 26.71 | 0 | 1 | BPH | 0 | 0.1206 | 0 | 0.0883 | 0 |
| validation | 694DRE | PCA230603 | 50 | 26.86 | 50 | 26.86 | 37.34 | 14.2 | 0 | 1 | BPH | 0 | 0.0281 | 0 | 0.0678 | 0 |
| validation | 727DRE | PCA230614 | 39.49 | 16.31 | 37.44 | 14.26 | 50 | 26.82 | 0 | 1 | BPH | 0 | 0.3995 | 0 | 0.2169 | 0 |
| validation | 656DRE | PCA230652 | 38.75 | 13.39 | 50 | 24.64 | 50 | 24.64 | 0 | 1 | BPH | 0 | 0.278 | 0 | 0.1572 | 0 |
| validation | 733DRE | PCA231085 | 50 | 27.21 | 38.41 | 15.62 | 50 | 27.21 | 0 | 1 | BPH | 0 | 0.0788 | 0 | 0.0583 | 0 |
| validation | 718DRE | PCA230647 | 50 | 26.85 | 50 | 26.85 | 50 | 26.85 | 0 | 1 | BPH | 0 | 0.0282 | 0 | 0.0234 | 0 |
| validation | 714DRE | PCA230611 | 50 | 25.14 | 50 | 25.14 | 50 | 25.14 | 0 | 1 | BPH | 0 | 0.0445 | 0 | 0.0386 | 0 |
| validation | 845DRE | PCA231269 | 50 | 26.08 | 36.32 | 12.4 | 50 | 26.08 | 0 | 1 | BPH | 0 | 0.1266 | 0 | 0.0952 | 0 |
| validation | 816DRE | PCA231252 | 38.5 | 12.8 | 50 | 24.3 | 50 | 24.3 | 0 | 1 | BPH | 0 | 0.3066 | 0 | 0.1757 | 0 |
| validation | 669DRE | PCA230605 | 50 | 26.76 | 44.97 | 21.73 | 50 | 26.76 | 0 | 1 | BPH | 0 | 0.0473 | 0 | 0.0375 | 0 |
| validation | 629DRE | PCA230696 | 50 | 27.49 | 50 | 27.49 | 50 | 27.49 | 0 | 1 | BPH | 0 | 0.0237 | 0 | 0.0194 | 0 |
| validation | 833DRE | PCA231261 | 35.31 | 12.14 | 32.58 | 9.41 | 35.21 | 12.04 | 0 | 1 | BPH | 0 | 0.694 | 1 | 0.7249 | 1 |
| validation | 739DRE | PCA230637 | 50 | 26.86 | 39.27 | 16.13 | 50 | 26.86 | 0 | 1 | BPH | 0 | 0.0795 | 0 | 0.0598 | 0 |
| validation | 776DRE | PCA231170 | 39.81 | 17.17 | 36.71 | 14.07 | 50 | 27.36 | 0 | 1 | BPH | 0 | 0.3684 | 0 | 0.1947 | 0 |
| validation | 809DRE | PCA231221 | 50 | 26.98 | 50 | 26.98 | 38.45 | 15.43 | 0 | 1 | BPH | 0 | 0.0272 | 0 | 0.0598 | 0 |
| validation | 838DRE | PCA231237 | 36.63 | 10.13 | 50 | 23.5 | 36.09 | 9.59 | 0 | 1 | BPH | 0 | 0.4339 | 0 | 0.5368 | 1 |
| validation | 819DRE | PCA231275 | 50 | 26.95 | 36.8 | 13.75 | 50 | 26.95 | 0 | 1 | BPH | 0 | 0.0978 | 0 | 0.0719 | 0 |
| validation | 664DRE | PCA230670 | 50 | 26.5 | 39.45 | 15.95 | 50 | 26.5 | 0 | 1 | BPH | 0 | 0.0857 | 0 | 0.0652 | 0 |
| validation | 689DRE | PCA230613 | 50 | 26.98 | 45.56 | 22.54 | 50 | 26.98 | 0 | 1 | BPH | 0 | 0.0421 | 0 | 0.0334 | 0 |
| validation | 698DRE | PCA230630 | 50 | 26.72 | 36.85 | 13.57 | 40.27 | 16.99 | 0 | 1 | BPH | 0 | 0.1031 | 0 | 0.1627 | 0 |
| validation | 839DRE | PCA231244 | 50 | 23.9 | 41.18 | 15.08 | 38.06 | 11.96 | 0 | 1 | BPH | 0 | 0.1391 | 0 | 0.2714 | 0 |
| validation | 637DRE | PCA230665 | 35.25 | 11.96 | 34.52 | 11.23 | 34.42 | 11.13 | 0 | 0 | BPH | 0 | 0.6603 | 1 | 0.712 | 1 |
| validation | 722DRE | PCA231133 | 50 | 25.66 | 50 | 25.66 | 50 | 25.66 | 0 | 1 | BPH | 0 | 0.0388 | 0 | 0.0332 | 0 |
| validation | 818DRE | PCA231258 | 50 | 24.57 | 50 | 24.57 | 50 | 24.57 | 0 | 1 | BPH | 0 | 0.0517 | 0 | 0.0456 | 0 |
| validation | 674DRE | PCA230650 | 50 | 26.81 | 37.01 | 13.82 | 38.49 | 15.3 | 0 | 1 | BPH | 0 | 0.0993 | 0 | 0.1789 | 0 |
| validation | 732DRE | PCA231083 | 50 | 27.18 | 39.7 | 16.88 | 50 | 27.18 | 0 | 1 | BPH | 0 | 0.0703 | 0 | 0.0526 | 0 |
| validation | 740DRE | PCA231127 | 50 | 23.77 | 50 | 23.77 | 50 | 23.77 | 0 | 1 | BPH | 0 | 0.0638 | 0 | 0.0573 | 0 |
| validation | 675DRE | PCA230660 | 50 | 27.21 | 50 | 27.21 | 50 | 27.21 | 0 | 1 | BPH | 0 | 0.0256 | 0 | 0.0211 | 0 |
| validation | 738DRE | PCA230627 | 50 | 25.91 | 50 | 25.91 | 50 | 25.91 | 0 | 1 | BPH | 0 | 0.0363 | 0 | 0.0309 | 0 |
| validation | 742DRE | PCA231156 | 50 | 27.34 | 50 | 27.34 | 50 | 27.34 | 0 | 1 | BPH | 0 | 0.0247 | 0 | 0.0203 | 0 |
| validation | 769DRE | PCA231201 | 50 | 27.37 | 50 | 27.37 | 50 | 27.37 | 0 | 1 | BPH | 0 | 0.0245 | 0 | 0.0201 | 0 |
| validation | 749DRE | PCA231157 | 50 | 25.25 | 50 | 25.25 | 50 | 25.25 | 0 | 1 | BPH | 0 | 0.0432 | 0 | 0.0374 | 0 |
| validation | 780DRE | PCA231185 | 50 | 24.9 | 38.24 | 13.14 | 37.09 | 11.99 | 0 | 1 | BPH | 0 | 0.1418 | 0 | 0.2816 | 0 |
| validation | 640DRE | PCA230679 | 50 | 26.83 | 50 | 26.83 | 50 | 26.83 | 0 | 1 | BPH | 0 | 0.0283 | 0 | 0.0236 | 0 |
| validation | 690DRE | PCA230631 | 50 | 27.32 | 35.02 | 12.34 | 50 | 27.32 | 0 | 1 | BPH | 0 | 0.105 | 0 | 0.0754 | 0 |
| validation | 802DRE | PCA231122 | 50 | 26.38 | 50 | 26.38 | 50 | 26.38 | 0 | 1 | BPH | 0 | 0.032 | 0 | 0.0269 | 0 |
| validation | 620DRE | PCA230693 | 50 | 23.55 | 50 | 23.55 | 50 | 23.55 | 0 | 1 | BPH | 0 | 0.0675 | 0 | 0.061 | 0 |
| validation | 710DRE | PCA231094 | 50 | 27.09 | 50 | 27.09 | 50 | 27.09 | 0 | 1 | BPH | 0 | 0.0264 | 0 | 0.0218 | 0 |
| validation | 848DRE | PCA231242 | 50 | 27.63 | 43.27 | 20.9 | 50 | 27.63 | 0 | 1 | BPH | 0 | 0.0443 | 0 | 0.0338 | 0 |
| validation | 804DRE | PCA231132 | 50 | 26.94 | 50 | 26.94 | 50 | 26.94 | 0 | 1 | BPH | 0 | 0.0275 | 0 | 0.0228 | 0 |
| validation | 807DRE | PCA231208 | 50 | 27.39 | 50 | 27.39 | 50 | 27.39 | 0 | 1 | BPH | 0 | 0.0243 | 0 | 0.02 | 0 |
| validation | 747DRE | PCA231128 | 50 | 24.99 | 50 | 24.99 | 50 | 24.99 | 0 | 1 | BPH | 0 | 0.0463 | 0 | 0.0404 | 0 |
| validation | 794DRE | PCA231184 | 50 | 26.63 | 50 | 26.63 | 50 | 26.63 | 0 | 1 | BPH | 0 | 0.0299 | 0 | 0.025 | 0 |
| validation | 638DRE | PCA230668 | 50 | 27.31 | 40.11 | 17.42 | 50 | 27.31 | 0 | 1 | BPH | 0 | 0.0654 | 0 | 0.0489 | 0 |
| validation | 659DRE | PCA230677 | 50 | 26.88 | 50 | 26.88 | 50 | 26.88 | 0 | 1 | BPH | 0 | 0.0279 | 0 | 0.0232 | 0 |
| validation | 684DRE | PCA231139 | 50 | 26.4 | 50 | 26.4 | 50 | 26.4 | 0 | 1 | BPH | 0 | 0.0318 | 0 | 0.0267 | 0 |
| validation | 693DRE | PCA230635 | 50 | 26.95 | 50 | 26.95 | 50 | 26.95 | 0 | 1 | BPH | 0 | 0.0274 | 0 | 0.0227 | 0 |
| validation | 692DRE | PCA230634 | 40.75 | 18.05 | 35.54 | 12.84 | 50 | 27.3 | 0 | 1 | BPH | 0 | 0.3617 | 0 | 0.1961 | 0 |
| validation | 679DRE | PCA231138 | 50 | 26.72 | 35.66 | 12.38 | 50 | 26.72 | 0 | 1 | BPH | 0 | 0.1149 | 0 | 0.0844 | 0 |
| validation | 803DRE | PCA231131 | 50 | 26.14 | 48.05 | 24.19 | 50 | 26.14 | 0 | 1 | BPH | 0 | 0.0413 | 0 | 0.0343 | 0 |
| validation | 704DRE | PCA231145 | 50 | 27.87 | 35.85 | 13.72 | 50 | 27.87 | 0 | 1 | BPH | 0 | 0.0847 | 0 | 0.0601 | 0 |
| validation | 842DRE | PCA231254 | 50 | 27 | 36.41 | 13.41 | 38.19 | 15.19 | 0 | 1 | BPH | 0 | 0.1001 | 0 | 0.1824 | 0 |
| validation | 668DRE | PCA230687 | 50 | 26.39 | 35.85 | 12.24 | 50 | 26.39 | 0 | 1 | BPH | 0 | 0.1224 | 0 | 0.091 | 0 |
| validation | 778DRE | PCA231176 | 50 | 27.69 | 50 | 27.69 | 50 | 27.69 | 0 | 1 | BPH | 0 | 0.0224 | 0 | 0.0183 | 0 |
| validation | 725DRE | PCA231150 | 50 | 25.35 | 50 | 25.35 | 50 | 25.35 | 0 | 1 | BPH | 0 | 0.0421 | 0 | 0.0364 | 0 |
| validation | 811DRE | PCA231247 | 35.39 | 10.74 | 37.12 | 12.47 | 35.64 | 10.99 | 2 | 1 | PCA | 1 | 0.6796 | 1 | 0.722 | 1 |
| validation | 683DRE | PCA231137 | 37.11 | 11.66 | 34.33 | 8.88 | 36.11 | 10.66 | 2 | 0 | PCA | 1 | 0.7225 | 1 | 0.7681 | 1 |
| validation | 826DRE | PCA231100 | 35.81 | 12.77 | 38.45 | 15.41 | 37.16 | 14.12 | 4 | 1 | PCA | 1 | 0.524 | 1 | 0.5404 | 1 |
| validation | 828DRE | PCA231256 | 50 | 26.64 | 38.95 | 15.59 | 38.03 | 14.67 | 4 | 1 | PCA | 1 | 0.0866 | 0 | 0.1667 | 0 |
| validation | 744DRE | PCA231114 | 37.55 | 14.14 | 37.63 | 14.22 | 44.5 | 21.09 | 1 | 1 | PCA | 1 | 0.4943 | 1 | 0.3752 | 1 |
| validation | 713DRE | PCA230601 | 50 | 24.39 | 41.59 | 15.98 | 50 | 24.39 | 2 | 1 | PCA | 1 | 0.1192 | 0 | 0.0979 | 0 |
| validation | 824DRE | PCA231209 | 35.37 | 10.81 | 33.65 | 9.09 | 36.36 | 11.8 | 3 | 1 | PCA | 1 | 0.7474 | 1 | 0.7655 | 1 |
| validation | 660DRE | PCA230686 | 29.41 | 6.32 | 28.59 | 5.5 | 30.31 | 7.22 | 5 | 1 | PCA | 1 | 0.9037 | 1 | 0.9216 | 1 |
| validation | 786DRE | PCA231158 | 31.23 | 4.83 | 30.68 | 4.28 | 33.16 | 6.76 | 4 | 1 | PCA | 1 | 0.9324 | 1 | 0.9426 | 1 |
| validation | 686DRE | PCA231149 | 38.93 | 16.01 | 39.53 | 16.61 | 35.62 | 12.7 | 1 | 1 | PCA | 1 | 0.3556 | 0 | 0.4449 | 1 |
| validation | 789DRE | PCA231169 | 37.26 | 10.85 | 37.48 | 11.07 | 37.74 | 11.33 | 2 | 1 | PCA | 1 | 0.7059 | 1 | 0.7386 | 1 |
| validation | 667DRE | PCA230681 | 50 | 26.88 | 36.04 | 12.92 | 50 | 26.88 | 3 | 1 | PCA | 1 | 0.1067 | 0 | 0.0782 | 0 |
| validation | 735DRE | PCA231109 | 34.08 | 7.54 | 38.84 | 12.3 | 32.23 | 5.69 | 5 | 1 | PCA | 1 | 0.791 | 1 | 0.8617 | 1 |
| validation | 643DRE | PCA230663 | 50 | 27.44 | 35.63 | 13.07 | 50 | 27.44 | 4 | 1 | PCA | 1 | 0.0963 | 0 | 0.0692 | 0 |
| validation | 814DRE | PCA231155 | 31.5 | 5.78 | 30.01 | 4.29 | 31.13 | 5.41 | 2 | 1 | PCA | 1 | 0.9211 | 1 | 0.9427 | 1 |
| validation | 751DRE | PCA230629 | 50 | 26.33 | 37.08 | 13.41 | 50 | 26.33 | 5 | 1 | PCA | 1 | 0.1112 | 0 | 0.0835 | 0 |
| validation | 770DRE | PCA231204 | 39.41 | 16.59 | 36.12 | 13.3 | 37.56 | 14.74 | 5 | 1 | PCA | 1 | 0.4113 | 0 | 0.4576 | 1 |
| validation | 771DRE | PCA231206 | 33.67 | 10.88 | 33.23 | 10.44 | 33.43 | 10.64 | 3 | 1 | PCA | 1 | 0.718 | 1 | 0.7601 | 1 |
| validation | 797DRE | PCA231203 | 50 | 26.85 | 50 | 26.85 | 50 | 26.85 | 2 | 1 | PCA | 1 | 0.0282 | 0 | 0.0234 | 0 |
| validation | 836DRE | PCA231211 | 37.95 | 14.95 | 35.31 | 12.31 | 34.01 | 11.01 | 5 | 1 | PCa | 1 | 0.5075 | 1 | 0.6105 | 1 |
| validation | 792DRE | PCA231178 | 35.6 | 12.35 | 36.21 | 12.96 | 38.32 | 15.07 | 3 | 1 | PCA | 1 | 0.6034 | 1 | 0.5875 | 1 |
| validation | 681DRE | PCA231153 | 30.32 | 6.85 | 31.03 | 7.56 | 31.65 | 8.18 | 4 | 1 | PCA | 1 | 0.8739 | 1 | 0.8935 | 1 |
| validation | 830DRE | PCA231260 | 38.1 | 11.34 | 34.86 | 8.1 | 38.16 | 11.4 | 5 | 1 | PCA | 1 | 0.7489 | 1 | 0.7761 | 1 |
| validation | 707DRE | PCA231041 | 29.96 | 5.54 | 34.78 | 10.36 | 30.43 | 6.01 | 5 | 1 | PCA | 1 | 0.8676 | 1 | 0.9024 | 1 |
| validation | 731DRE | PCA230624 | 27.57 | 1.84 | 26.47 | 0.74 | 27.79 | 2.06 | 3 | 1 | PCA | 1 | 0.971 | 1 | 0.9802 | 1 |
| validation | 743DRE | PCA231161 | 34.27 | 8.65 | 31.4 | 5.78 | 34.5 | 8.88 | 5 | 1 | PCA | 1 | 0.8583 | 1 | 0.8815 | 1 |
| validation | 827DRE | PCA231105 | 36.13 | 10.47 | 35.53 | 9.87 | 37.93 | 12.27 | 5 | 1 | PCA | 1 | 0.7436 | 1 | 0.7526 | 1 |
| validation | 645DRE | PCA230688 | 32.57 | 6.7 | 32.32 | 6.45 | 32.83 | 6.96 | 4 | 1 | PCA | 1 | 0.8885 | 1 | 0.9133 | 1 |
| validation | 673DRE | PCA230649 | 50 | 23.66 | 50 | 23.66 | 50 | 23.66 | 5 | 1 | PCA | 1 | 0.0656 | 0 | 0.0591 | 0 |
| validation | 815DRE | PCA231250 | 38.18 | 11.88 | 42.15 | 15.85 | 50 | 23.7 | 2 | 1 | PCA | 1 | 0.5517 | 1 | 0.3521 | 1 |
| validation | 823DRE | PCA231179 | 32.8 | 8.78 | 33.14 | 9.12 | 33.11 | 9.09 | 5 | 1 | PCA | 1 | 0.8081 | 1 | 0.8413 | 1 |
| validation | 621DRE | PCA230695 | 50 | 26.73 | 35.41 | 12.14 | 36.9 | 13.63 | 3 | 1 | PCA | 1 | 0.1172 | 0 | 0.2289 | 0 |
| validation | 702DRE | PCA231141 | 29.85 | 5.36 | 29.52 | 5.03 | 32.91 | 8.42 | 4 | 1 | PCA | 1 | 0.9209 | 1 | 0.9255 | 1 |
| validation | 700DRE | PCA231117 | 33.27 | 9.99 | 33.85 | 10.57 | 33.13 | 9.85 | 3 | 1 | PCA | 1 | 0.746 | 1 | 0.7892 | 1 |
| validation | 806DRE | PCA231197 | 40.1 | 15.56 | 50 | 25.46 | 50 | 25.46 | 1 | 1 | PCA | 1 | 0.1949 | 0 | 0.1098 | 0 |
| validation | 734DRE | PCA231086 | 42.87 | 19.75 | 35.33 | 12.21 | 50 | 26.88 | 5 | 1 | PCA | 1 | 0.3096 | 0 | 0.1787 | 0 |
| validation | 835DRE | PCA231272 | 34.84 | 11.74 | 33.02 | 9.92 | 35.81 | 12.71 | 3 | 1 | PCA | 1 | 0.6978 | 1 | 0.7136 | 1 |
| validation | 676DRE | PCA230661 | 31.25 | 5.05 | 29.51 | 3.31 | 31.25 | 5.05 | 4 | 1 | PCA | 1 | 0.9361 | 1 | 0.9531 | 1 |
| validation | 753DRE | PCA231183 | 50 | 25.25 | 50 | 25.25 | 38.22 | 13.47 | 1 | 1 | PCA | 1 | 0.0432 | 0 | 0.0986 | 0 |
| validation | 721DRE | PCA231126 | 31.77 | 8.06 | 30.41 | 6.7 | 31.85 | 8.14 | 2 | 1 | PCA | 1 | 0.8595 | 1 | 0.887 | 1 |
| validation | 671DRE | PCA230620 | 24.59 | 1.56 | 24.14 | 1.11 | 25.53 | 2.5 | 5 | 1 | PCA | 1 | 0.9713 | 1 | 0.9795 | 1 |
| validation | 832DRE | PCA231257 | 34.83 | 9.79 | 35.51 | 10.47 | 35.32 | 10.28 | 4 | 1 | PCA | 1 | 0.7545 | 1 | 0.7885 | 1 |
| validation | 712DRE | PCA231093 | 35.65 | 10.78 | 34.64 | 9.77 | 36.51 | 11.64 | 5 | 1 | PCA | 1 | 0.7351 | 1 | 0.7574 | 1 |
| validation | 741DRE | PCA231134 | 37.53 | 9.18 | 36.33 | 7.98 | 36.84 | 8.49 | 4 | 1 | PCA | 1 | 0.8152 | 1 | 0.8551 | 1 |
| validation | 766DRE | PCA231194 | 25.85 | 3.36 | 26.03 | 3.54 | 26.52 | 4.03 | 5 | 1 | PCA | 1 | 0.9506 | 1 | 0.964 | 1 |
| validation | 777DRE | PCA231172 | 36.18 | 12.89 | 24.02 | 0.73 | 26.32 | 3.03 | 4 | 1 | PCA | 1 | 0.8282 | 1 | 0.9212 | 1 |
| validation | 796DRE | PCA231195 | 30.83 | 7.6 | 28.36 | 5.13 | 32.76 | 9.53 | 3 | 1 | PCA | 1 | 0.8861 | 1 | 0.8945 | 1 |
| validation | 821DRE | PCA231175 | 32.01 | 8.06 | 30.23 | 6.28 | 32.35 | 8.4 | 3 | 1 | PCA | 1 | 0.8646 | 1 | 0.8885 | 1 |
| validation | 825DRE | PCA231236 | 34.03 | 7.82 | 32.07 | 5.86 | 35.52 | 9.31 | 4 | 1 | PCA | 1 | 0.8742 | 1 | 0.8873 | 1 |
| validation | 642DRE | PCA230685 | 31 | 4.82 | 31.23 | 5.05 | 31.33 | 5.15 | 5 | 1 | PCA | 1 | 0.9274 | 1 | 0.9464 | 1 |
| validation | 812DRE | PCA230655 | 26.67 | 2.05 | 25.05 | 0.43 | 26.68 | 2.06 | 5 | 1 | PCA | 1 | 0.9708 | 1 | 0.9803 | 1 |
| validation | 680DRE | PCA231146 | 35.74 | 10.67 | 36.48 | 11.41 | 34.91 | 9.84 | 3 | 1 | PCA | 1 | 0.7053 | 1 | 0.7615 | 1 |
| validation | 646DRE | PCA230676 | 31.77 | 8.23 | 31.56 | 8.02 | 32.2 | 8.66 | 2 | 1 | PCA | 1 | 0.8384 | 1 | 0.8669 | 1 |
| validation | 687DRE | PCA231154 | 28.07 | 5.4 | 26.46 | 3.79 | 28.25 | 5.58 | 5 | 1 | PCA | 1 | 0.9292 | 1 | 0.9467 | 1 |
| validation | 760DRE | PCA231050 | 33.47 | 10.68 | 33.52 | 10.73 | 33.44 | 10.65 | 5 | 1 | PCA | 1 | 0.7191 | 1 | 0.7596 | 1 |
| validation | 724DRE | PCA231148 | 34.8 | 8.06 | 31.21 | 4.47 | 33.03 | 6.29 | 4 | 1 | PCA | 1 | 0.8848 | 1 | 0.9188 | 1 |
| validation | 746DRE | PCA231125 | 30.39 | 7.45 | 28.99 | 6.05 | 31.75 | 8.81 | 5 | 1 | PCA | 1 | 0.8791 | 1 | 0.8943 | 1 |
| validation | 706DRE | PCA231000 | 28.32 | 2.12 | 27.17 | 0.97 | 28.73 | 2.53 | 4 | 1 | PCA | 1 | 0.9689 | 1 | 0.9782 | 1 |
| validation | 662DRE | PCA231004 | 50 | 26.09 | 26.29 | 2.38 | 28.15 | 4.24 | 4 | 1 | PCA | 1 | 0.2867 | 0 | 0.6407 | 1 |
| validation | 761DRE | PCA231089 | 33.3 | 10.72 | 32.02 | 9.44 | 33.13 | 10.55 | 5 | 1 | PCA | 1 | 0.7436 | 1 | 0.7811 | 1 |

2 gene: ADD3_2 and GSX2_2; 3gene: **ADD3_2, GSX2_2, and CYBA_1**

**Table S13. The experimental materials, reagents, and instruments involved in this research.**

| **Project Name** | **Material Type** | **Material Name** | **Manufacturer** | **Catalog Number** |
| --- | --- | --- | --- | --- |
| Methylation Amplicon Library Construction | Reagent | Amplicon Library Construction Primer Set | Generic | / |
| Methylation Amplicon Library Construction | Reagent | Jupiter rTaq Multiplex 2X Master Mix | Changzhou Xinyisheng Life Science & Technology Co., Ltd. | LS-EZ-K-00003O |
| Methylation Detection | Reagent | Blood/Cell/Tissue Genomic DNA Extraction Kit | Tiangen Biotech (Beijing) Co., Ltd. | DP304-03 |
| Methylation Detection | Reagent | Nucleic Acid Extraction or Purification Reagent | Xingchun (Changzhou) Biotechnology Co., Ltd. | M15R-2 |
| Methylation Detection | Reagent | EZ DNA Methylation—Lightning™ Kit | Zymo Research Corporation | D5031 |
| Methylation Detection | Reagent | Hieff Unicon Universal TaqMan Multiplex qPCR Master Mix | Yeasen Biotechnology (Shanghai) Co., Ltd. | YS11211ES08 |
| Methylation Detection | Reagent | qPCR Detection Primer Set | Generic | / |
| Methylation Detection | Reagent | AR Grade Anhydrous Ethanol | Huihong | / |
| Methylation Detection | Reagent | Nuclease-Free Water | Yeasen Biotechnology (Shanghai) Co., Ltd. | 10601ES76 |
| Methylation Detection | Reagent | Agarose | Thermo Fisher | 75510019 |
| Methylation Detection | Reagent | 50× TAE Solution | Yeasen Biotechnology (Shanghai) Co., Ltd. | 60116ES76 |
| Methylation Detection | Reagent | GelstainRed™ Nucleic Acid Dye | UElandy | S2009L |
| Methylation Detection | Reagent | 6× Loading Buffer | TAKARA | 9156 |
| Methylation Detection | Reagent | GoldBand 100 bp DNA Ladder | Yeasen Biotech | 10507ES60 |
| Methylation Detection | Equipment | Mini Centrifuge | Haimen Kylin-Bell Instrument Manufacturing Co., Ltd. | LX-200 |
| Methylation Detection | Equipment | Vortex Mixer | Crystal / Jingqi (USA) Co., Ltd. | VM-01U(HYQ-3110) |
| Methylation Detection | Equipment | Microplate Centrifuge | Tiangen Biotech (Beijing) Co., Ltd. | OSE-MP25 |
| Methylation Detection | Equipment | Heating and Cooling Dry Bath | Cayuid Biotechnology Yixing Co., Ltd. | H203-100C |
| Methylation Detection | Equipment | Qubit 4 Fluorometer | Invitrogen / Life Technologies | Qubit 4.0 |
| Methylation Detection | Equipment | Clean Workbench | Jinan Xinbeixi Biotechnology Co., Ltd. | BBS-DDC |
| Methylation Detection | Equipment | Triple Block Gradient PCR Thermal Cycler | Life Technologies | 3*32-Well |
| Methylation Detection | Equipment | Electrophoresis Power Supply | Tanon / Shanghai Tanon Technology Co., Ltd. | EPS-600 |
| Methylation Detection | Equipment | Multifunctional Horizontal Electrophoresis Tank | Tanon / Shanghai Tanon Technology Co., Ltd. | HE-120 |
| Methylation Detection | Equipment | Blue Light Gel Cutter | Tiangen Biotech (Beijing) Co., Ltd. | OSE-470L |
| Methylation Detection | Equipment | Electronic Balance | Hangzhou Youheng Weighing Equipment Co., Ltd. | HLD-10002 |
| Methylation Detection | Equipment | Fully Automatic Medical PCR Analysis System | Shanghai Hongshi Medical Technology Co., Ltd. | SLAN-96S |
| Methylation Detection | Equipment | Real-Time Fluorescence Quantitative PCR System | Life Technologies | ABI 7500 |
| MeDIP | Reagent | MagMeDIP qPCR Kit / Magnetic Methylated DNA Immunoprecipitation Kit | Diagnode | C02010021 |
| MeDIP | Reagent | NadPrep® DNA Universal Library Preparation Kit (for Illumina®) | Nadoda | 1002103 |
| MeDIP | Reagent | KAPA HiFi HotStart ReadyMix | KAPA BioSystems | KK2602 |
| MeDIP | Reagent | Full-Length Adapter | Generic | / |
| MeDIP | Reagent | Amplification Primers | Generic | / |
| MeDIP | Reagent | AR Grade Anhydrous Ethanol | Huihong | / |
| MeDIP | Reagent | Nuclease-Free Water | Yeasen Biotech | 10601ES76 |
| MeDIP | Reagent | Agarose | Thermo Fisher | 75510019 |
| MeDIP | Reagent | 50× TAE Solution | Yeasen Biotech | 60116ES76 |
| MeDIP | Reagent | GelstainRed™ Nucleic Acid Dye | UElandy | S2009L |
| MeDIP | Reagent | 6× Loading Buffer | TAKARA | 9156 |
| MeDIP | Reagent | GoldBand 100 bp DNA Ladder | Yeasen Biotech | 10507ES60 |
| MeDIP | Equipment | M220 Focused-ultrasonicator | Covaris | 500295 |
| MeDIP | Equipment | Rotating Mixer | Jiemei Jingqi | TR-04U |
| MeDIP | Equipment | Magnetic Stand (Compatible with 200 μL PCR Tubes) | / | / |
| MeDIP | Equipment | Standard PCR Thermal Cycler | Langji |  |
| MeDIP | Equipment | Multi-Range Pipettes | Eppendorf | / |
| MeDIP | Equipment | Qubit 4 Fluorometer | Thermo Fisher | Qubit 4 |
| MeDIP | Equipment | Micro Centrifuge (Compatible with 1.5 mL Tubes and 200 μL PCR Tubes) | Kylin-Bell or Other Brands |  |
| MeDIP | Equipment | Vortex Oscillation Mixer | Kylin-Bell or Other Brands |  |
| MeDIP | Equipment | Green Plus Gel Cutter | Tiangen Biotech | OSE-470L |
| MeDIP | Equipment | EPS-600 Digital Constant Voltage/Current Electrophoresis Power Supply | Shanghai Tanon | EPS-600 |
| MeDIP | Equipment | HE-120 Multifunctional Horizontal Electrophoresis Tank | Shanghai Tanon | HE-120 |
| MeDIP | Equipment | 0.01 g–1000 g Electronic Balance |  |  |
| MeDIP | Equipment | Microwave Oven | Household Use |  |
| MeDIP | Equipment | 100 mL Graduated Cylinder |  |  |
| MeDIP | Equipment | 1 L Blue-Cap Bottle | Shuniu or Other Brands |  |
| Sample Preservation | Reagent | Disposable Urine Collection Device | Yearth Biotechnology Co. Ltd | QS01A |

**Table S14. Consistency of Clinical Classification between Day 0 and Day 5**

|  | Day 5 positive | Day 5 negative | Total |
| --- | --- | --- | --- |
| Day 0 positive | 5 | 0 | 5 |
| Day 0 negative | 0 | 14 | 14 |
| Total | 5 | 14 | 19 |
